# A Light-Promoted Innate Trifluoromethylation of Pyridones and Related *N*-Heteroarenes

**DOI:** 10.1021/acs.orglett.3c01710

**Published:** 2023-06-28

**Authors:** Ashley Dang-Nguyen, Kristine C. Legaspi, Connor T. McCarty, Diane K. Smith, Jeffrey Gustafson

**Affiliations:** Department of Chemistry and Biochemistry, San Diego State University, 5500 Campanile Drive, San Diego, California 92182-1030, United States

## Abstract

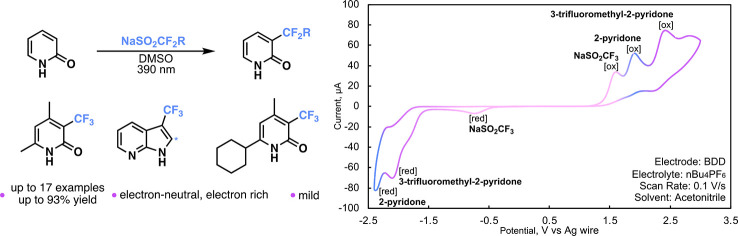

We report a practical, light-mediated perfluoroalkylation using Langlois’ reagent (sodium trifluoromethylsulfinate) that proceeds in the absence of any photocatalyst or additives. This method has allowed for the facile functionalization of pyridones and related *N*-heteroarenes such as azaindole. This protocol is operationally simple, uses readily available materials, and is tolerable for electron-neutral and -rich functional pyridones. Cyclic voltammetry was utilized as a mechanistic probe, and preliminary data suggest the reaction may involve an electrophilic radical mechanism.

Perfluoroalkyl groups impart unique electronic effects to small molecules, which has led to their widespread incorporation in said molecules.^1,2^ In drug discovery, these groups have been shown to increase metabolic stability, lipophilicity, and overall activity.^3^ Perfluoroalkylated functionalities are often added to arenes and *N*-heteroarenes, necessitating the development of strategies for further incorporating these groups. Recent developments in perfluoroalkylation methods have been employed by reagents such as, but not limited to, Prakash’s reagent,^4^ Langlois’ reagent,^5^ Togni’s reagent,^6^ Umemoto’s reagent,^7^ Baran’s reagent,^8^ Ritter’s reagent,^9^ CF_3_I,^10^ triflyl chloride,^11^ triflic anhydride,^12^ and trifluoroacetic acid (TFA).^13^ These reagents can be either oxidized or reduced to generate a perfluoroalkyl radical with electrophilic or nucleophilic behavior.^14−16^ While many of the methods developed alongside these reagents have been shown to functionalize a range of *N*-heteroarenes, they often require harsh conditions, precious metal catalysts, or prefunctionalization of substrates, which can limit their practicality on a large scale and limit their utility for late-stage functionalization (LSF) of more complex *N*-heteroarenes.

There are fewer methodologies that afford pyridone perfluoroalkylation compared with other arenes and heteroarenes. Pyridones are a privileged scaffold and are common bioisosteres for a variety of functional groups such as amides, pyranones, pyrimidines, pyrazines, and phenols.^17−19^ There are several biologically relevant perfluoroalkylated pyridones, including FDA-approved drug Pifeltro, an HIV-1 medication for use in combination with other antiretroviral medicines, and Fusilade DX, an EPA-approved herbicide used to treat weeds for cotton and soybeans. Despite their ubiquity, there are few direct, mild synthetic methods toward trifluoromethylated pyridones. They are often synthesized on a scale from the corresponding methylated pyridine, which then undergoes a chlorination and subsequent fluorination before transforming it into a pyridone (Scheme 1a).^20−22^ In the context of Pifeltro, the perfluoroalkyl group was preinstalled onto a halogenated pyridine, which was subsequently reacted in an S_N_Ar fashion followed by oxidation to produce the trifluoromethylated pyridone moiety.^23^ Another common route for achieving fluorinated pyridones involves a deoxyfluorination from the corresponding hydroxypicolinic acids from the Mykhailiuk^24^ group (Scheme 1b). Direct strategies for incorporating perfluoroalkyl groups onto pyridones are highly desirable.^25^ Current strategies for directly obtaining trifluoromethylated pyridones and similar scaffolds include an iron(II)-mediated trifluoromethylation using CF_3_I and hydrogen peroxide from the Yamakawa^10^ group (Scheme 1c), photoredox trifluoromethylations using triflyl chloride from the MacMillan^11^ group (Scheme 1d), and TFA from the Jin^13^ group (Scheme 1e). Herein, we report a simple light-mediated radical trifluoromethylation strategy that circumvents the need for harsh, expensive conditions that do not use any photocatalyst or oxidative additive on pyridones (Scheme 1f). This methodology has also been extended to include *N*-heteroarenes as well as some examples of late-stage functionalization (LSF).

**Scheme 1:**
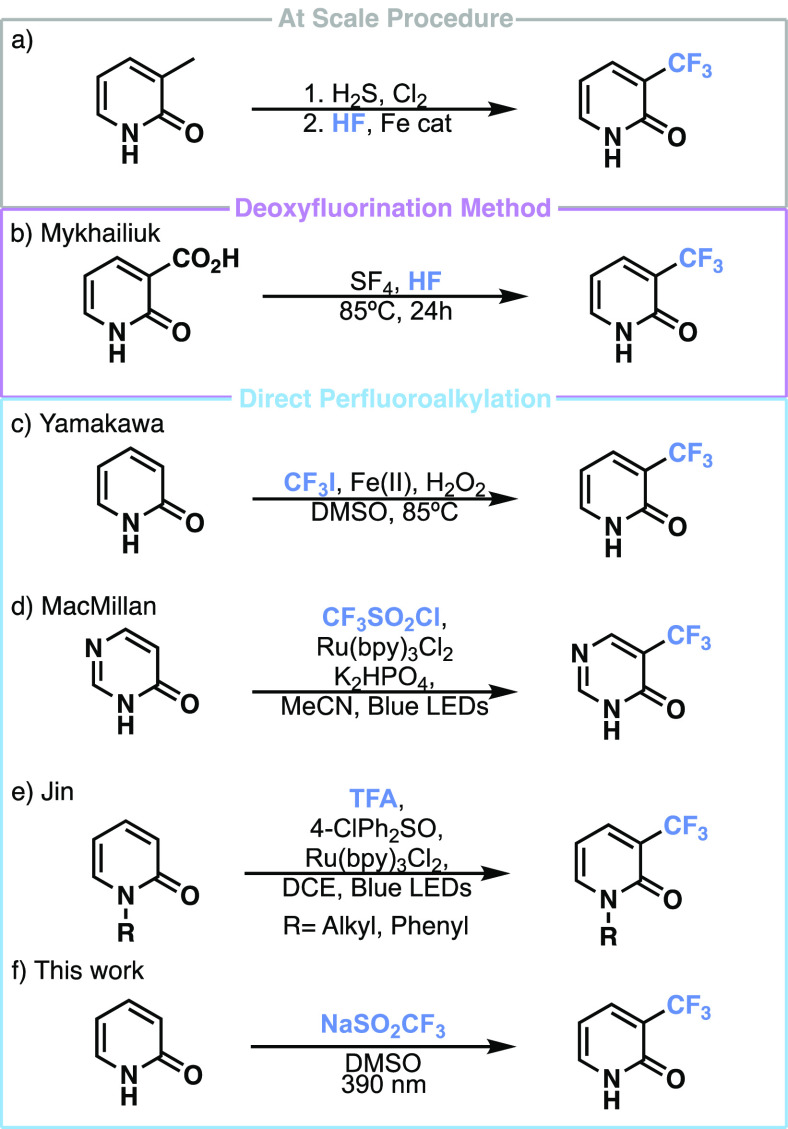
Preparation of Trifluoromethylated Pyridones

While studying the perfluoroalkylation of aromatics, we noticed a unique reactivity where pyridones such as **1a** could undergo a trifluoromethylation at position three using Langlois’ reagent **2a**, DMSO, and 390 nm LEDs (Table 1, entry 1) without the need for any photocatalyst or additive under an ambient atmosphere. The functionalization is occurring at the most nucleophilic site on **1a**, and we postulate that this proceeds through an electrophilic perfluoroalkylation mechanism.^26^ We found this noteworthy as there was no need for any photocatalyst or terminal oxidant, as is typically needed for such transformations. In the absence of light, we observe no conversion (Table 1, entry 2); however, reactivity can be regained in the absence of light by using K_2_S_2_O_8_ as a terminal oxidant (Table 1, entry 3), which would be expected on the basis of work by Baran and co-workers.^14^ We observed moderate solvent effects; for example, a 1:1 MeCN/H_2_O mixture (Table 1 entry 4) resulted in a decrease in conversion. However, DMF (Table 1, entry 5) gave nearly indistinguishable conversions from DMSO. When the wavelength of light was changed from 390 to 440 nm (Table 1, entry 6) or 467 nm (Table 1, entry 7), we observed a continual decrease in conversion. When the reaction contents are sparged with argon (Table 1, entry 8), we notice a decrease in conversion; however, upon sparging with O_2_ (Table 1 entry 9), we noticed a large effect compared to the argon results, suggesting the importance of oxygen in this reaction. With regard to the sulfinate used, the metal counterion had a weak effect on reactivity as **2b** can be used, albeit resulting in lower yields despite a larger number of equivalents (Table 1, entry 10). The corresponding methyl sulfinate **2c** resulted in no conversion, suggesting the need for electron poor sulfinates (Table 1, entry 11). Upon using sodium difluorosulfinate **2d** (Table 1, entry 12), we noticed a significant decrease in conversion compared to **2a**, possibly due to the decrease in electrophilicity of the CF_2_H radical in comparison.^27^

**Table 1:**
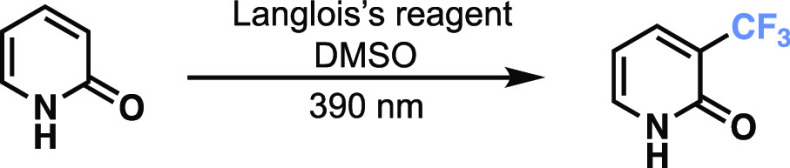
Optimization of the Reaction Conditions^a^

entry	deviations from the standard conditions	% conversion^b^
1	standard conditions	96
2	no light	ND^c^
3	K_2_S_2_O_8_ additive (3.0 equiv), no light	95
4	MeCN/H_2_O (1:1)	60
5	DMF	90
6	440 nm instead of 390 nm	65
7	467 nm instead of 390 nm	60
8	sparge with Ar, freeze–pump–thaw	54
9	sparge with O_2_	98
10	Zn(SO_2_CF_3_) (**2b**, 1.4 equiv)	80
11	NaSO_2_CH_3_ (**2c**)	ND^c^
12	NaSO_2_CF_2_H (**2d**)	17

a Conditions: **1** (0.03125 mmol, 1 equiv), sulfinate (0.0625 mmol, 2.0 equiv), dDMSO (0.5 mL), RT, 24h, stir bar.

b Conversion based on ^1^H NMR analysis using 0.03125 mmol trimethoxybenzene (TMB) as an internal standard.

c Not determined.

We next evaluated the optimal conditions across a variety of pyridones to demonstrate the versatility of this approach. As shown in Scheme 2, the reaction is tolerable among different pyridones, including aryl-alkylated (**3b** and **3c**) and *N*-alkylated pyridones (**3e** and **3f**) in good yields ranging from 56% to 93%. Interestingly, when position 3 is methylated, there is a considerably lower yield (12%) of the 5-trifluoromethylated product (**3d**), versus 19% when the reaction mixture is sparged with O_2_, which suggests that the electronics of the substrate are important in determining whether the reaction can occur. Swapping Langlois’ reagent for other difluorosulfinate salts such as NaSO_2_CF_2_CH_3_ (**2c**) resulted in a 90% yield of **3h**. NaSO_2_CF_2_H (**2d**) resulted in a 9% yield of **3h**. A yield of 15% was achieved when the reaction mixture was sparged with O_2_. NaSO_2_CF_2_C_8_H_6_Br (**2e**) resulted in a 79% yield of **3i**. 4-Pyridone, another bioactive pyridone scaffold, was also subjected to this methodology and produced a mixture of mono- and ditrifluoromethylated products in 51% (**3j**) and 23% (**3k**) yields, respectively. While we initially thought that pyridones were unique to this methodology, we were pleasantly surprised that other *N*-heteroarenes were also perfluoroalkylated. We were able to obtain trifluoromethylated uracil (**3l**) and azaindole (**3m**) in 60% and 70% yields, respectively. 2-Quinazoline was also perfluoroalkylated using these conditions, generating product **3n** in ≤65% yield. We evaluated different pyridone-containing ligands as seen in the literature for use in catalysis,^28−30^ and we obtained **3o** and **3p** in moderate yields of 49% and 42%, respectively. We did observe some substrate limitations, as we were unable to perfluoroalkylate 5-nitro-2-pyridone **1q** and mesitylene **1r**. We believe that in the case of **1q**, the pyridone ring is too electron poor due to the nitro substituent. However, in the context of **1r**, it appears the photocatalyst and oxidant-free chemistry necessitate a heteroatom bearing lone pairs, which is believed to interact with the sulfinate (*vide infra*) despite sparging the reaction mixture with O_2_, which we note has a positive effect on yields.

**Scheme 2:**
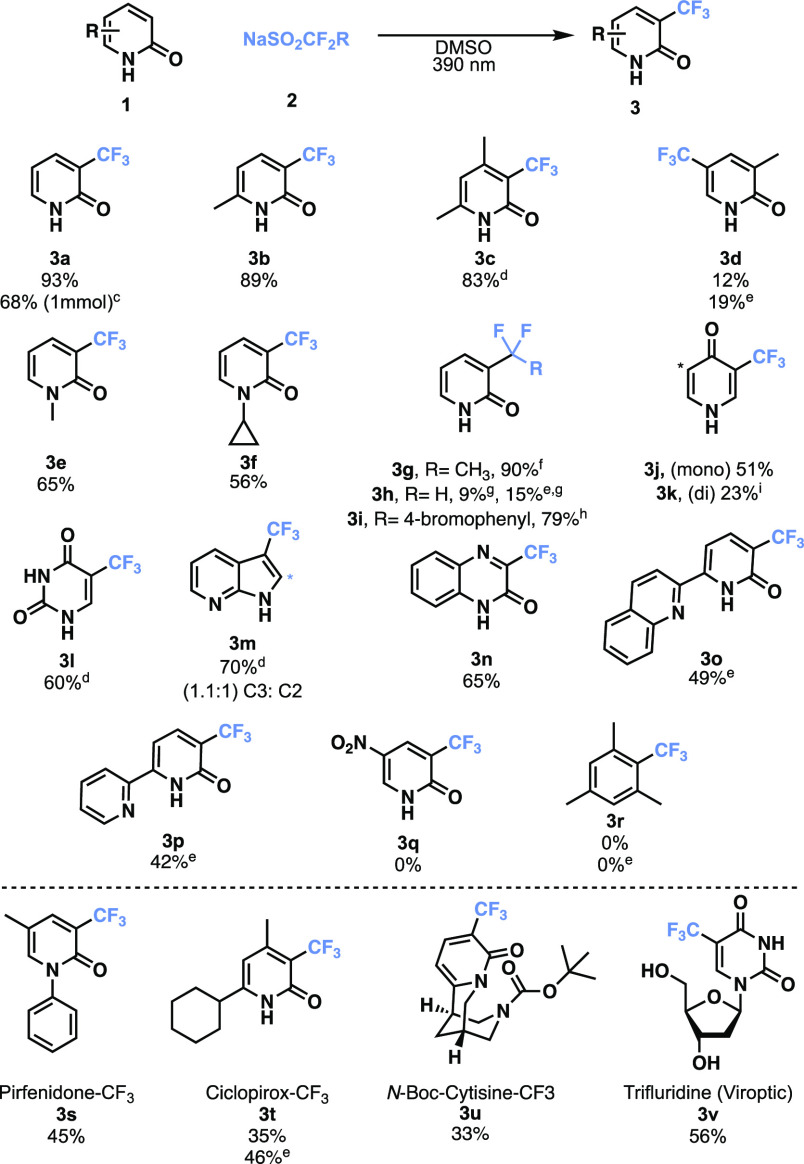
Substrate Scope of Trifluoromethylation Pyridones and Related *N*-Heteroarenes, Conditions: **1** (0.125 mmol, 1 equiv), sulfinate (0.25 mmol, 2.0 equiv), DMSO (2 mL), rt, 24 h. Isolated yield. On a 1 mmol scale using 2.0 equiv of **2a**, sparged with O_2_. With 1.5 equiv of **2a**. Sparged with O_2_. NaSO_2_CF_2_CH_3_ (**2c**), 2.0 equiv. NaSO_2_CF_2_H (**2d**), 2.0 equiv. NaSO_2_CF_2_C_8_H_6_Br (**2e**), 2.0 equiv. With 3.0 equiv of **2a**.

Inspired by the ability to perfluoroalkylate pyridone-containing ligands, we believed that this methodology would also be suitable for the late-stage functionalization of bioactive molecules. To evaluate this strategy, we selected bioactive pyridones, including pirfenidone (**1s**), used in the treatment of idiopathic pulmonary fibrosis, cicloprox (**1t**), used as a treatment for fungal infections, cytisine (**1u**), a former therapy for smoking cessation in Europe and Asia, and trifluridine (**1v**), an ophthalmic treatment for viral infections. We were able to perfluoroalkylate these bioactive molecules in isolated yields of 45% (**3s**), 35% (**3t**), 33% (**3u**), and 56% (**3v**), respectively. We were able to increase the yield of **3t** when the reaction mixture was sparged with oxygen to 46%. In the case of **1u**, the secondary amine was identified to be an issue; however, upon Boc protection, we were able to produce **3u** in 33% yield.

To gain more insight into the reaction mechanism, we performed the following experiments to elucidate the order of transformation. First, upon addition of TEMPO to the reaction, no product **3a** was formed, suggesting that the trifluoromethylation occurred via a radical pathway (Scheme 3a).^31^ Next, we wanted to investigate the role of the light by performing a light on/off experiment to determine whether the reaction occurs through a chain propagation mechanism and whether it could be initiated in the absence of light.^32^ The reaction was sluggish using 20 min intervals, which were increased to hourly intervals. We observed that when light was removed, the reaction ceased and would occur only in the presence of light (Scheme 3b). To probe the formation of a charge-transfer complex, all reagents were then analyzed using UV–vis spectroscopy and were found to absorb in the UV-A and UV-B regions (see the Supporting Information). The reaction mixture was measured, and no significant bathochromic shift was observed, suggesting another mode of radical generation, possibly via n, π* as postulated by Mi, Li, and co-workers.^33^ To better visualize the reaction, we performed cyclic voltammetry (CV) experiments to observe the presence of all reagents in the reaction mixture and to study their redox potentials. We found that pyridone **1a** has an oxidative potential of 1.94 V and a reductive potential of −2.16 V (page S108 of the Supporting Information) and Langlois’ reagent **2a** has oxidative and reductive potentials of 1.65 and −0.76 V, respectively (page S109 of the Supporting Information). We were unable to introduce DMSO in the CVs to study its effect, as DMSO is oxidized at the same potential as **2a**, which prevented analysis. When both reagents are in solution, we can see distinct peaks depending on the concentration; 100 mM was optimal for irradiation, and all CV was performed at an overall concentration of 1 mM (Scheme 3c). At 0 h, we observe an ∼1:1 ratio of reagents; however, after irradiation of the solution at 1 h, we notice a significant decrease in relative peaks between **1a** and **2a**, suggesting the consumption of **2a** initially. This is in line with finding from the Kim^34^ group that **2a** would be easier to oxidize than **1a**. When comparing the data at 7 and 15 h, we notice a decrease in the levels of both **1a** and **2a**, and the distinct formation of **3a**, which has an oxidative potential of 2.41 V and a reductive potential of −1.83 V (page S109 of the Supporting Information). On the basis of our experimental probes, we postulate that **2a** undergoes a light-mediated oxidation to generate a CF_3_ radical, which then reacts with the *N*-aryl substrate, generating a trifluoromethylated intermediate bearing a radical, which then undergoes oxidation to the cationic product, followed by rearomatization. On the basis of the results in Table 1 (entries 8 and 9) we note the dependence of oxygen; thus, it may be plausible that these conditions are exciting oxygen, which can then oxidize **2a** or participate in the oxidation to rearomatize the product.^35,36^ Further mechanistic studies to better understand the full breadth of the mechanism are currently underway and will be the subject of future work.

**Scheme 3:**
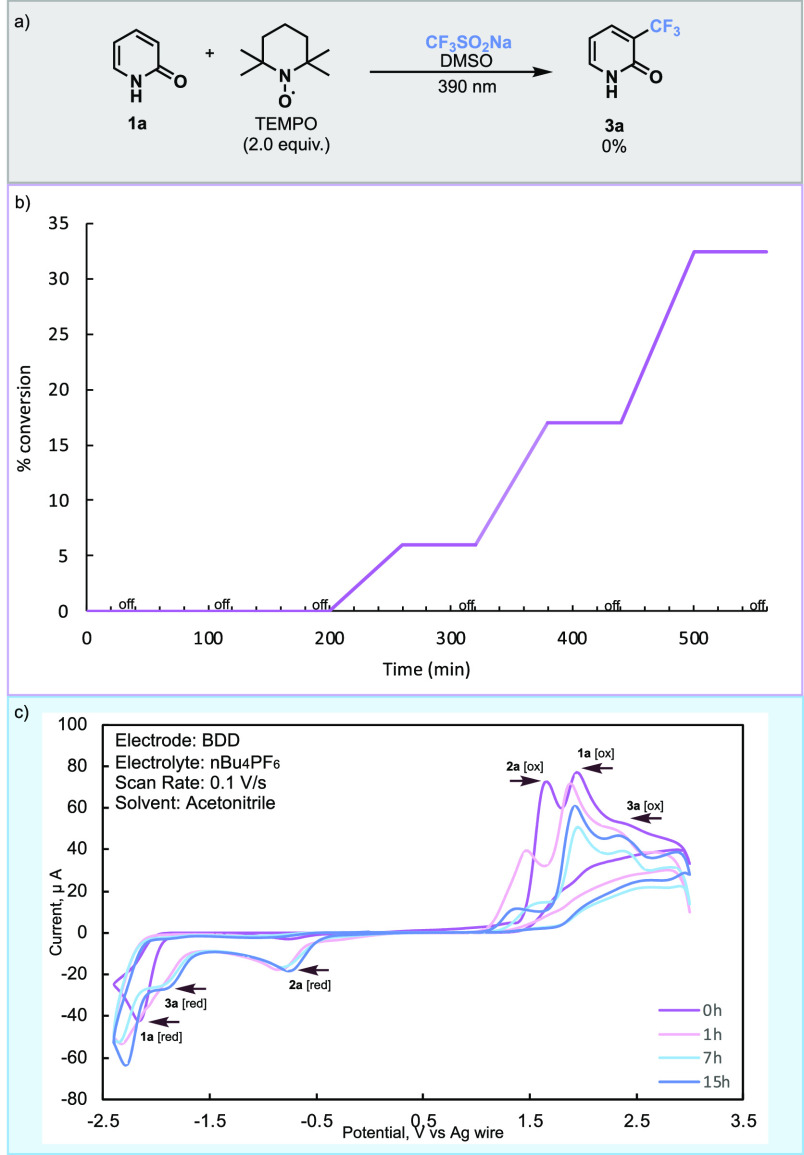
Mechanistic Studies (a) Radical trapping experiment using the same number of equivalents of sulfinate and TEMPO. (b) On/off experiment, % conversion based on ^19^F NMR analysis using 0.03125 mmol of TMB as an internal standard. (c) Cyclic voltammetry. The plotting convention is that of IUPAC. The working electrode is boron-doped diamond (BDD). The counter electrode is platinum. The reference electrode is silver. A 100 mM solution of **1a** and **2a** (1.1 equiv) was irradiated at different time points and analyzed at 1 mM.

In summary, we have described a new, light-promoted method for the trifluoromethylation of pyridones and related *N*-heteroarenes that does not need any photocatalyst or oxidant. This methodology is also inclusive of using a variety of sulfinate salts bearing two or more fluorine substituents. This approach is operationally simple and minimally affected by inert gases or water in the reaction. While Langlois’ reagent can generate a CF_3_ radical via reduction or oxidation, we offer evidence suggesting an oxidative mechanism for the electrophilic trifluoromethylation of pyridones and related *N*-heteroarenes.

## Data Availability

The data underlying this study are available in the published article and its Supporting Information.

## Supporting Information Available

Experimental procedures, characterization of all new and existing compounds, UV–vis, cyclic voltammetry details, and their corresponding NMR and HRMS spectra (PDF)

## Dedication

The authors dedicate this paper to our dear collaborator Dr. Diane K. Smith (1960–2022).
