# Supplementary material for: A Light-Promoted Innate Trifluoromethylation of Pyridones and Related N-Heteroarenes
Source: Org Lett. 2023 Jun 28;25(26):4898–902. doi: 10.1021/acs.orglett.3c01710 (PMC10334463; doi:10.1021/acs.orglett.3c01710)

Supporting Information

A light-promoted innate trifluoromethylation of pyridones and related *N*-heteroarenes

Ashley Dang-Nguyen[a], Kristine C. Legaspi[a], Connor T. McCarty[a], Diane K. Smith[a], and Jeffrey Gustafson*[a]

[a]Department of Chemistry and Biochemistry, San Diego State University, 5500 Campanile Drive, San Diego, California 92182-1030, United States

Corresponding Author: Jeffrey L. Gustafson, jgustafson@sdsu.edu

Table of Contents

A. GENERAL INFORMATION 6

A1. NMR: 6
A2. Mass Spec: 6
A3. Reagents/Purification: 6
A4. Photoredox: 6
A5. Common Abbreviations Used: 6

B. LIGHT SET UP 9

C. OPTIMIZATION 10

D. GENERAL PROCEDURES 11

D1. General trifluoromethylation procedure 11
D2. 1mmol scale of 2-pyridone (1a) 11

E. PURIFIED PRODUCTS 11

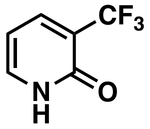

(3a) 3-(trifluoromethyl)pyridin-2(1H)-one 11

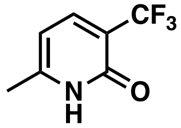

(3b) 6-methyl-3-(trifluoromethyl)pyridin-2(1H)-one 12

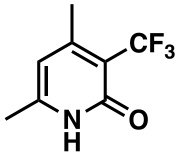

(3c) 4,6-dimethyl-3-(trifluoromethyl)pyridin-2(1H)-one 12

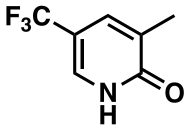

(3d) 3-methyl-5-(trifluoromethyl)pyridin-2(1H)-one 12

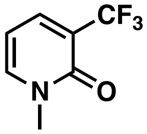

(3e) 1-methyl-3-(trifluoromethyl)pyridin-2(1H)-one 13

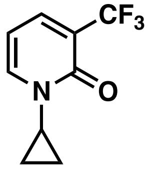

(3f) 1-cyclopropyl-3-(trifluoromethyl)pyridin-2(1H)-one 13

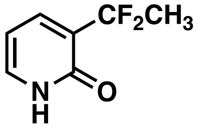

(3g) 3-(1,1-difluoroethyl)pyridine-2(1H)-one 13

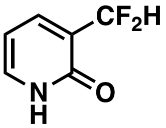

(3h) 3-(difluoromethyl)pyridin-2(1H)-one 14

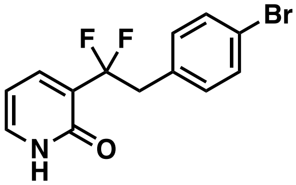

(3i) 3-(2-(4-bromophenyl)-1,1-difluoroethyl)pyridin-2(1H)-one 14

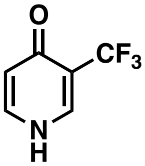

(3j) 3-(trifluoromethyl)pyridinf-4(1H)-one 14

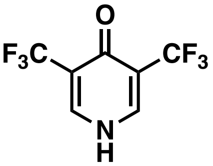

(3k) 3,5-bis(trifluoromethyl)pyridin-4-ol 15

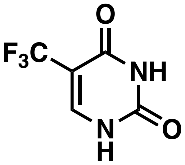

(3l) 5-(trifluoromethyl)pyrimidine-2,4(1H,3H)-dione 15

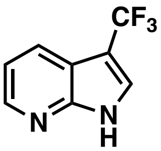

(3m-1) 3-(trifluoromethyl)-1H-pyrrolo[2,3-b]pyridine 15

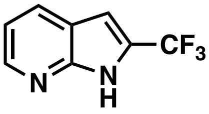

(3m-2) 2-(trifluoromethyl)-1H-pyrrolo[2,3-b]pyridine 16

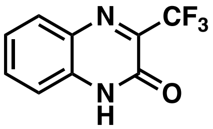

(3n) 3-(trifluoromethyl)quinoxalin-2(1H)-one 16

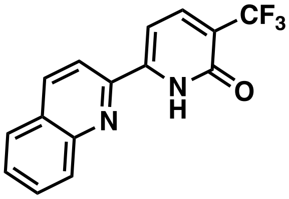

(3o) 6-(quinolin-2-yl)-3-(trifluoromethyl)pyridin-2(1H)-one 16

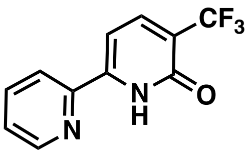

(3p) 5-(trifluoromethyl)-[2,2'-bipyridin]-6(1H)-one 17

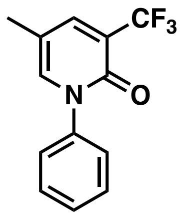

(3s) [Pirfenidone-CF_3]; 5-methyl-1-phenyl-3-(trifluoromethyl)pyridin-2(1H)-one 17

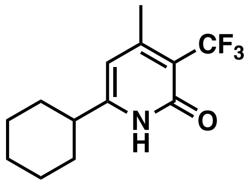

(3t) [Ciclopirox-CF_3]; 6-cyclohexyl-4-methyl-3-(trifluoromethyl)pyridin-2(1H)-one 18

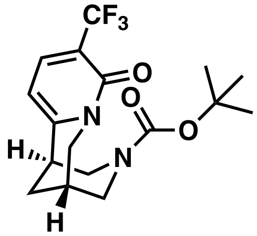

(3u) [N-Boc Cytisine-CF_3]; tert-butyl (1R,5R)-8-oxo-9-(trifluoromethyl)-1,5,6,8-tetrahydro-2H-1,5-methanopyrido[1,2-a][1,5]diazocine-3(4H)-carboxylate 18

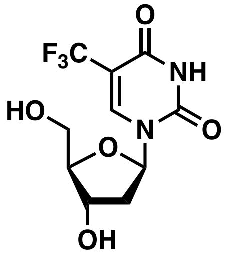

(3v) [Trifluridine]; 1-((2R,4S,5R)-4-hydroxy-5-(hydroxymethyl)tetrahydrofuran-2-yl)-5-(trifluoromethyl)pyrimidine-2,4(1H,3H)-dione 19

F. SPECTRA 20

(3a) 3-(trifluoromethyl)pyridin-2(1H)-one 21
(3b) 6-methyl-3-(trifluoromethyl)pyridin-2(1H)-one 25
(3c) 4,6-dimethyl-3-(trifluoromethyl)pyridin-2(1H)-one 29
(3d) 3-methyl-5-(trifluoromethyl)pyridin-2(1H)-one 33
(3e) 1-methyl-3-(trifluoromethyl)pyridin-2(1H)-one 37
(3f) 1-cyclopropyl-3-(trifluoromethyl)pyridin-2(1H)-one 41
(3g) 3-(1,1-difluoroethyl)pyridine-2(1H)-one 45
(3h) 3-(difluoromethyl)pyridin-2(1H)-one 49
(3i) 3-(2-(4-bromophenyl)-1,1-difluoromethyl)pyridin-2(1H)-one 53
(3j) 3-(trifluoromethyl)pyridin-4(1H)-one 57
(3k) 3,5-bis(trifluoromethyl)pyridin-4-ol 61
(3l) 5-(trifluoromethyl)pyrimidine-2,4(1H,3H)-dione 65
(3m_1) 2-(trifluoromethyl)-1H-pyrrolo[2,3-b]pyridine 68
(3m_2) 3-(trifluoromethyl)-1H-pyrrolo[2,3-b]pyridine 70
(3n) 3-(trifluoromethyl)quinoxalin-2(1H)-one 73
(3o) 6-(quinolin-2-yl)-3-(trifluoromethyl)pyridin-2(1H)-one 76
(3p) 5-(trifluoromethyl)-[2,2'-bipyridin]-6(1*H*)-one 80
(3s) [Pirfenidone-CF3]; 5-methyl-1-phenyl-3-(trifluoromethyl)pyridin-2(1H)-one 84
(3t) [Ciclopirox-CF3]; 6-cyclohexyl-4-methyl-3-(trifluoromethyl)pyridin-2(1H)-one 88

(3u) [N-Boc Cytisine-CF_3]; tert-butyl (1R,5R)-8-oxo-9-(trifluoromethyl)-1,5,6,8-tetrahydro-2H-1,5-methanopyrido[1,2-a][1,5]diazocine-3(4H)-carboxylate 92
(3v) [Trifluridine]; 96

G. LIGHT ON/OFF PROCEDURE 99

G1. Preparation of reaction mixture. 99

H. UV-VIS ANALYSIS 100

H1. General Information regarding equipment/reagents 100
H2. Preparation of solutions 100
H3. General UV-Vis procedure 100
H4. UV-Vis Spectra SI-S Figures 101

I. CYCLIC VOLTAMMETRY 106

I1. General Information regarding equipment/reagents 106
I2. Preparation of electrolyte and sample solutions 106
I3. General voltammetry procedure 107
I4. CVs / SI-S Figures 108

A. General Information

UV-Vis and Cyclic Voltammetry Information can be found in their respective sections.

A1. NMR: ^1H, ^{13}C, and ^{19}F NMR Spectra were recorded on Varian VNMRS 400 MHz, Bruker Avance AV_1 400MHz, Varian Inova 500 MHz, and Bruker Avance III HD 600MHz at room temperature. All chemical shifts were reported in parts per million (δ) and internally referenced to residual solvent proton signals unless otherwise noted. All spectral data were reported as follows: (multiplicity [singlet (s), doublet (d), doublet of doublets (dd), doublet of doublet of doublets (ddd), doublet of triplets (dt), triplet (t), triplet of triplets (tt), quartet (q), quintet (qn), and multiplet (m), heptet (h)], coupling constants [Hz], integration). Carbon and Fluorine spectra were recorded with complete decoupling.

A2. Mass Spec: Conventional mass spectra were obtained using Advion Expression^S CMS APCI/ASAP. HRMS were taken on Agilent 6530 Accurate Mass QTOF ESI.

A3. Reagents/Purification: All chemicals and reagents were purchased or synthesized from materials from Acros Organics, Cambridge Isotope Laboratories, Combi-Blocks, Fisher Scientific, Frontier Scientific, Oakwood Chemicals, Sigma Aldrich, or TCI America. *Note many of these suppliers were purchased by Thermo Scientific during Reagents were repurified and or distilled as necessary. All normal phase flash column chromatography (FCC) was performed using Grade 60 Silica Gel (230- 400 mesh) purchased from Fisher Scientific. Preparative Thin Layer Chromatography (TLC) plates contained grade 60 silica gel coated with fluorescent indicator F254 and were purchased from Fisher Scientific.

A4. Photoredox: (1) 390nm (PR-160 gen 1, 50W), (3) 390nm (PR-160L gen 1, 50W), (1) 370nm (PR-160L gen 2, 40W) LEDs, and PR-160 Fan Rig Kit were purchased from Kessil (https://kessil.com/products/science_PR160L.php). Kessil PR controller was used for light on/light off experiment. To avoid excess heating from LEDs on older set-up, fan source used: AC Infinity AXIAL 1238, 120V AC 120mm x 38mm High Speed (See Section B). Average distance between reactions and light is 6 cm. No light filters were used.

A5. Common Abbreviations Used:

A	absorbance
abs	absolute
Ac	acetyl
AcOH	acetic acid
Ac_2O	acetic anhydride
amu	atomic mass units
anhyd	anhydrous
aq	aqueous
Ar	aryl
atm	atmosphere
au	atomic units
av	average
bipy	2,2'-bipyridine or 2,2'-bipyridyl
Bn	benzyl ($PhCH_2$ also Bzl)
Boc	*tert*-butoxycarbonyl
bp	boiling point
bpy	2,2'-bipyridine or 2,2'-bipyridyl
Br	bromine
br	broad (spectral peak)
Bu	butyl
°C	degrees Celsius
calcd	calculated (for MS analysis)
cat	catalytic
CI	constitutional isomer
CIF	crystallographic information file
Cl	chlorine
cm	centimeter
cm^{-1}	wavenumbers(s)
^{13}C NMR	carbon NMR
compd	compound
concn	concentration

COSY	correlation spectroscopy (2D NMR method)
cryst	crystalline
CT	charge transfer
CV	cyclic voltammetry
δ	chemical shift (ppm) downfield from TMS
d	days; doublet (spectral)
d	density
DCE	1,2-dichloroethane
DCM	CH_2Cl_2, Dichloromethane
dil	dilute
DIPEA	*N,N*-diisopropylethylamine
DMAP	4-(*N,N*-dimethylamino)pyridine
DME	1,2-dimethoxyethane
DMF	dimethylformamide
DMSO	dimethylsulfoxide
e.g.	example (no spaces)
EPR	electron paramagnetic resonance
eq	equation
equiv.	equivalents
ESI	electrospray ionization
Et	ethyl
et al.	and others (co-authors)
etc.	and so forth
EtOAc	Ethyl Acetate
Et_3N	triethylamine (use instead of TEA)
^{19}F	fluorine NMR
FT	Fourier transform
g	gram(s); gas
G	gauss
h	hours(s)
^1H NMR	proton NMR
HOMO	highest occupied molecular orbital
HRMS	high-resolution mass spectrometry
Hz	hertz
i.e.	that is
insol	insoluble
IR	infrared
J	coupling constant in NMR
k	rate constant; Boltzmann constant
K	Kelvin
l	liquid
L	liter; ligand
lit.	literature value
λ_{max}	max UV-vis wavelength
LUMO	lowest unoccupied molecular orbital
m	meter; milli; multiplet (spectral)
M	molar (moles per liter)
M^+	parent molecular ion (in MS)
μ	micro
max	maximum
Me	methyl
MeCN	acetonitrile
MeOH	Methanol
Mes	mesityl (2,4,6-trimethylphenyl)
MHz	megahertz
min	minute(s); minimum
mL	milliliter
mm	millimeter
mM	millimolar (moles per liter)
mmol	millimole(s)
mol	mole(s)
mol wt	molecular weight
mp	melting point
MS	mass spectrometry; molecular sieves
Ms	mesyl (methanesulfonyl)
MTBE	methyl *tert*-butyl ether
M_W	weight average molecular weight
m/z	mass to charge ratio (in MS)
N	normal (equiv per liter)
n-Bu	normal butyl (primary)
NBS	*N*-bromosuccinimide
NCS	*N*-chlorosuccinimide
NMR	nuclear magnetic resonance
Nu	nucleophile
obsd	observed
PET	photoinduced electron transfer
Ph	phenyl
PMB	*p*-methoxybenzyl
ppm	part per million
Pv	pivaloyl
py	pyridine
q	quartet (spectral)
quin	quintet (spectral)
R	alkyl group
recryst	recrystallized
red	reduction
redox	reduction-oxidation
rt	room temperature (Thieme publishing r.t.)
sat.	saturated
SET	single electron transfer
S_N1	unimolecular nucleophilic substitution
S_N2	bimolecular nucleophilic substitution
sol	solid
soln	solution
t	triplet (spectral)
t	time or temp in °C
T	temperature in kelvin
t-Bu	*tert*-butyl
temp	temperature
Tf	trifluoromethanesulfonyl (triflyl)
TFA	trifluoroacetic acid
TFAA	trifluoroacetic anhydride
THF	tetrahydrofuran

TLC	thin-layer chromatography	vis	visible
Tol	toluene	vs.	versus
TS	transition state	v/v	volume to volume ratio
UV-vis	ultraviolet-visible absorption spectroscopy	wt	weight
		w/w	weight to weight rati

B. Light Set Up

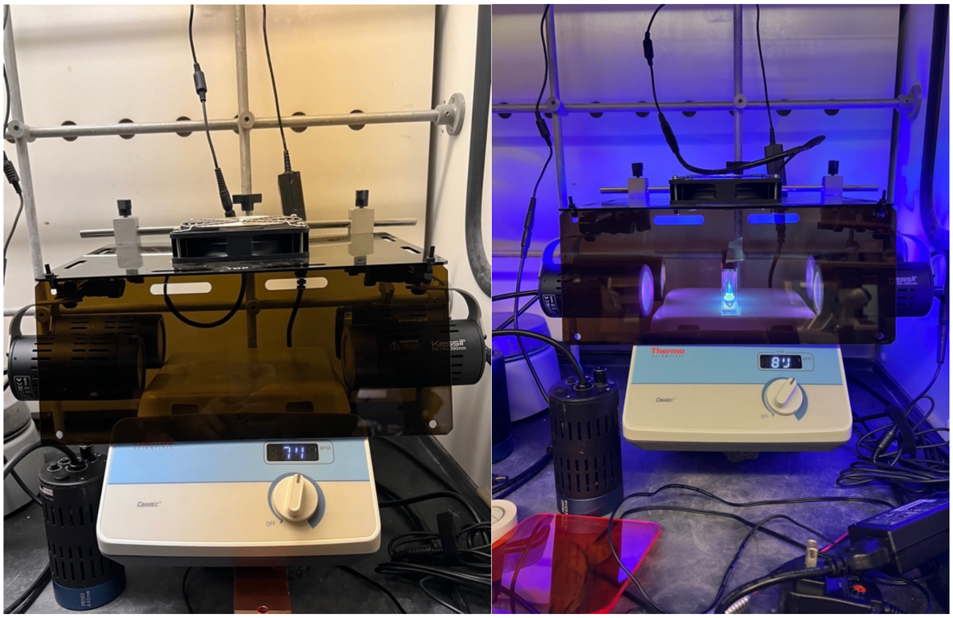

3 PR160L and 1 PR160s LED photoredox Kessil lamps (390nm) were used in the Kessil PR160 Fan Rig (Fan located on top of rig), stirring at >700 rpm. Average distance from lights to center is 6cm.

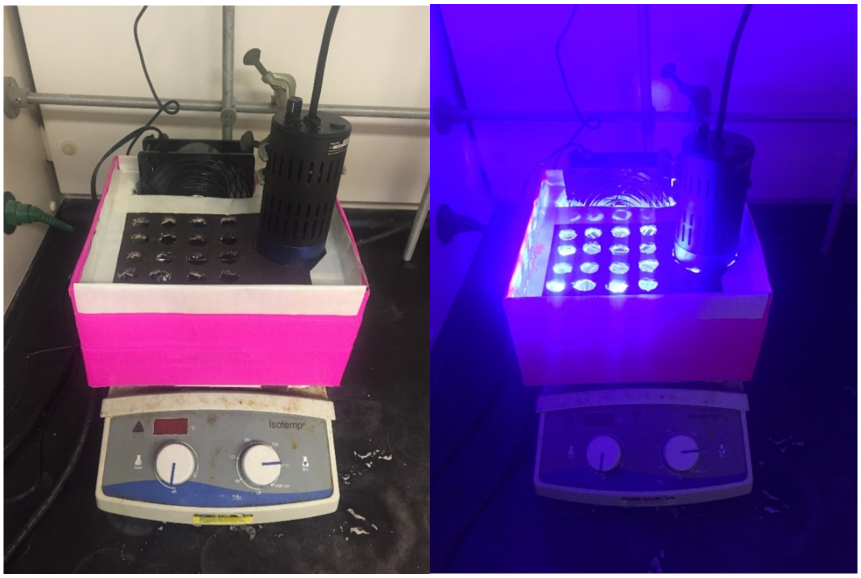

A PR160 LED photoredox Kessil lamp (370Lnm) was inserted into a cutout cardboard box with angled mirrors inside for even distribution of light. A computer fan was used to minimize heat output from the lamp. Stir rate is >700 rpm

C. Optimization

SI-Table 1

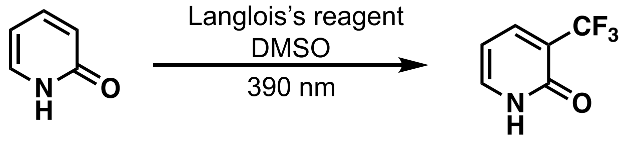

Solvent Evaluation

entry	wavelength	solvent (ratio)	conversion[a] (%)
1	390	DMSO	92
2	390	DMF	90
3	390	MeCN:H_2O (1:1)	60
4	440	MeCN:H_2O (1:1)	32
5	390	$CHCl_3$:H_2O (1:1)	25
6	390	EtOAc	28
7	390	Acetone	62
8	390	THF	ND

[a]Conversion based on NMR using 0.03125mmol TMB as a standard

SI-Table 2

Solvent Concentration Optimization

entry	Langlois' (equiv.)	concentration (M)	conversion[a] (%)
1	1.0	0.25	45
2	1.0	0.125	46
3	1.0	0.0625	51
4	1.0	0.03125	49
5	1.0	0.0178	40

[a]Conversion based on NMR using 0.3125mmol TMB as a standard

D. General Procedures

D1. General trifluoromethylation procedure

To an 8mL vial equipped with a new magnetic stir bar, 2-pyridone (11.8mg, 0.125mmol, 1.0 equiv.) was added followed by sodium trifluoromethanesulfinate+ (39.0mg, 0.25mmol, 2.0 equiv.) and DMSO (2mL, 0.0625M). For slight increased yields, reactions were purged with O_2 gas before sealed. The reaction was then capped and irradiated for 24 hours in 390nm+ light stirring at a rate >700. The reaction was quenched with DCM first, before extraction with 10% LiCl soln (30mL). Aqueous phase was then extracted 3x more with DCM. All organic layers were combined, back extracted with water, and dried with sodium sulfate before rotary evaporation at 55ºC-60 ºC for several minutes. After solvent evaporation, semi-crude material was then dried further on Schlenk line. Materials were further purified by FCC in 95:5 (DCM: MeOH), and or by prep TLC in 95:5 (DCM:MeOH) to afford trifluoromethylated product unless otherwise stated.

Note: Compounds 1 and 2 were commercially obtained by vendors such as Combi-Blocks and Sigma Aldrich and used as is.

+*varies based on substrate, prolonged reaction time and excess reagent is not detrimental, see individual compound for equivalences, purification, light used.*

D2. 1mmol scale of 2-pyridone (1a)

To an 20mL borosilicate vial equipped with a new magnetic stir bar, 2-pyridone (95.1mg, 1.0 mmol, 1.0 equiv.) was added followed by sodium trifluoromethanesulfinate+ (312.1 mg, 2.0 mmol, 2.0 equiv.) and DMSO (16mL, 0.0625M). The reaction was purged with O_2 and irradiated for 24 hours in 390nm PR-160L in the Rig Kit stirring at a rate >700. The reaction was quenched with DCM first, before extraction with 10% LiCl soln (160mL). Aqueous phase was then extracted 3x more with DCM. All organic layers were combined, back extracted with water, and dried with sodium sulfate before rotary evaporation at 55ºC - 60ºC for several minutes. After solvent evaporation, semi-crude material was then dried further on Schlenk line. Purified by FCC in 95:5 (DCM: MeOH) to afford trifluoromethylated product.

E. Purified Products

**All spectra were taken in $CDCl_3$ unless otherwise stated*

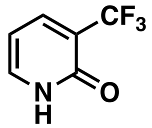

(3a) 3-(trifluoromethyl)pyridin-2(1H)-one

Conditions: See D1 general procedure, See D2 general procedure for 1 mmol scale.

Purification: 95:5 DCM:MeOH

Yield (D1): 11.88mg, 0.125mmol; isolated; 19.3mg (95% yield), 18.8mg (92% yield), 18.5mg, (90.7%), off-white solid, average is 92.5%.

Yield (D2): 95.1mg, 1mmol; 112mg, (68%) off-white solid

^{1}H NMR ($CDCl_3$, 400 MHz): δ 13.37(bs, 1H), 7.88(d, 1H, J= 7.4 Hz), 7.66 (d, 1H, J= 7.1 Hz), 6.41-6.38 (t, 1H, J= 6.6 Hz). **^{13}C {1H} NMR** ($CDCl_3$, 126 MHz): δ 161.5, 140.7 (q, J= 5 Hz), 139.1, 122.6 (q, J=272 Hz), 118.6, 105.5. **^{19}F NMR** ($CDCl_3$, 376 MHz) -65.77. This compound is also described in *J. Org. Chem.* **2020**, *85*, 3110-3124.

HRMS (ESI) m/z: $[M+H]^+$ Calcd for $C_6H_5F_3NO$ 164.0323; Found 164.0316

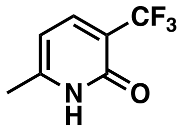

(3b) 6-methyl-3-(trifluoromethyl)pyridin-2(1H)-one

Conditions: See D1 general procedure.
Purification: 95:5 DCM:MeOH
Yield: 13.63mg, 0.125mmol; isolated: 19.8mg (89% yield), 19.5mg (88% yield), white solid
^{1}H NMR ($CDCl_3$, 400 MHz): δ 13.29 (brs, 1H), 7.73 (d, 1H, J= 7.5 Hz), 6.14 (d, 1H, J= 7.5 Hz), 2.42 (s, 3H). **^{13}C {1H} NMR** ($CDCl_3$, 126 MHz): δ 161.8, 151.1, 140.7 (q, J= 5 Hz), 122.8 (q, J= 273), 119.9, 104.8, 19.2. **^{19}F NMR** ($CDCl_3$, 376 MHz) -65.16. This compound is also described in Green. Chem. **2022**, *24*, 7388-7394
HRMS (ESI) m/z: $[M+H]^+$ Calcd for $C_7H_7F_3NO$ 178.0473; Found 178.0477

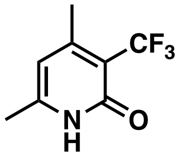

(3c) 4,6-dimethyl-3-(trifluoromethyl)pyridin-2(1H)-one

Conditions: See D1 general procedure, used 1.5 equiv. of sodium triflate.
Purification: 95:5 DCM:MeOH
Yield: 15.39mg, 0.125mmol; isolated: 19.8mg (83% yield), white solid
^{1}H NMR ($CDCl_3$, 400 MHz): δ 13.10 (bs, 1H), 5.93 (s, 1H), 2.37-2.35 (q, 3H, J= 3.15 Hz), 2.33 (s, 3H). **^{13}C {1H} NMR** ($CDCl_3$, 126 MHz): δ 162.4, 154.4, 148.5, 124.5 (q, J= 270 Hz), 114.1 (q, J= 28 Hz), 21.2 (q, J= 4 Hz), 18.7. **^{19}F NMR** ($CDCl_3$, 376 MHz) 57.29 (d)
HRMS (ESI) m/z: $[M+H]^+$ Calcd for $C_8H_9F_3NO$ 192.0636; Found 192.0637

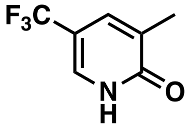

(3d) 3-methyl-5-(trifluoromethyl)pyridin-2(1H)-one

Conditions: See D1 general procedure.
Purification: 95:5 DCM:MeOH or 60:40 Hex:EtOAc
Yield: 13.64mg, 0.125mmol; isolated: 2.65mg (12% yield), 2.6mg (12% yield) white solid, Under O_2 13.64mg, 0.125mmol, isolated 4.3mg (19% yield), 4.4mg (20% yield)
^{1}H NMR ($CDCl_3$, 400 MHz): δ 11.78 (brs, 1H), 7.42 (d, 1H, J= 7.5 Hz), 6.77 (d, 1H, J= 7.5 Hz), 2.24 (s, 3H). **^{13}C {1H} NMR** ($CDCl_3$, 126 MHz): δ 163.5, 138.1, 136.1 (q, J= 30 Hz), 131.0, 120.4 (q, J= 271 Hz), 108.2 (q, J= 4 Hz), 16.4. **^{19}F NMR** ($CDCl_3$, 376 MHz) -67.17
HRMS (ESI) m/z: $[M+H]^+$ Calcd for $C_7H_7F_3NO$ 178.0473; Found 178.0470

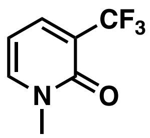

(3e) 1-methyl-3-(trifluoromethyl)pyridin-2(1H)-one

Conditions: See D1 general procedure.
Purification: 95:5 DCM:MeOH
Yield: 13.63mg, (12.07uL), 0.125mmol; isolated: 14.38mg (65% yield), white solid
^{1}H NMR ($CDCl_3$, 400 MHz): δ 7.73 (d, 1H, J= 7.15 Hz), 7.52 (d, 1H, J= 6.78 Hz), 6.24-6.20 (t, 1H, J= 6.90 Hz), 3.6 (s, 1H). **^{13}C {1H} NMR** ($CDCl_3$, 126 MHz): δ 158.8, 142.2, 138.8 (q, J= 5 Hz), 122.7 (q, J= 272 Hz), 105.9, 103.9, 37.8. **^{19}F NMR** ($CDCl_3$, 376 MHz): δ -66.09. This compound is also described in *Chem. Commun*. **2018**, *54*, 10574-10577
HRMS (ESI) m/z: $[M+H]^+$ Calcd for $C_7H_7F_3NO$ 178.0473; Found 178.0472

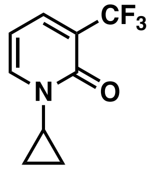

(3f) 1-cyclopropyl-3-(trifluoromethyl)pyridin-2(1H)-one

Synthesis (1f): This substrate was synthesized using the procedure found in the supplementary information of *J. Org. Chem.* **2018***. 83 (7).* 3417-3425. **^{1}H NMR (1f)** ($CDCl_3$, 400 MHz): δ 7.29 (m, 2H), 6.43 (d, 1H, J = 9.3Hz), 6.11 (t, 1H, J = 6.8Hz), 3.27 (h, 1H, J = 4Hz), 1.07 (q, 2H, J= 6.9Hz), 0.8 (q, 2H, J = 6.3Hz).
Conditions: See D1 general procedure.
Purification: 95:5 DCM: MeOH or 7:3 DCM:EtOAc
Yield: 16.89mg, 0.125mmol; isolated: 14.2 mg (56% yield), white solid
^{1}H NMR ($CDCl_3$, 500 MHz): δ 7.70 (d, 1H, J=6.3 Hz) 7.49 (d, 1H, J= 6.8 Hz), 6.19 (t, 1H, J= 6.9 Hz), 3.39-3.35 (m, 1H), 1.17 (q, 2H, J= 6.5 Hz), 0.88 (q, 2H, J=6.2 Hz). **^{13}C {1H} NMR** ($CDCl_3$, 126 MHz): δ 159.7, 140.8, 138.2 (q, J = 5 Hz), 122.8 (q, J= 271 Hz), 119.5, 103.5, 32.6, 6.8. **^{19}F NMR** ($CDCl_3$, 376 MHz): δ -65.88
HRMS (ESI) m/z: $[M+H]^+$ Calcd for $C_9H_9F_3NO$ 204.0635; Found 204.0646

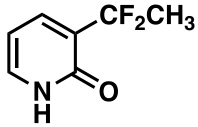

(3g) 3-(1,1-difluoroethyl)pyridine-2(1H)-one

Conditions: See D1 general procedure.
Purification: 95:5 DCM:MeOH
Yield: 11.88mg, 0.125mmol; isolated: 18.0mg (90% yield), white powder
^{1}H NMR ($CDCl_3$, 400 MHz): δ 13.18 (brs, 1H), 7.77 (d, 1H, J= 6.9 Hz), 7.50 (d, 1H, J= 8.3 Hz), 6.35 (t, 1H, J= 6.8 Hz), 2.07 (t, 3H, J= 19.2 Hz). **^{13}C {1H} NMR** ($CDCl_3$, 126 MHz): δ 162.5 (t, J= 4 Hz), 138.2 (t, J= 8 Hz), 136.7, 127.2 (t, J= 26 Hz), 120.3 (t, J= 234 Hz), 105.9, 23.2 (t, J= 27 Hz). **^{19}F NMR** ($CDCl_3$, 376 MHz): δ -86.29 (q)

HRMS (ESI) m/z: $[M+H]^+$ Calcd for $C_7H_8F_2NO$ 160.0574; Found 160.0573

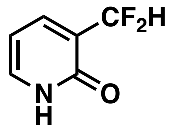

(3h) 3-(difluoromethyl)pyridin-2(1H)-one

Conditions: See D1 general procedure. Used 2.0 equivalents of sodium difluorosulfinate.
Purification: 95:5 DCM:MeOH
Yield: 11.88mg, 0.125mmol; 1.6mg (9% yield), Under O_2 isolated: 2.7mg (15% yield), off-white crystalline solid
^{1}H NMR ($CDCl_3$, 500 MHz): δ 13.01 (brs, 1H), 7.82 (d, 1H, J= 6.5 Hz), 7.52 (d, 1H, J= 5.2 Hz), 6.81 (t, 1H, J= 53.8 Hz), 6.41 (t, 1H, J= 6.1 Hz). **^{13}C {1H} NMR** ($CDCl_3$, 126 MHz): δ 162.9, 139.3 (t, J= 6 Hz), 137.1, 124.7 (t, J= 22 Hz), 110.9 (t, J= 238 Hz), 106.5. **^{19}F NMR** ($CDCl_3$, 376 MHz): δ -118.88 (d, J= 55.1 Hz)
HRMS (ESI) m/z: $[M+H]^+$ Calcd for $C_6H_6F_2NO$ 146.0417; Found 146.0407

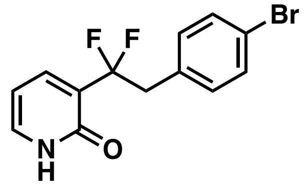

(3i) 3-(2-(4-bromophenyl)-1,1-difluoroethyl)pyridin-2(1H)-one

Conditions: See D1 general procedure
Purification: 95:5 DCM:MeOH
Yield: 11.88mg, 0.125mmol; isolated: 31.0mg (79% yield), white crystalline solid/powder, *degrades in solution (regular and deuterated) over prolonged periods of time*
^{1}H NMR ($CDCl_3$, 400 MHz): δ 13.03 (brs, 1H), 7.54 (d, 1H, J= 5.9 Hz), 7.49 (d, 1H, J=7.9 Hz), 7.37 (d, 2H, J= 8.4 Hz), 7.10 (d, 2H, J= 7.4), 6.27 (t, 1H, J= 6.7), 3.75 (t, 2H, J= 15.7 Hz). **^{13}C {1H} NMR** ($CDCl_3$, 126 MHz): δ 162.3, 139.6 (t, J= 8 Hz), 136.7, 132.1, 131.4, 122.8, 121.4, 120.3, 106.1, 41 (t, J= 26 Hz), 29.7. **^{19}F NMR** ($CDCl_3$, 376 MHz): δ -96.30 (t)
HRMS (ESI) m/z: $[M+H]^+$ Calcd for $C_{13}H_{11}BrF_2NO$ 313.9992; Found: 314.0013, 316.0023 (^{79}Br, ^{81}Br)

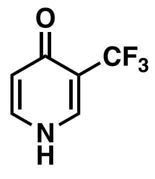

(3j) 3-(trifluoromethyl)pyridinf-4(1H)-one

Conditions: See D1 general procedure, used 1.3 equivalents of sodium triflate.
Purification: 90:10 DCM:MeOH
Yield: 11.89mg**,** 0.125mmol; isolated 10.5mg (51% yield), white crystalline solid

^{1}H NMR ($CO(CD_3)_2$, 500 MHz): δ 10.87 (brs, 1H), 8.11 (s, 1H), 7.76 (d, 1H, J=7.3 Hz), 6.27 (d, 1H, J= 7.5 Hz). **^{13}C {1H} NMR** (CD_3OD, 126 MHz): δ 177.9, 141.0, 138.8 (q, J= 5 Hz), 124.9 (q, J= 272 Hz), 120.8, 119.6 (q, J= 28 Hz). **^{19}F NMR** ($CO(CD_3)_2$, 376 MHz): δ -65.62
HRMS (ESI) m/z: $[M+H]^+$ Calcd for $C_6H_5F_3NO$ 164.0323; Found: 164.0315

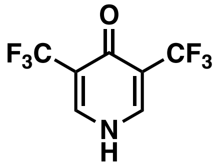

(3k) 3,5-bis(trifluoromethyl)pyridin-4-ol

Conditions: See D1 general procedure, used 3.0 equivalents of sodium triflate
Purification: 90:10 DCM:MeOH
Yield: 11.88mg, 0.125mmol; isolated: 6.5mg (23% yield), white solid
^{1}H NMR (CD_3OD, 500 MHz): δ 8.24 (s, 2H). **^{13}C {1H} NMR** (CD_3OD, 126 MHz): δ 210.1, 140.6, 123.1 (q, J= 273), 30.6. **^{19}F NMR** (CD_3OD, 376 MHz): δ -66.51
HRMS (ESI) m/z: $[M+H]^+$ Calcd for $C_7H_4F_6NO$, 232.0197; Found 232.0200

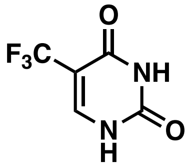

(3l) 5-(trifluoromethyl)pyrimidine-2,4(1H,3H)-dione

Conditions: See D1 general procedure
Purification: Purification via recrystallization using residual DMSO and dilute with DCM.
Yield: 14.011mg, 0.125mmol; isolated: 13.5mg (60% yield), white solid
^{1}H NMR (CD_3OD, 500 MHz): δ 7.93 (s, 1H). **^{13}C {1H} NMR** (CD_3OD, 126 MHz): δ 162.0, 152.4, 144.7 (q, J= 6 Hz), 126.0 (q, J= 265 Hz), 101.8. **^{19}F NMR** (CD_3OD, 376 MHz): δ -64.85. This compound is also described in *Tetrahedron.* **1982**, *23*, 4099-4100 and *Chem. Comm*. **2018**, *54*, 13662-13665
HRMS (ESI) m/z: $[M-H]^+$ Calcd for $C_5H_2F_3N_2O_2$ 179.0077; Found 179.0081

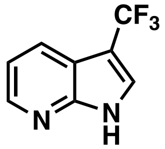

(3m-1) 3-(trifluoromethyl)-1H-pyrrolo[2,3-b]pyridine

Conditions: See D1 general procedure, used 0.35mmol scale.
Purification: 80:20 Hexanes:EtOAc
Yield: 41.35mg, 0.35mmol; isolated: 23.4mg (35.9% yield), white powder, 70% overall of both regioisomers, (1.1 : 1 C3 favored)
^{1}H NMR ($CDCl_3$, 400 MHz): δ 13.76 (brs, 1H), 8.50 (d, 1H, J= 4.8 Hz), 8.10 (d, 1H, J= 8.0 Hz), 7.23 (dd, 1H, J= 7.8, 4.7 Hz), 6.91 (d, 1H, J= 1.31)

^{13}C {1H} NMR ($CDCl_3$, 126 MHz): δ 148.5, 144.5, 131.4, 127.4 (q, J= 39 Hz), 121.3 (q, J= 271 Hz), 119.8, 116.9, 101.3 (q, J= 5 Hz). **^{19}F NMR** ($CDCl_3$, 376 MHz): δ -61.22 (s). This compound is also described in *J. Am. Chem. Soc.* **2019**, *141*, 12872-12879
HRMS (ESI) m/z: $[M+H]^+$ Calcd for $C_8H_5F_3N_2$ 187.0483; Found 187.0468

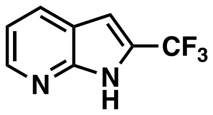

(3m-2) 2-(trifluoromethyl)-1H-pyrrolo[2,3-b]pyridine

Conditions: See D1 general procedure, used 0.35mmol scale.
Purification: 70:30 Hexanes:EtOAc
Yield: 41.35mg, 0.35mmol; isolated: 22.1mg (33.9% yield), white powder, 70% overall of both regioisomers
^1H NMR ($CDCl_3$, 500 MHz): δ 11.77 (brs, 1H), 8.45 (dd, 1H, J= 3.16, 1.41 Hz), 8.13 (d, 1H, J= 7.97 Hz), 7.76 (q, 1H, J= 7.97 Hz), 7.25 (q, 1H, J= 4.78 Hz)
^{13}C {1H} NMR ($CDCl_3$, 126 MHz): δ 148.3, 143.8, 128.7, 125.3 (q, J= 4 Hz), 123.7 (q, J= 271 Hz), 117.3, 116.8 ,105.9 (q, J = 38 Hz). **^{19}F NMR** ($CDCl_3$, 376 MHz): δ -57.36 (s). This compound is also described in *J. Am. Chem. Soc.* **2019**, *141*, 12872-12879
HRMS (ESI) m/z: $[M+H]^+$ Calcd for $C_8H_5F_3N_2$ 187.0483; Found 187.0468

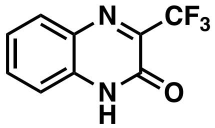

(3n) 3-(trifluoromethyl)quinoxalin-2(1H)-one

Conditions: See D1 general procedure.
Purification: 95:5 DCM: MeOH
Yield: 18.25mg, 0.125mmol; 17.3mg (65% yield), white solid
^1H NMR ($CDCl_3$, 400 MHz): δ 10.26 (brs, 1H), 8.42 (d, 1H, J= 8.2), 8.13 (d, 1H, J= 7.6 Hz), 8.07 (s, 1H), 7.60 (t, 1H, J= 7.9 Hz). **^{13}C {1H} NMR** ($CDCl_3$, 126 MHz): δ 160.9, 147.5, 146.6, 133.1 (q, J= 6 Hz), 131.6, 127.2, 126.0, 125.5, 123.8. **^{19}F NMR** ($CDCl_3$, 376 MHz): δ -60.59. This compound is also described in *Org. Chem. Front.* **2019**, *6*, 2392-2397
HRMS (ESI) m/z: $[M+H]^+$ Calcd for $C_9H_6F_3N_2O$ $[M+H]^+$, 215.0432; Found 215.0448

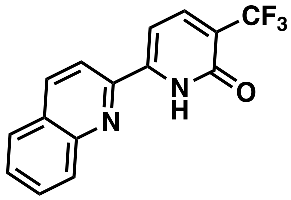

(3o) 6-(quinolin-2-yl)-3-(trifluoromethyl)pyridin-2(1H)-one

Ligand Synthesis: This substrate was synthesized using the procedure found in the supplementary information of *Science.* **2021**, *374*, 1281-1285. **^1H NMR (1o)** ($CDCl_3$, 400 MHz) δ10.97 (brs, 1H), 8.26 (d, 1H, J = 8.6 Hz), 8.09 (d, 1H, J = 8.6Hz), 7.86 (t, 2H, J = 7.9Hz), 7.78 (t,

1H, J = 6.9Hz), 7.60 (t, 1H, J = 6.9Hz), 7.53 (t, 1H, J = 6.3Hz), 6.95 (d, 1H, J = 6.7Hz), 6.70 (d, 1H, J = 9.0Hz)
Conditions: See D1 general procedure.
Purification: 95:5 DCM: MeOH
Yield: 27.78mg, 0.125mmol; 17.6mg (48.6% yield), pale white solid
^{1}H NMR ($CDCl_3$, 400 MHz): δ 11.16 (brs, 1H), 8.34 (d, 1H, J=9.2 Hz), 8.16 (d, 1H, J= 8.3 Hz), 7.94-7.88 (m, 3H), 7.83 (t, 1H, J=7.5 Hz), 7.66 (t, 1H, J= 7.5 Hz), 6.99 (d, 1H, J=7.5 Hz)
^{13}C {1H} NMR ($CDCl_3$, 126 MHz): δ 158.4, 147.1, 145.8, 145.3, 139.9 (q, J= 5 Hz), 138.1, 129.8, 128.5, 127.6, 124.0, 121.3, 116.8, 29.7. **^{19}F NMR** ($CDCl_3$, 376 MHz): δ -65.55
HRMS (ESI) m/z: $[M+H]^+$ Calcd for $C_{15}H_{10}F_3N_2O$ 291.0745; Found 291.0759

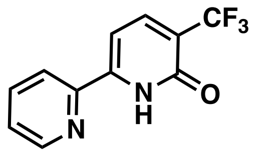

(3p) 5-(trifluoromethyl)-[2,2'-bipyridin]-6(1H)-one

Ligand Synthesis: This substrate was synthesized using the procedure found in the supplementary information of *Science.* **2021**, *374*, 1281-1285. **^{1}H NMR (1p)** ($CDCl_3$, 400 MHz) δ10.81 (brs, 1H), 8.64 (d, 1H, J = 4.6 Hz), 7.81 (m, 2H), 7.48 (m, 1H), 7.35 (m, 1H), 6.8 (d, 1H, J = 7.1Hz), 6.63 (d, 1H, J = 9.5Hz)
Conditions: See D1 general procedure.
Purification: 95:5 DCM : MeOH
Yield: 21.5mg (0.125mmol); 12.5mg (41.6% yield), pale yellow solid
^{1}H NMR (CD_2Cl_2, 400 MHz): δ 10.86 (brs, 1H), 8.70 (d, 1H, J= 4.2 Hz), 7.94-7.87 (m, 3H), 7.45 (t, 1H, J= 5.1 Hz), 6.86 (d, 1H, J= 7.4 Hz). **^{13}C {1H} NMR** (CD_3OD, 126 MHz): δ 154.1, 145.4, 142.5, 141.7, 136.1 (q, J= 5 Hz), 133.6, 122.3, 121.6, 118.7 (q, J= 270 Hz), 116.5, 96.6. **^{19}F NMR** (CD_2Cl_2, 376 MHz): δ -65.82
HRMS (ESI) m/z: $[M+Na]^+$ Calcd for $C_{11}H_7F_3N_2ONa$ 263.0408; Found 263.0425

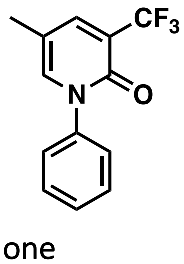

(3s) [Pirfenidone-CF_3]; 5-methyl-1-phenyl-3-(trifluoromethyl)pyridin-2(1H)-one

Conditions: See D1 general procedure.
Purification: 95:5 DCM: MeOH
Yield: 23.15mg ,0.125mmol; 14.3mg (45% yield), clear oil/white powder
^{1}H NMR ($CDCl_3$, 400 MHz): δ 7.69 (s, 1H), 7.50-7.47 (m, 2H), 7.44-7.43 (m, 1H), 7.38-7.34 (m, 3H), 2.16 (s, 3H). **^{13}C {1H} NMR** ($CDCl_3$, 126 MHz): δ 157.5, 141.7 (q, J= 5 Hz), 139.9, 139.3,

129.3, 128.8, 126.5, 123.7, 121.5, 113.1, 16.9. **^{19}F NMR** ($CDCl_3$, 376 MHz): δ -65.90. This compound is also described in *Chem. Commun*. **2018**, *54*, 10574-10577
HRMS (ESI) m/z: $[M+H]^+$ Calcd for $C_{13}H_{11}F_3NO$ 254.0792; Found 254.0794

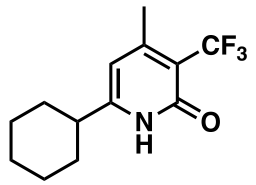

(3t) [Ciclopirox-CF_3]; 6-cyclohexyl-4-methyl-3-(trifluoromethyl)pyridin-2(1H)-one
Conditions: See D1 general procedure.
Purification: 97:3 DCM: MeOH // 6:4 Hexanes EtOAc
Yield: 25.9mg of *N*-OH SM**,** 0.125mmol; 11.34mg (35% yield of dehydroxylated product), 15.0 mg (46% yield oxygen sparged), 15.1 mg (46% yield oxygen sparged yellow-orange solid
^{1}H NMR ($CDCl_3$, 400 MHz): δ 12.48 (brs, 1H), 5.90 (s, 1H), 2.51-2.45 (t, 1H, J= 12.0 Hz), 2.38-2.36 (q, 3H, J= 3.3 Hz), 1.91-1.84 (t, 4H, J= 13.78 Hz), 1.74 (d, 1H, J= 10 Hz), 1.53-1.28 (m, 5H). **^{13}C {1H} NMR** ($CDCl_3$, 126 MHz): δ 162.2, 156.9, 124.4 (q, J= 274 Hz), 114.3 (q, J= 28 Hz), 107.29, 42.3, 31.3, 26.0, 25.3, 21.2 (q, J= 4 Hz). **^{19}F NMR** ($CDCl_3$, 376 MHz): δ -57.35
HRMS (ESI) m/z: $[M+H]^+$ Calcd for $C_{13}H_{17}F_3NO$ 260.1262; Found: 260.1260

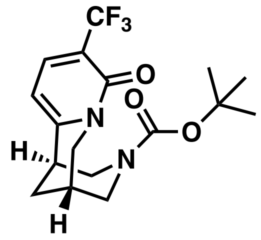

(3u) [N-Boc Cytisine-CF_3]; tert-butyl (1R,5R)-8-oxo-9-(trifluoromethyl)-1,5,6,8-tetrahydro-2H-1,5-methanopyrido[1,2-a][1,5]diazocine-3(4H)-carboxylate
Synthesis: Cytisine was purchased through Combi-Blocks, and subsequently boc-protected using 1.2 equiv. of Boc_2O, 1.5 equiv. of Et_3N in 0.2M of DCM. **^{1}H NMR (1u)** ($CDCl_3$, 400 MHz) δ7.26 (m, 1H), 6.42 (d, 1H, J = 9.0Hz), 6.05 (m, 1H), 4.17 (m, 2H), 3.81 (dd, 1H, J = 15.5, 6.6Hz), 3.05 (m, 3H), 2.4 (m, 1H), 1.96 (q, 2H, J =13.3Hz), 1.32 (m, 9H).
Conditions: See D1 general procedure.
Purification: 95:5 DCM: MeOH
Yield: 36.29mg, 0.125mmol; 14.78mg (33% yield) white solid
^{1}H NMR ($CDCl_3$, 400 MHz): δ 7.65 (d, J= 7.9 Hz, 1H), 6.12 (d, J= 5.7 Hz, 1H), 4.36-4.13 (m, 3H), 3.85-3.81 (m, 1H), 3.06-2.97 (m, 3H), 2.44 (brs, 1H), 1.98 (brs, 2H), 1.38-1.21 (m, 9H). **^{13}C {1H} NMR** ($CDCl_3$, 126 MHz): δ 159.1, 154.3, 137.6, 123.1 (q, J= 271 Hz), 116.7 (q, J= 29 Hz), 103.9, 80.8, 50.4, 49.1, 35.3, 29.6, 28.0, 27.4, 25.9. **^{19}F NMR** ($CDCl_3$, 376 MHz): δ -65.77
HRMS (ESI) m/z: $[M+Na]^+$ Calcd for $C_{17}H_{21}F_3N_2O_3Na$ 381.1410; Found: 381.1425

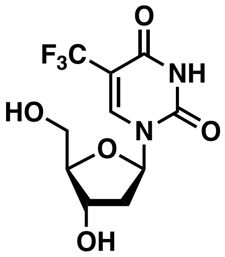

(3v) [Trifluridine]; 1-((2R,4S,5R)-4-hydroxy-5-(hydroxymethyl)tetrahydrofuran-2-yl)-5-(trifluoromethyl)pyrimidine-2,4(1H,3H)-dione

Conditions: See D1 general procedure.

Purification: 88:12 DCM: MeOH

Yield: 28.52mg, 0.125mmol; 20.8mg (56% yield) white solid

^{1}H NMR (CD_3OD, 500 MHz): δ 8.78 (s, 1H), 6.24 (t, J= 6.15 Hz,1H), 4.43-4.40 (m, 1H), 3.96 (q, J= 3.06 Hz, 1H), 3.79 (dd, J= 2.84, 3.02 Hz, 2H), 2.65 (s, 1H), 2.39-2.34 (m, 1H), 2.30-2.25 (m, 1H), 2.16 (s, 1H). **^{13}C {1H} NMR** (CD_3OD, 126 MHz): δ 161.2, 151.3, 143.7, 123.9 (q, J=269), 105.4 (q, J= 32.77), 89.4, 87.6, 71.7, 62.2, 42.1. **^{19}F NMR** (CD_3OD, 376 MHz): δ -64.52. This compound is also described in *Proc. Natl. Acad. Sci. USA*. **2011**, *35*, 14411-14415.

HRMS (ESI) m/z: $[M+Na]^+$ Calcd for $C_{10}H_{11}F_3N_2O_5Na$ 319.0517; Found: 319.0537

F. Spectra

2-Pyridone H-Bonding with DMSO solvent in $CDCl_3$; 2-Pyridone starting material *in black*, 2-pyridone mixed with DMSO taken in $CDCl_3$ is *in faint purple*.

^{1}H NMR of **3a** and **3a** with DMSO (400 MHz, $CDCl_3$)

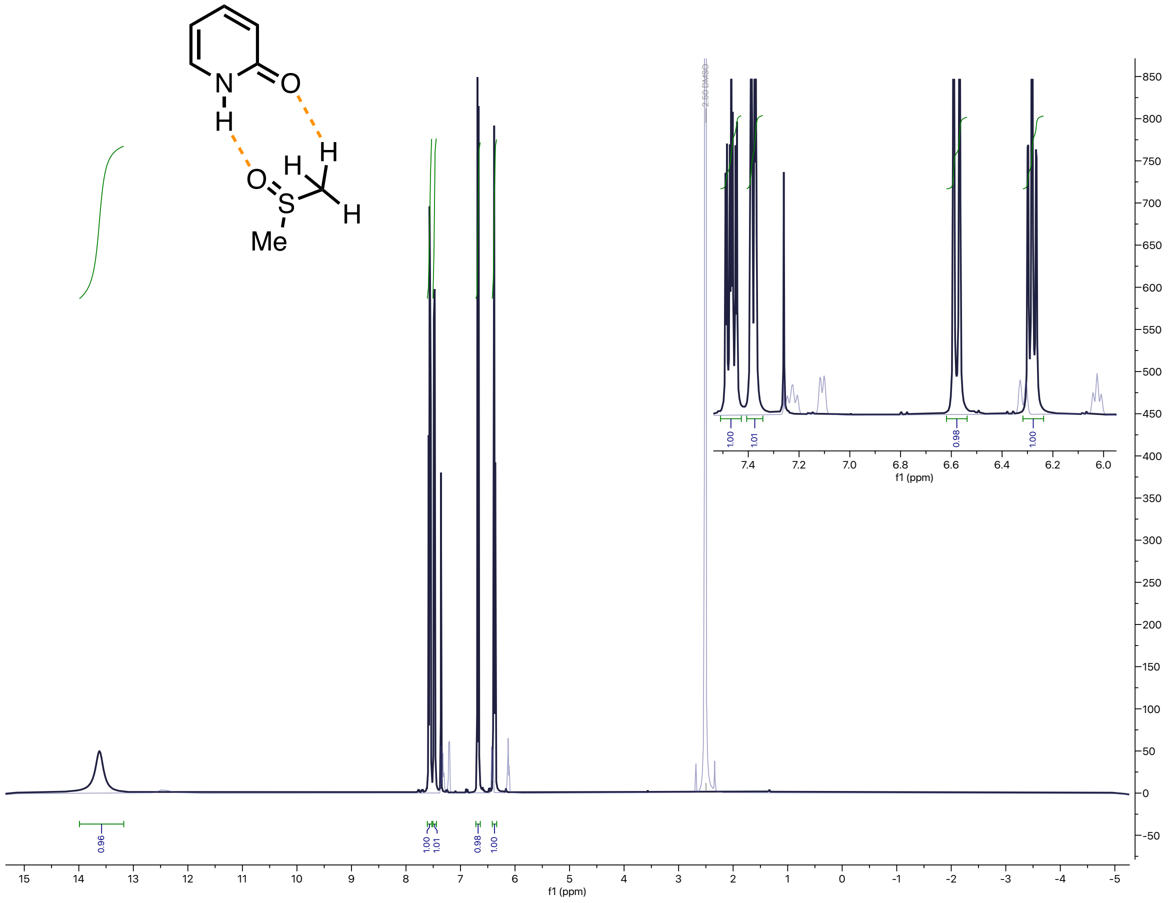

(3a) 3-(trifluoromethyl)pyridin-2(1H)-one

This compound is also described in *J. Org. Chem.* **2020**, *85*, 3110-3124.

3a - ^{1}H NMR (400 MHz, $CDCl_3$)

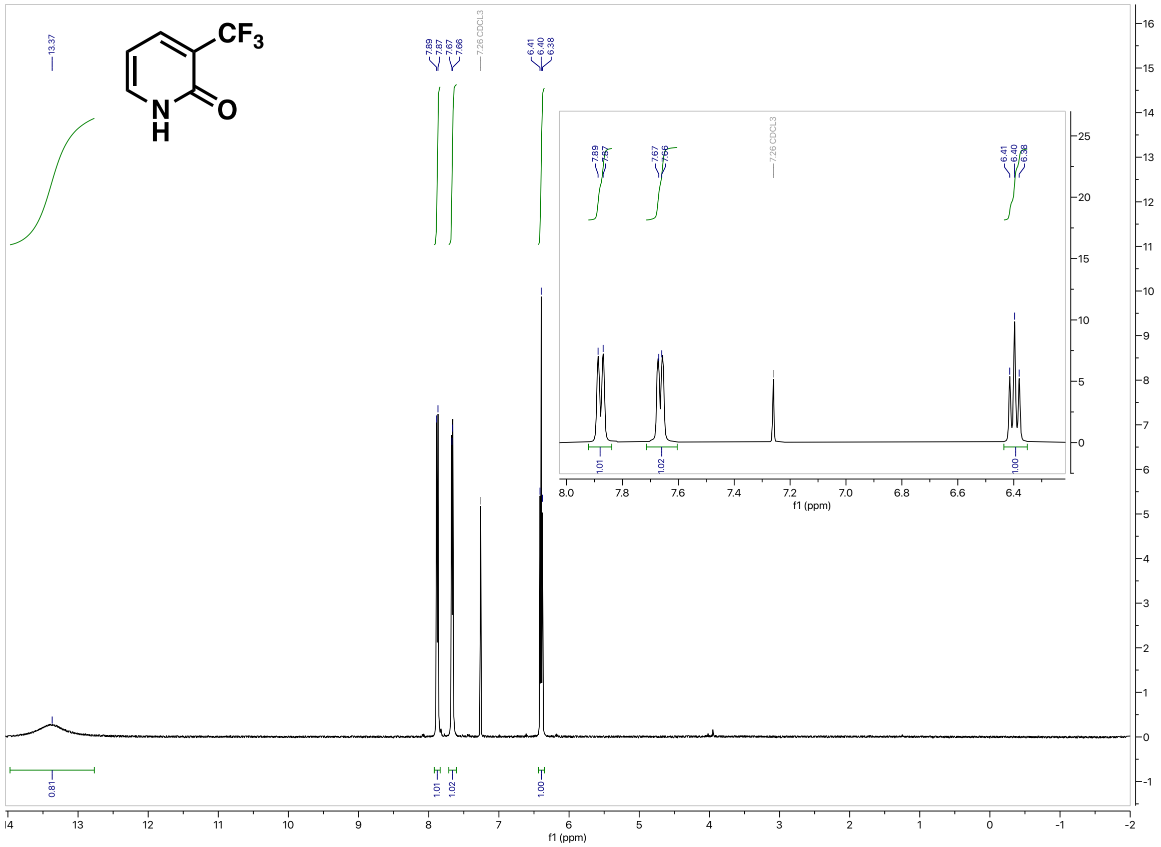

3a - ^{19}F NMR (376 MHz, $CDCl_3$)

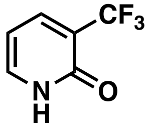

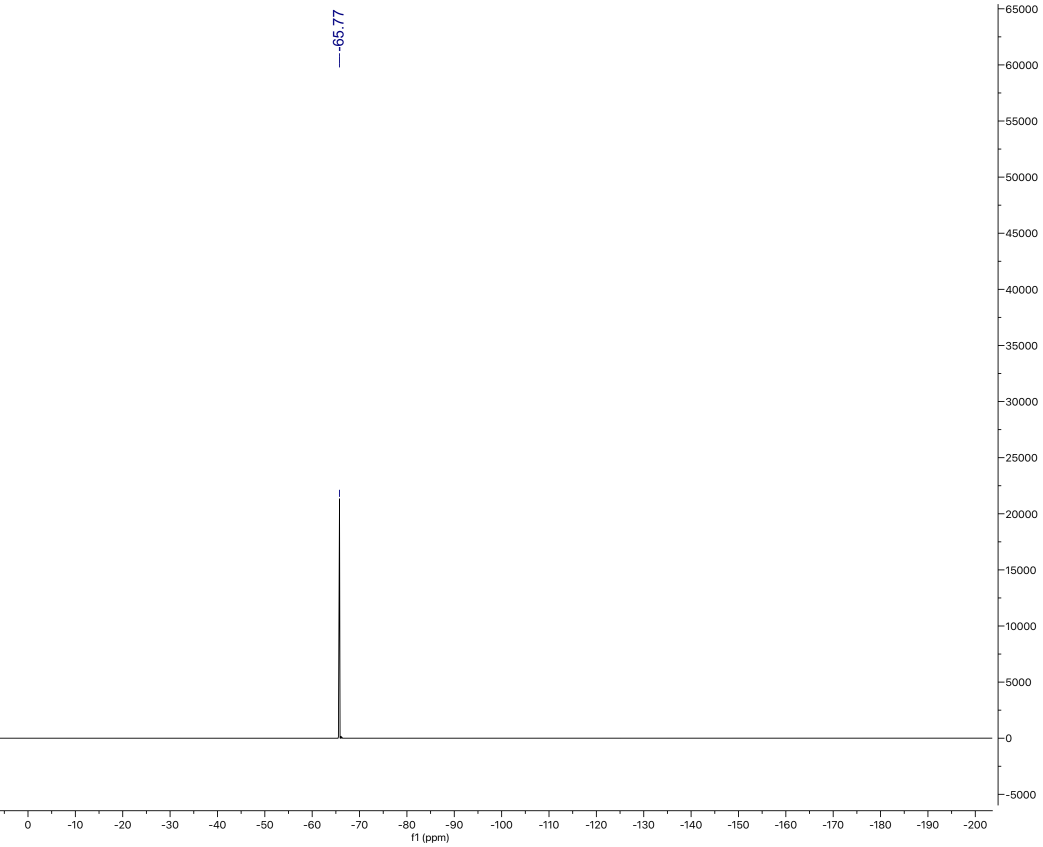

3a - ^{13}C NMR (126 MHz, $CDCl_3$)

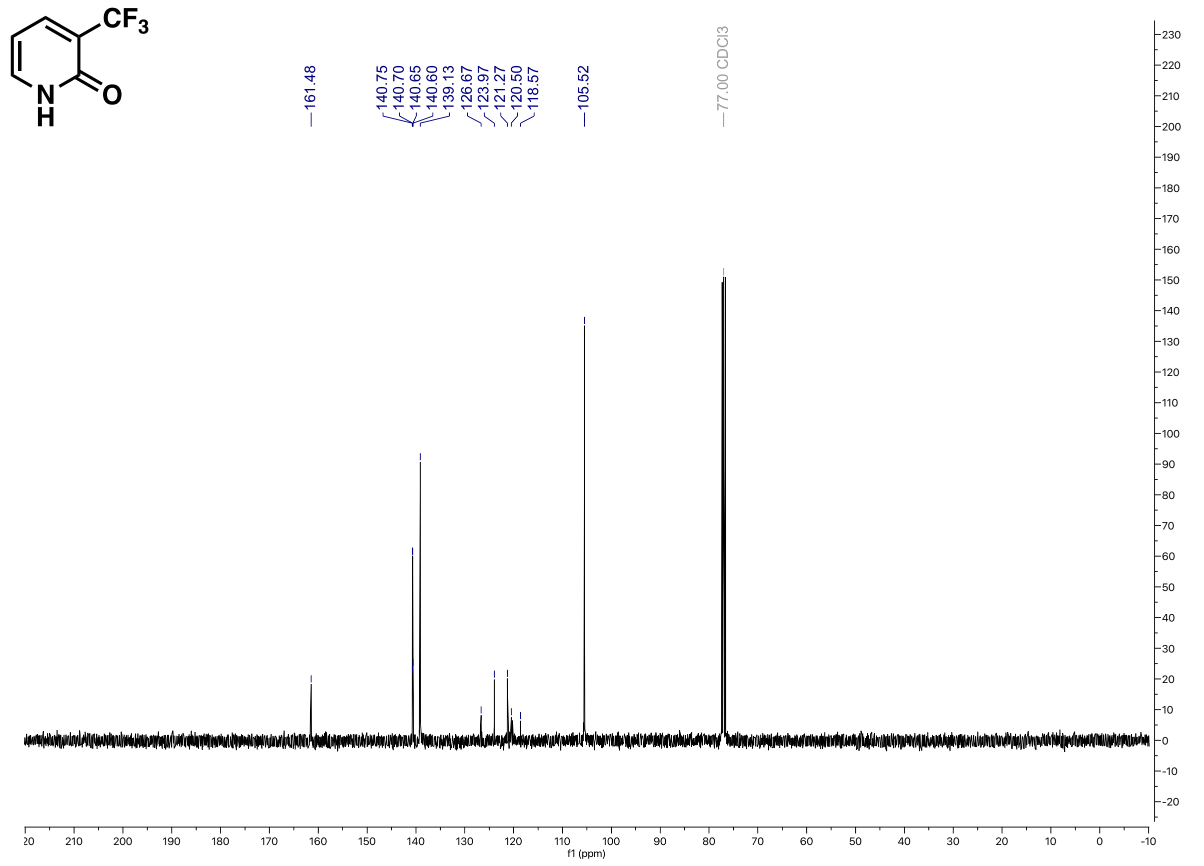

3a – HRMS (ESI)

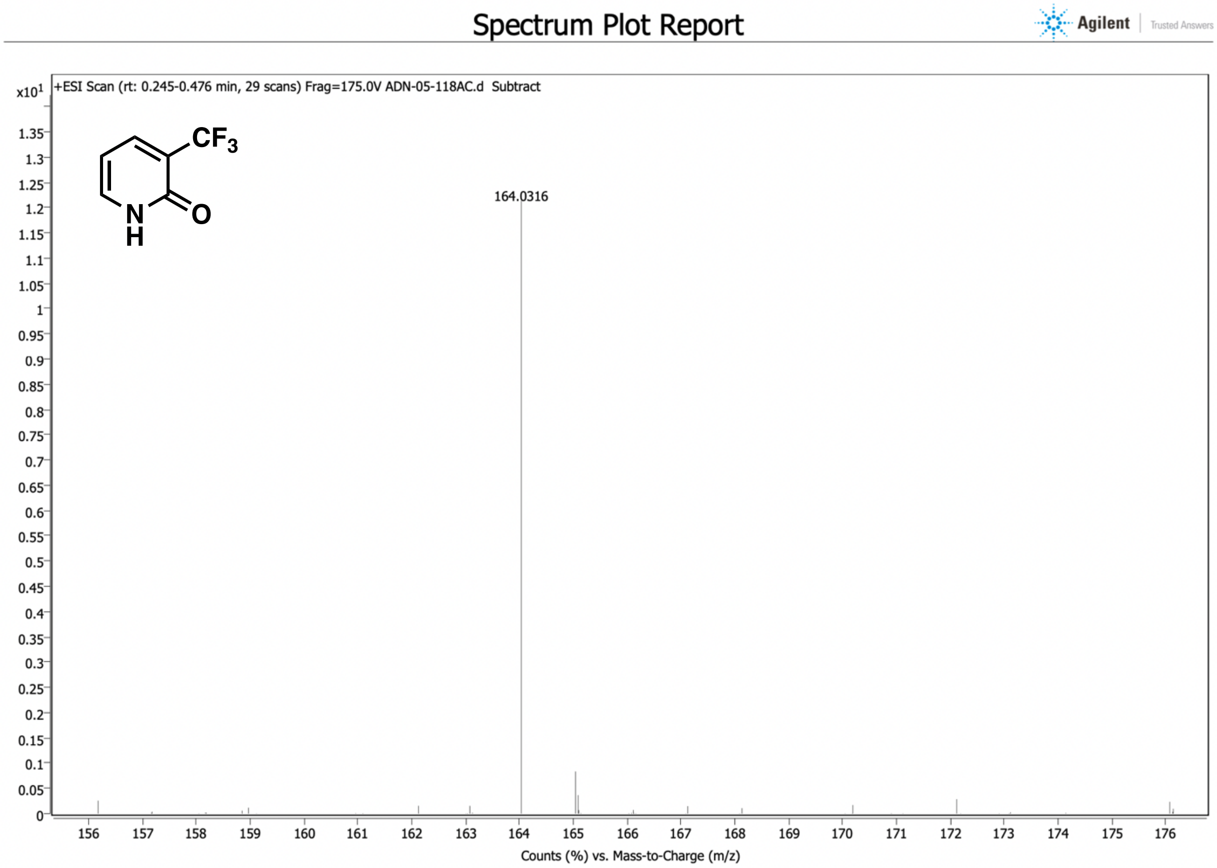

Spectrum Plot Report
Agilent
Trusted Answers
x10^1
+ESI Scan (rt: 0.245-0.476 min, 29 scans) Frag=175.0V ADN-05-118AC.d Subtract
CF3
N
H
O
164.0316
Counts (%) vs. Mass-to-Charge (m/z)

(3b) 6-methyl-3-(trifluoromethyl)pyridin-2(1H)-one

This compound is also described in Green. Chem. **2022**, *24*, 7388-7394

3b - ^{1}H NMR (400 MHz, $CDCl_3$)

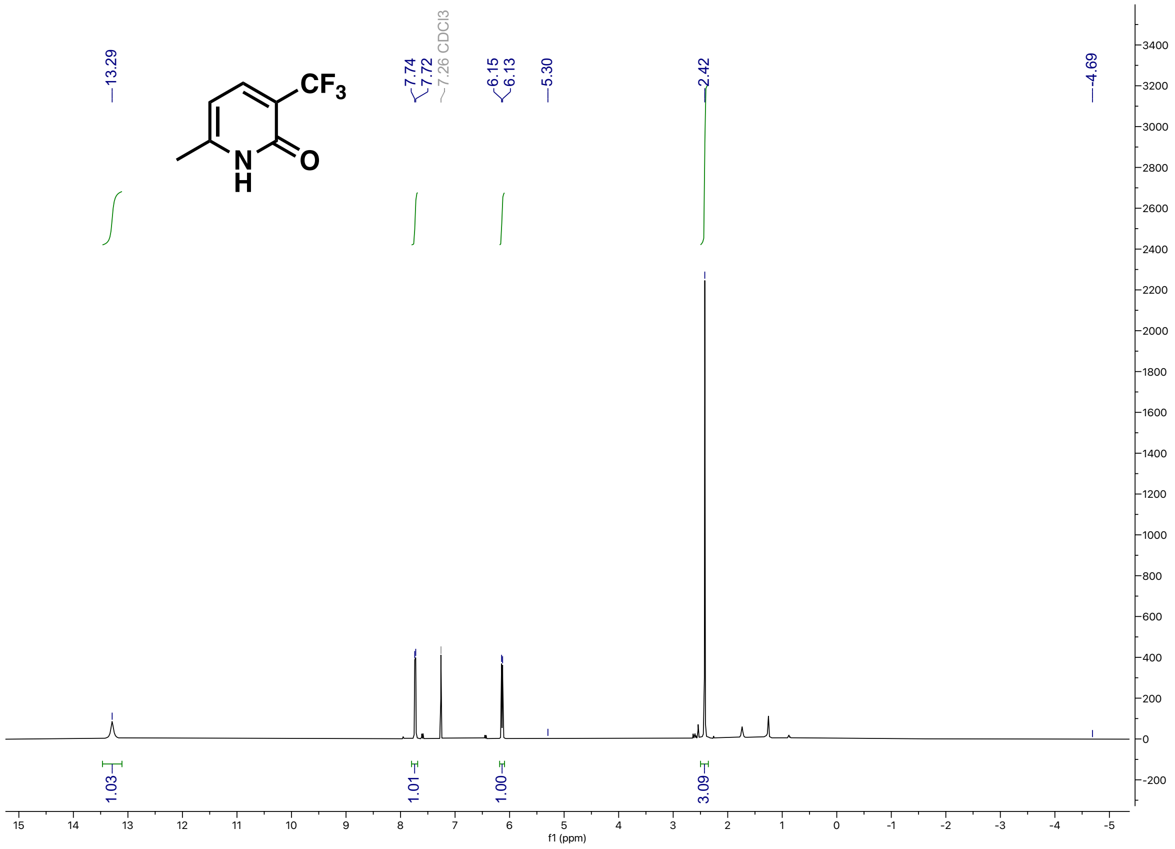

3b - ^{19}F NMR (376 MHz, $CDCl_3$)

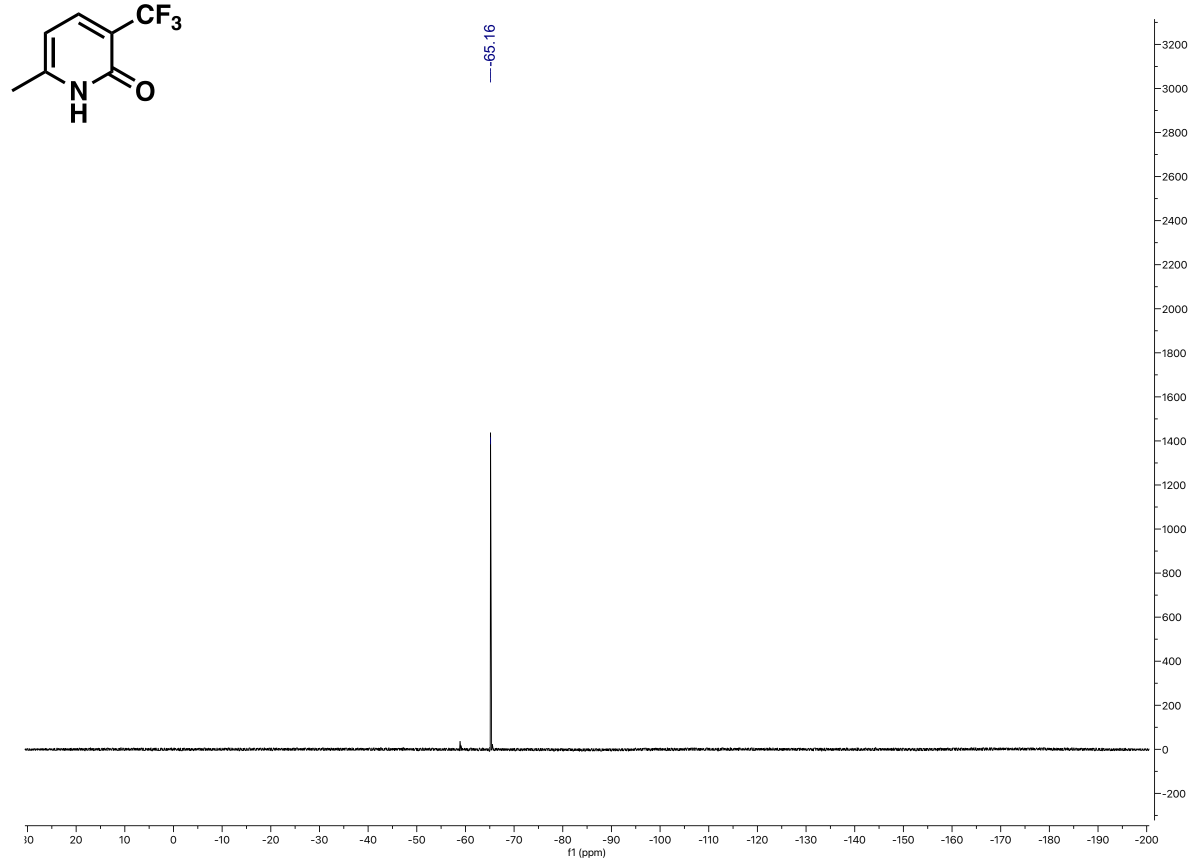

3b - ^{13}C NMR (126 MHz, $CDCl_3$)

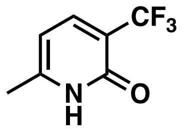

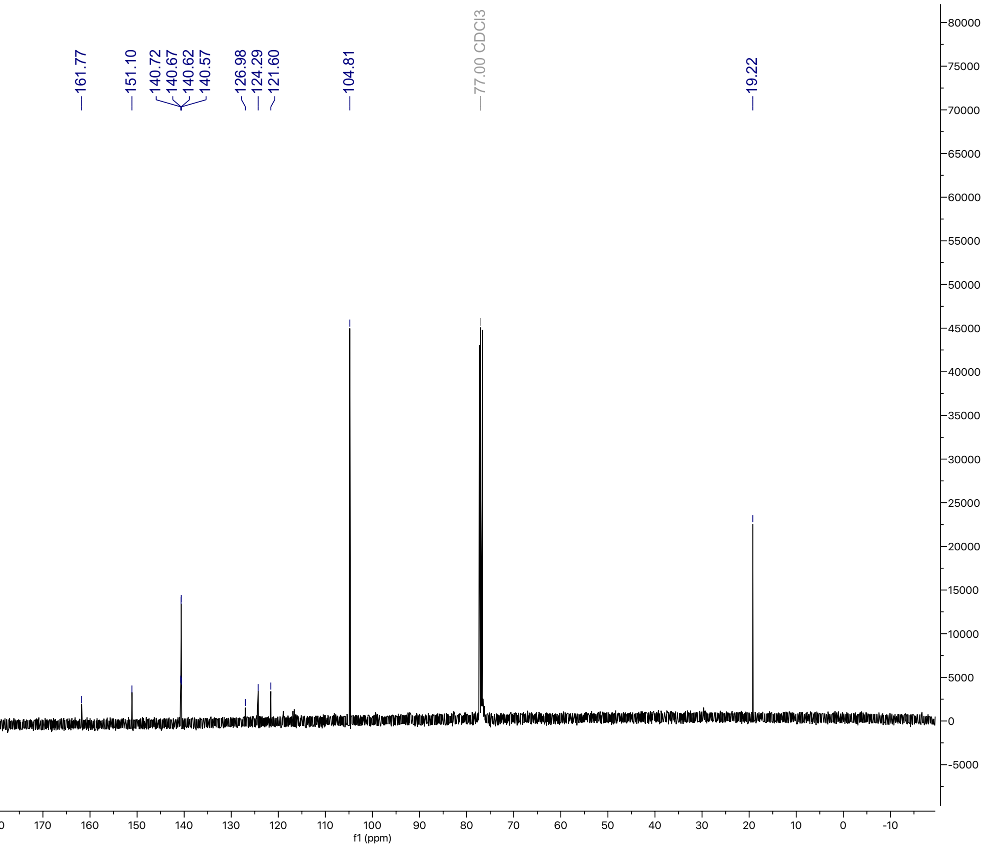

3b - HRMS (ESI)

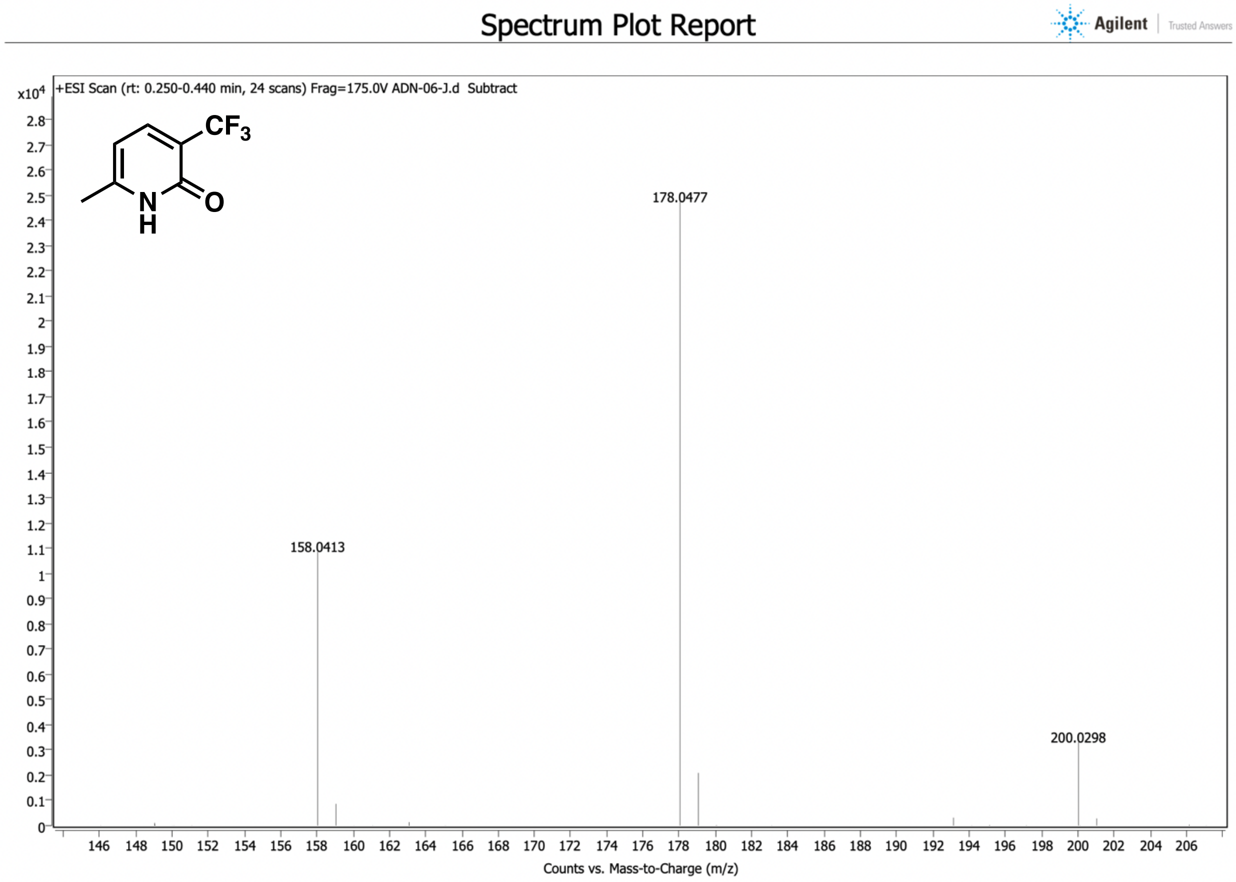

Spectrum Plot Report
Agilent | Trusted Answers
+ESI Scan (rt: 0.250-0.440 min, 24 scans) Frag=175.0V ADN-06-J.d Subtract
x10^4
CF3
N
H
O
178.0477
158.0413
200.0298
Counts vs. Mass-to-Charge (m/z)

(3c) 4,6-dimethyl-3-(trifluoromethyl)pyridin-2(1H)-one

3c - ^{1}H NMR (400 MHz, $CDCl_3$)

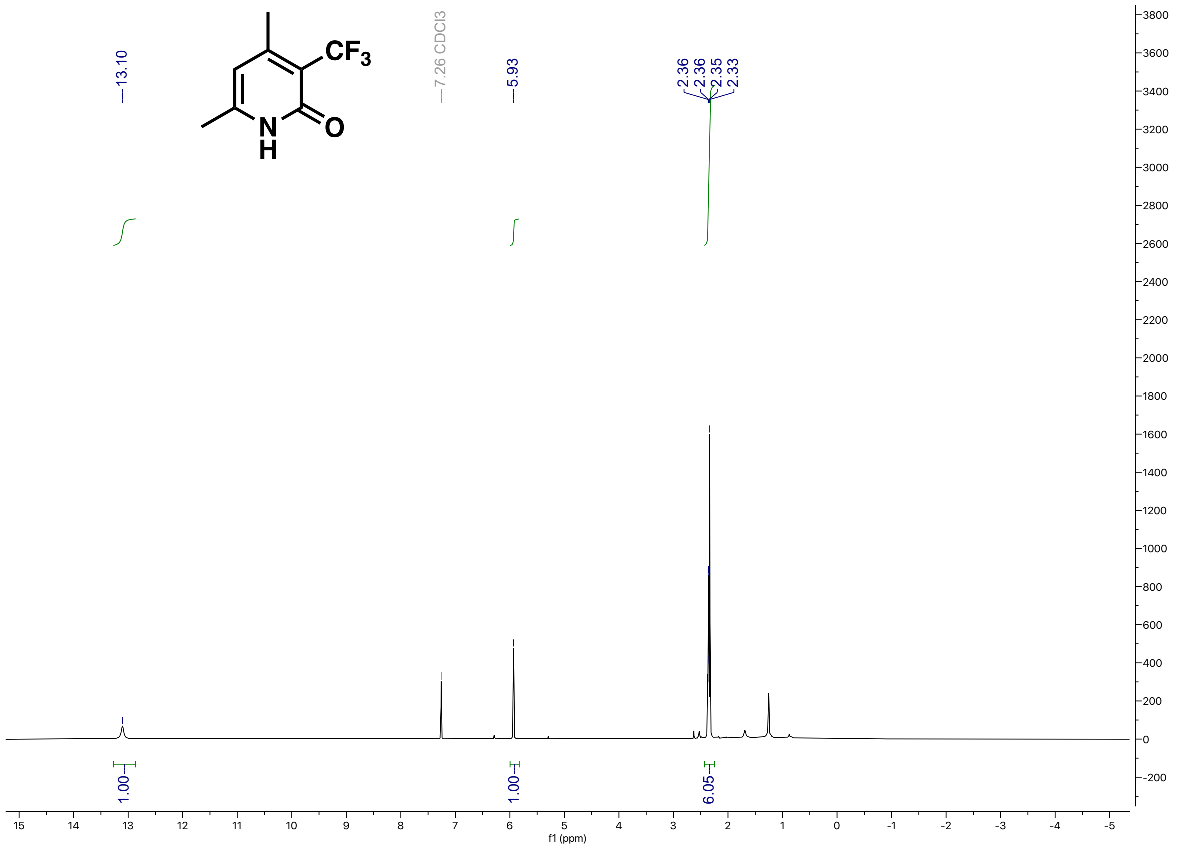

3c - ^{19}F NMR (376 MHz, $CDCl_3$)

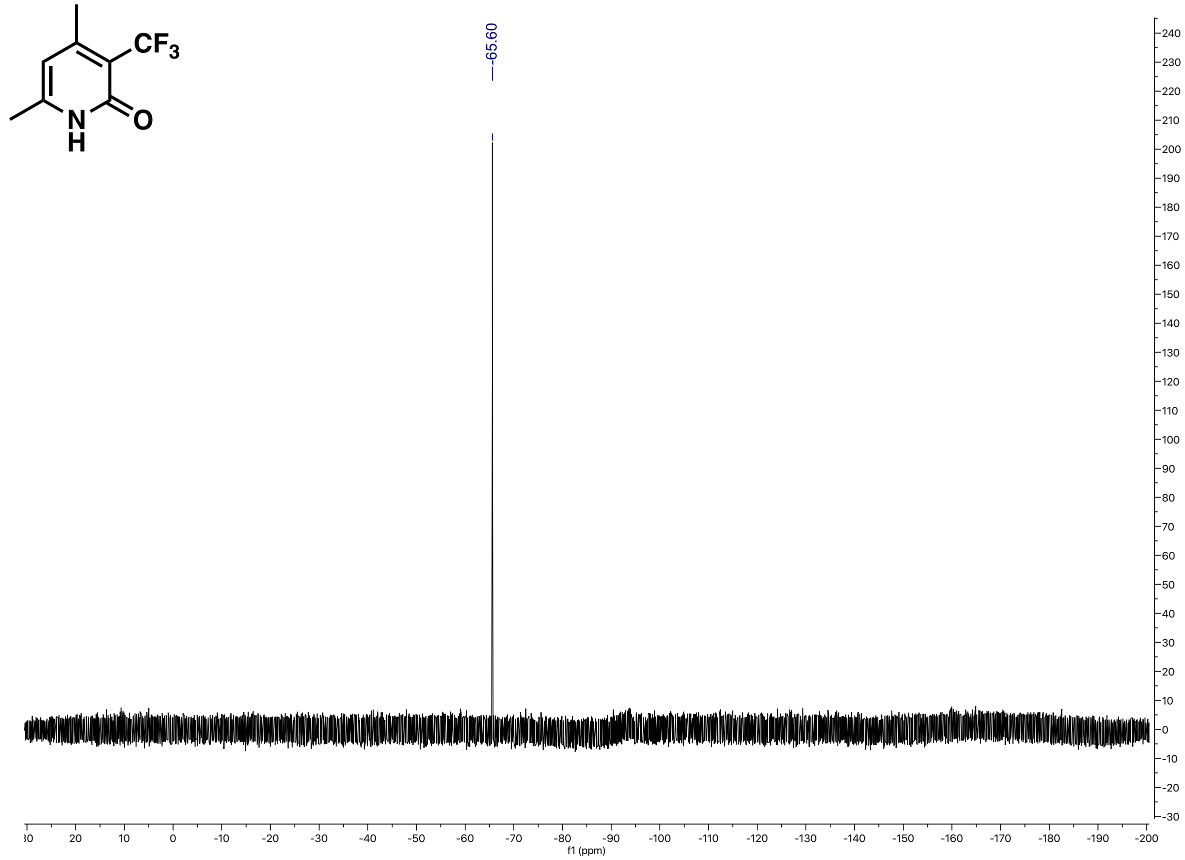

3c - ^{13}C NMR (126 MHz, $CDCl_3$)

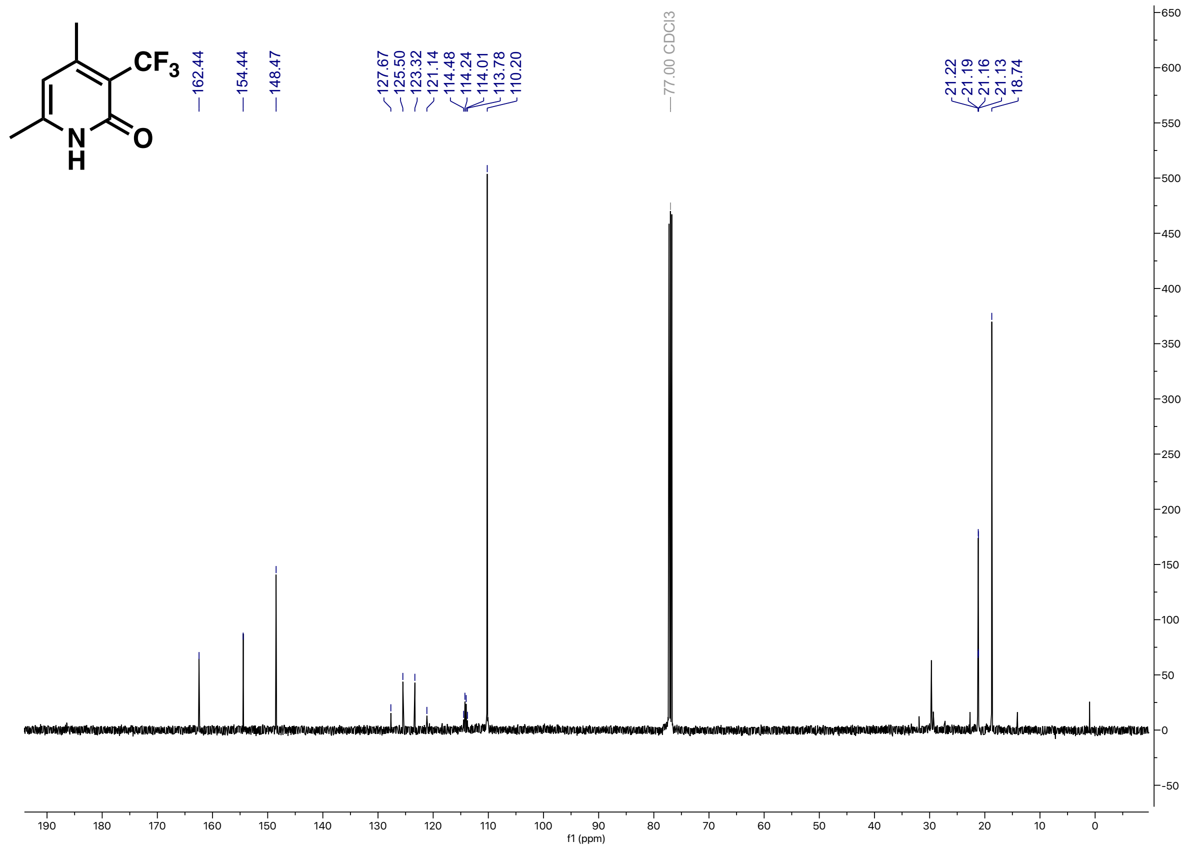

3c – HRMS (ESI)

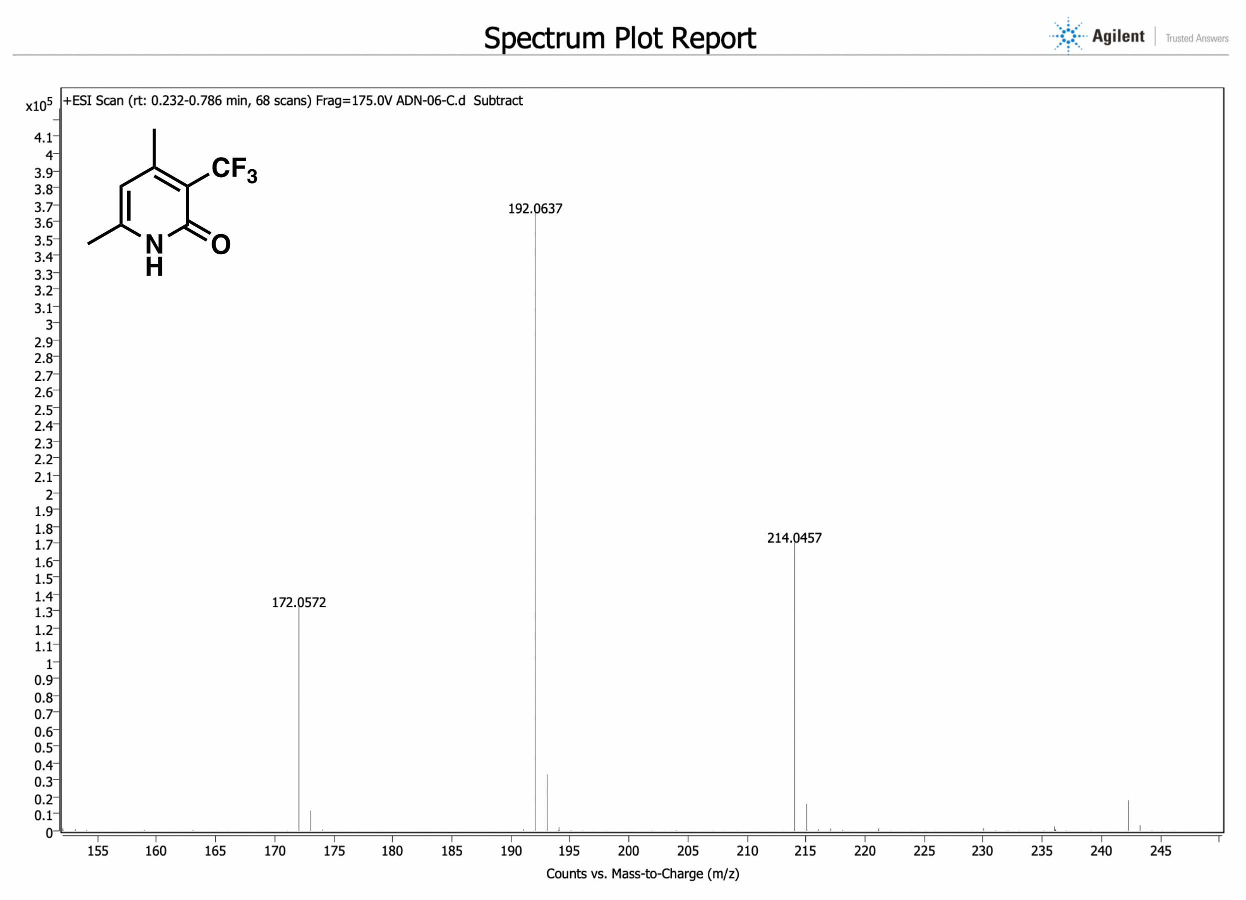

Spectrum Plot Report
Agilent
Trusted Answers
x10^5
+ESI Scan (rt: 0.232-0.786 min, 68 scans) Frag=175.0V ADN-06-C.d Subtract
CF3
N
H
O
192.0637
214.0457
172.0572
Counts vs. Mass-to-Charge (m/z)

(3d) 3-methyl-5-(trifluoromethyl)pyridin-2(1H)-one

3d - ^{1}H NMR (400 MHz, $CDCl_3$)

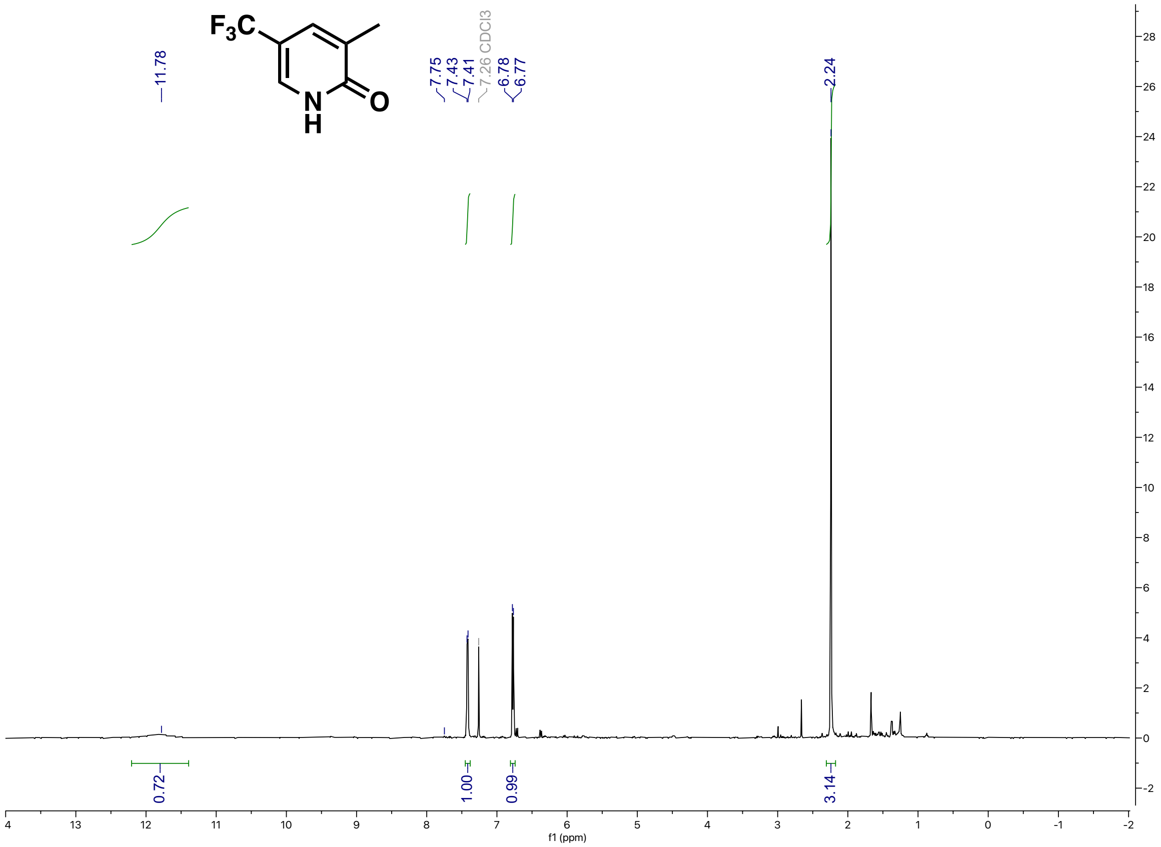

3d - ^{19}F NMR (376 MHz, $CDCl_3$)

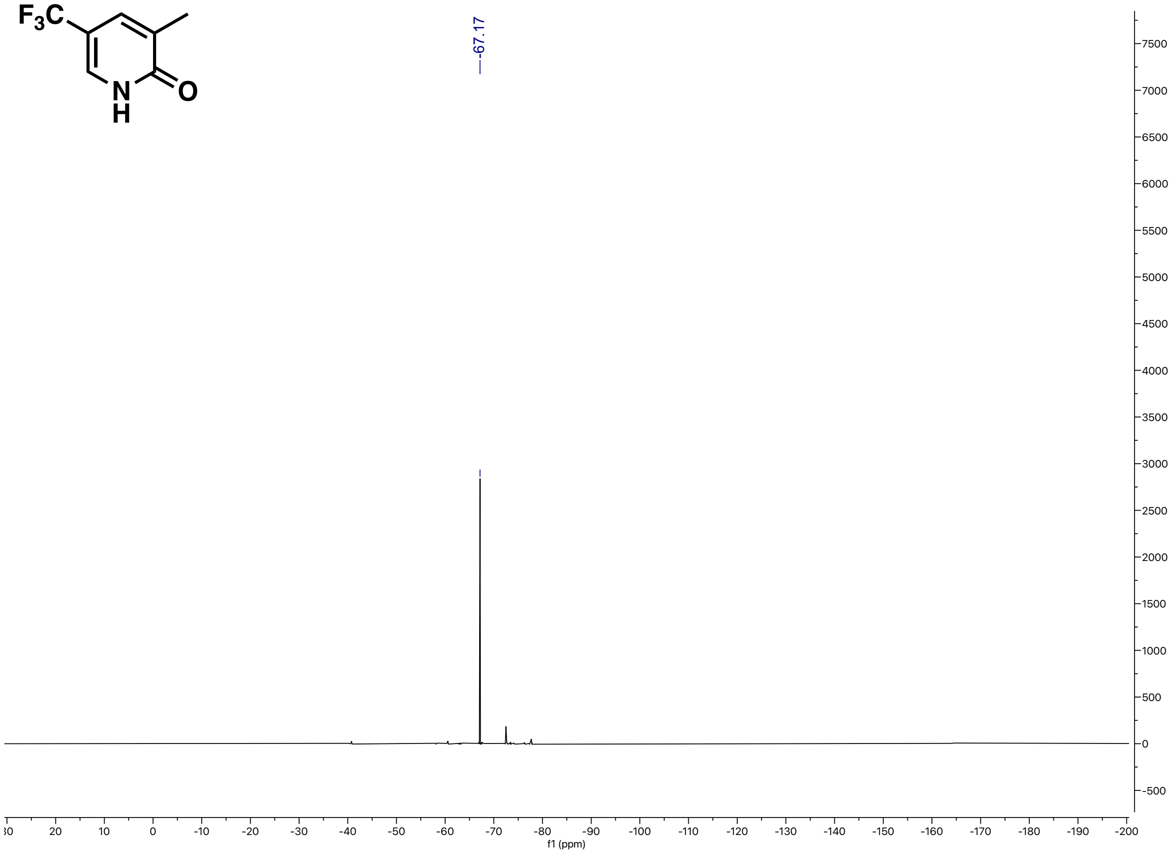

3d - ^{13}C NMR (126 MHz, $CDCl_3$)

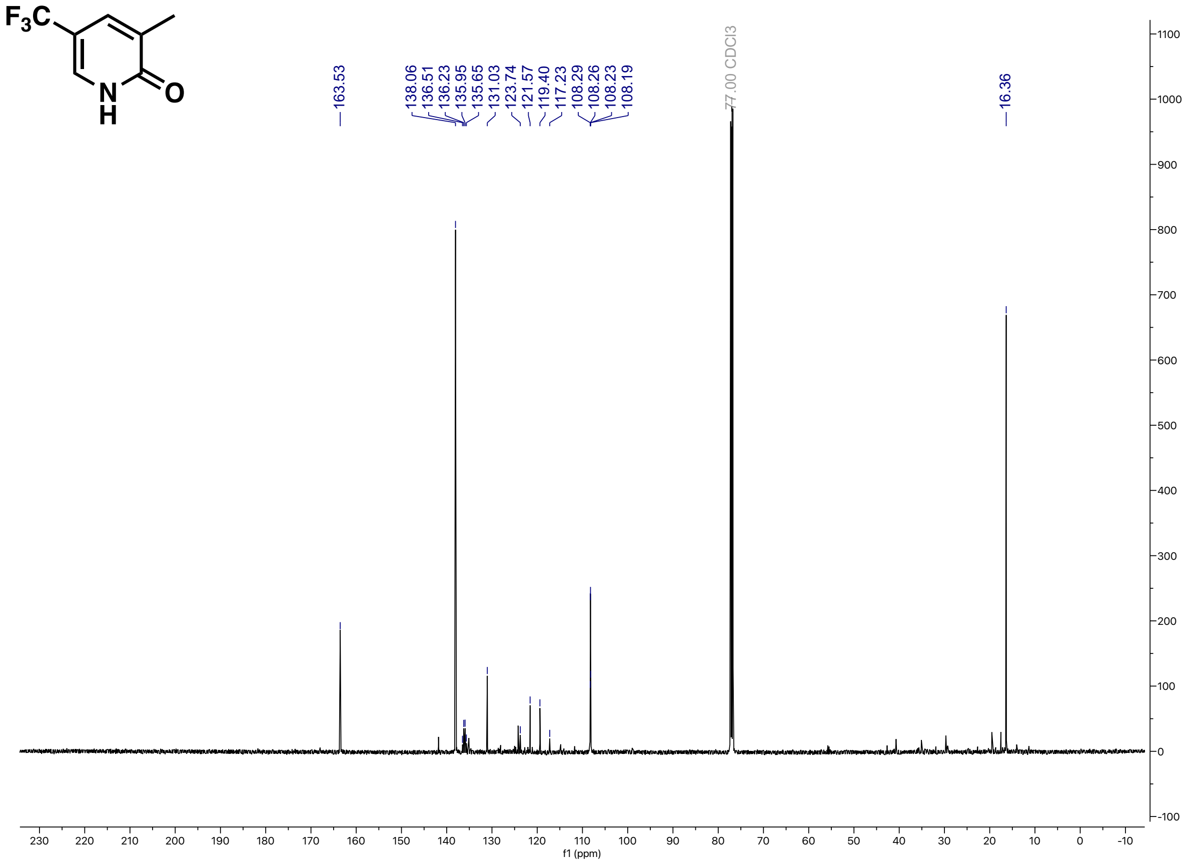

3d – HRMS (ESI)

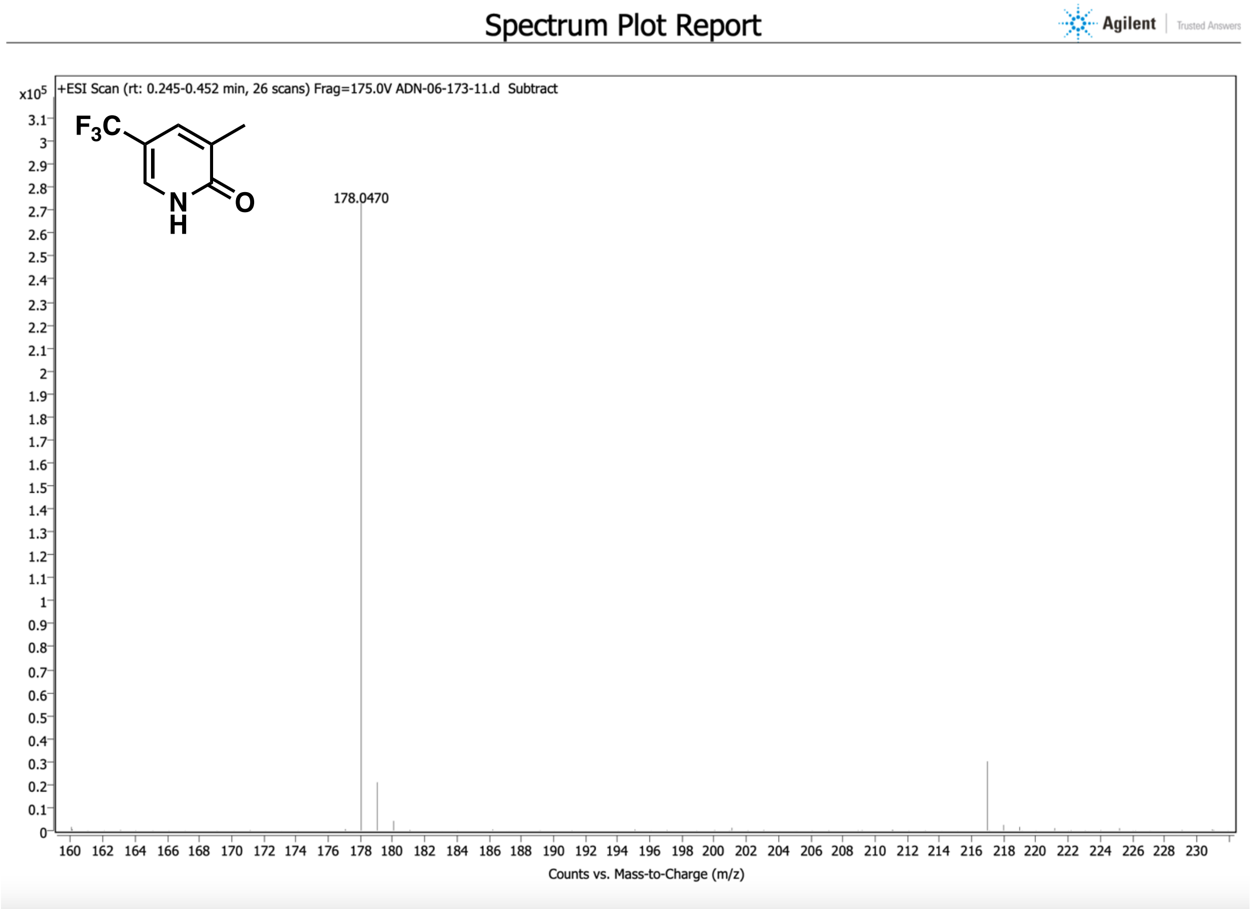

Spectrum Plot Report
Agilent
Trusted Answers
+ESI Scan (rt: 0.245-0.452 min, 26 scans) Frag=175.0V ADN-06-173-11.d Subtract
x10^5
178.0470
F3C
N
H
O
Counts vs. Mass-to-Charge (m/z)

(3e) 1-methyl-3-(trifluoromethyl)pyridin-2(1H)-one

This compound is also described in *Chem. Commun*. **2018**, *54*, 10574-10577

3e – ^{1}H NMR (400 MHz, $CDCl_3$)

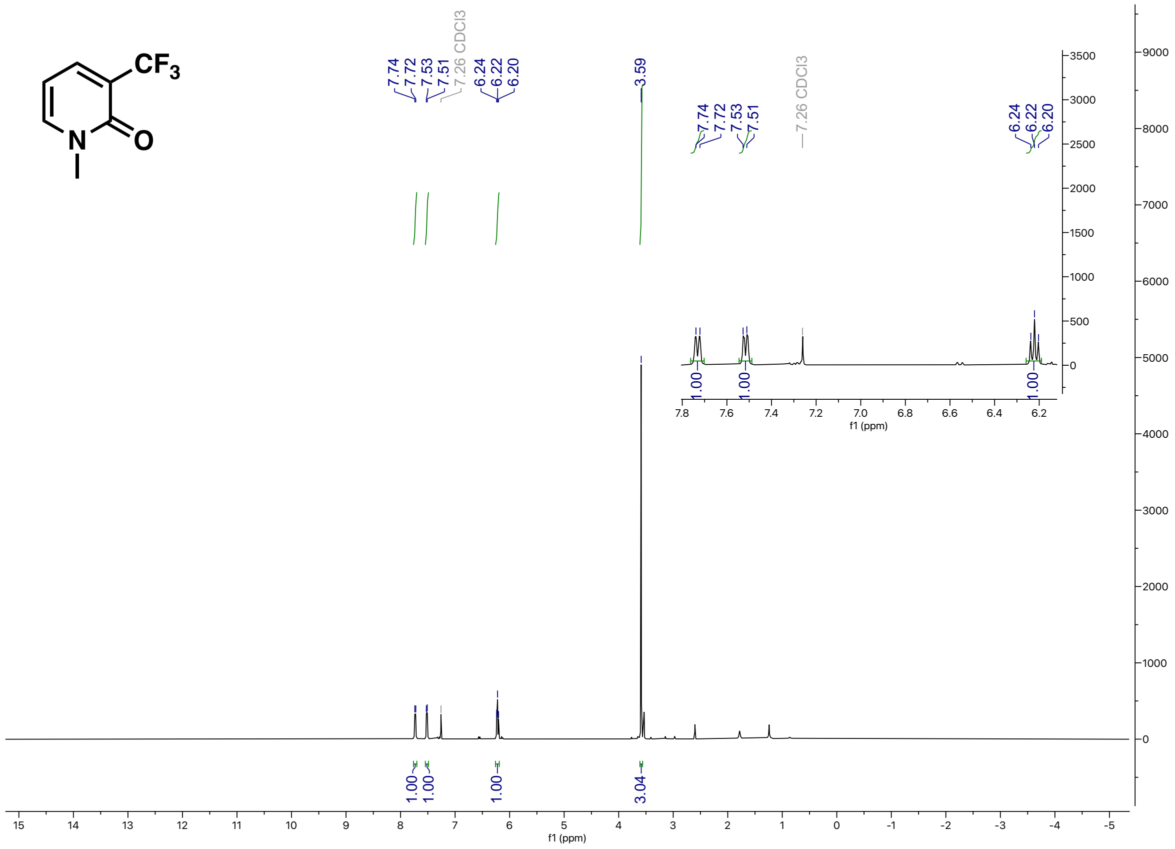

3e – ^{19}F NMR (376 MHz, $CDCl_3$)

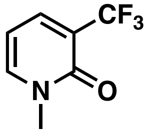

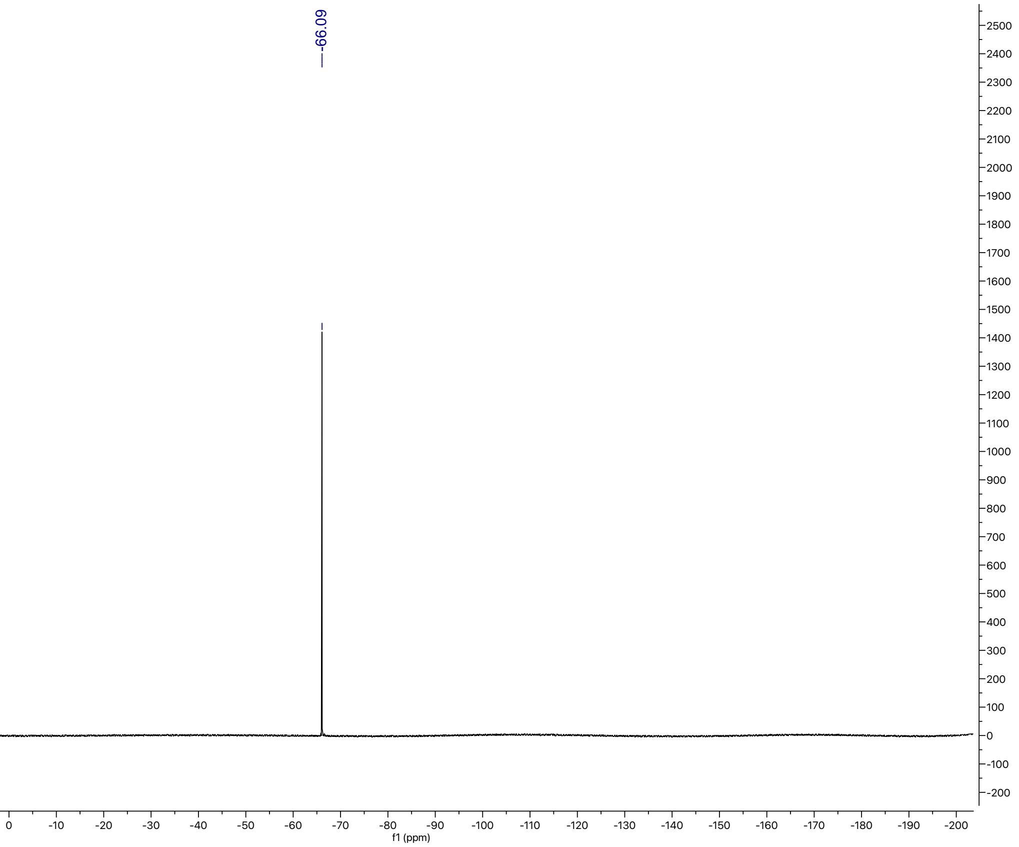

3e – ^{13}C NMR (126 MHz, $CDCl_3$)

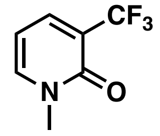

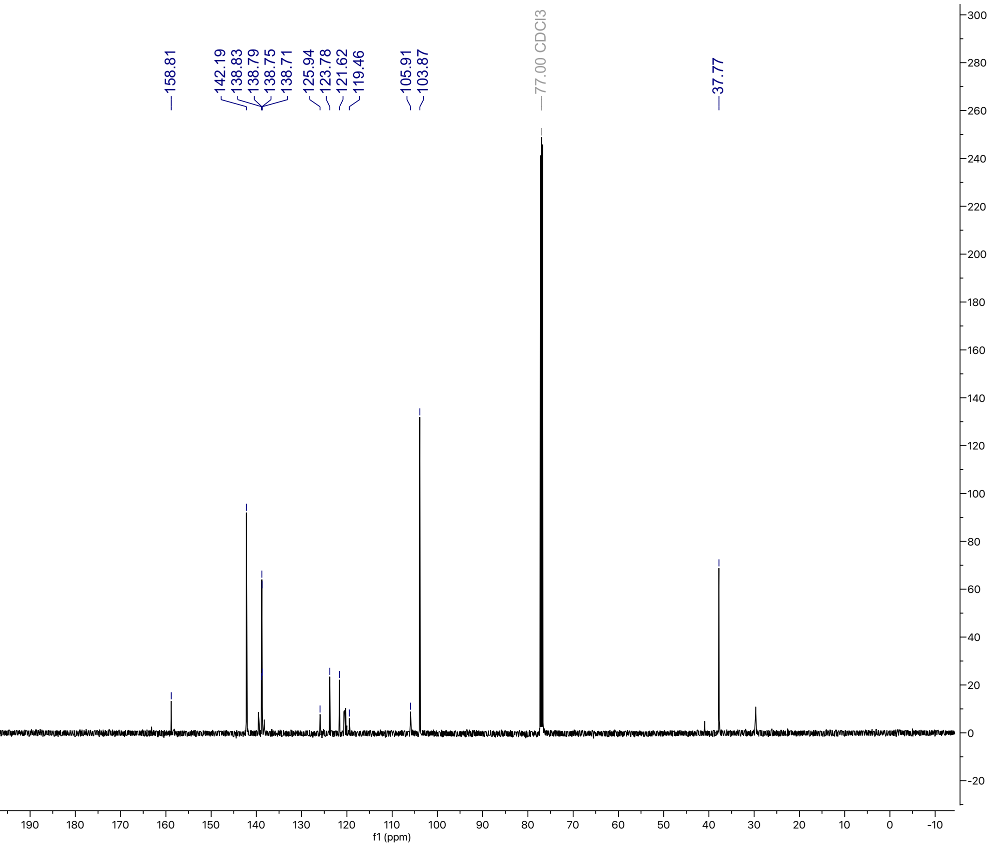

3e – HRMS (ESI)

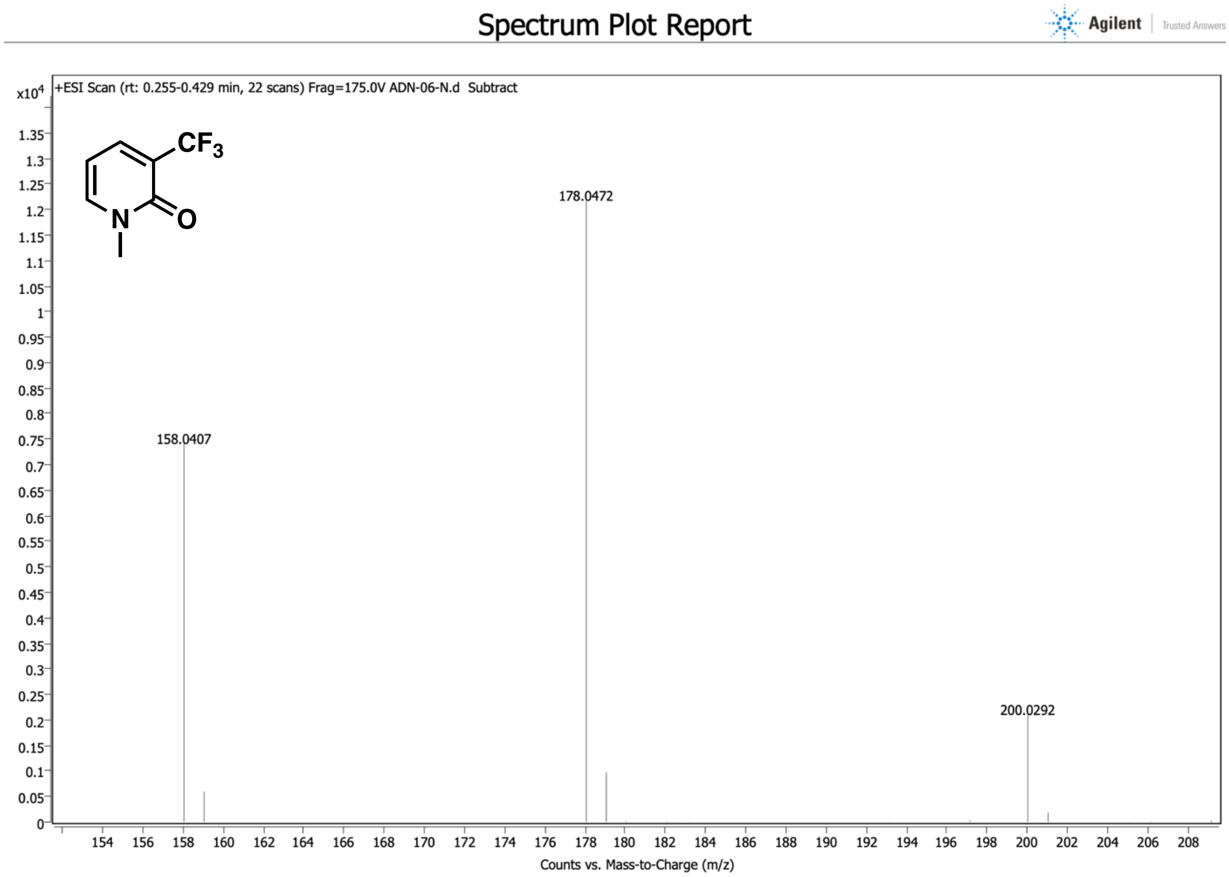

Spectrum Plot Report
Agilent
Trusted Answers
x10^4
+ESI Scan (rt: 0.255-0.429 min, 22 scans) Frag=175.0V ADN-06-N.d Subtract
CF3
N
O
178.0472
158.0407
200.0292
Counts vs. Mass-to-Charge (m/z)

(3f) 1-cyclopropyl-3-(trifluoromethyl)pyridin-2(1H)-one

1f – ^{1}H NMR (400MHz, CD_2Cl2), synthesized using previously published methods (see S13)

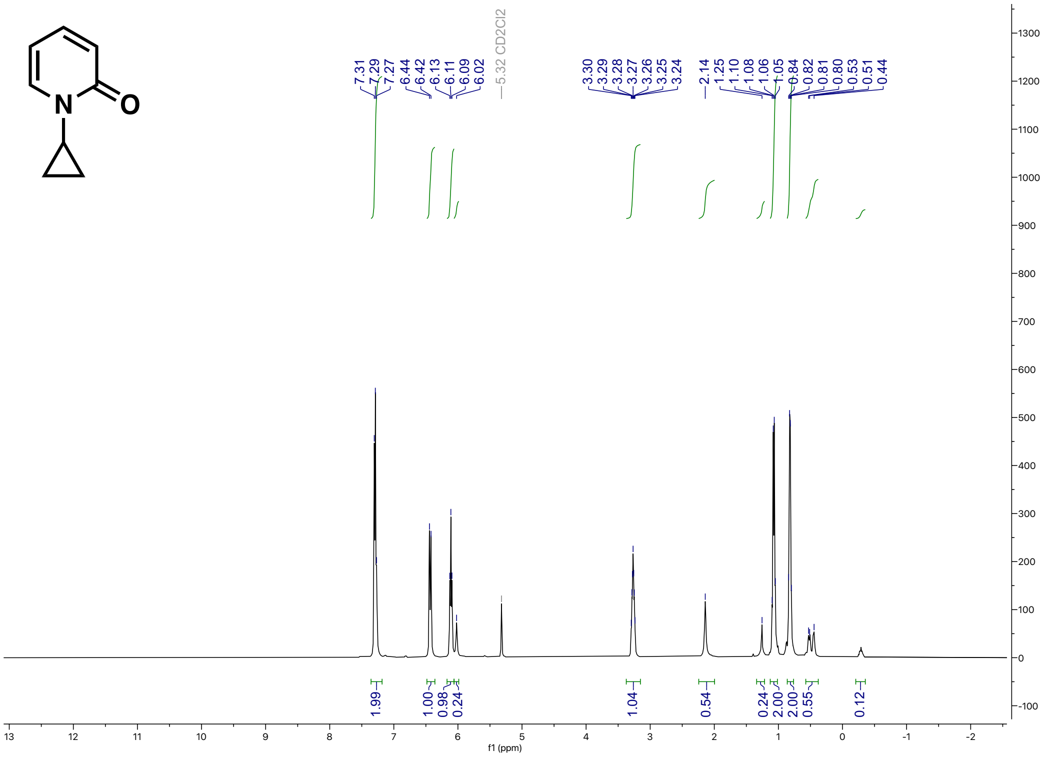

3f – ^{1}H NMR (500 MHz, $CDCl_3$)

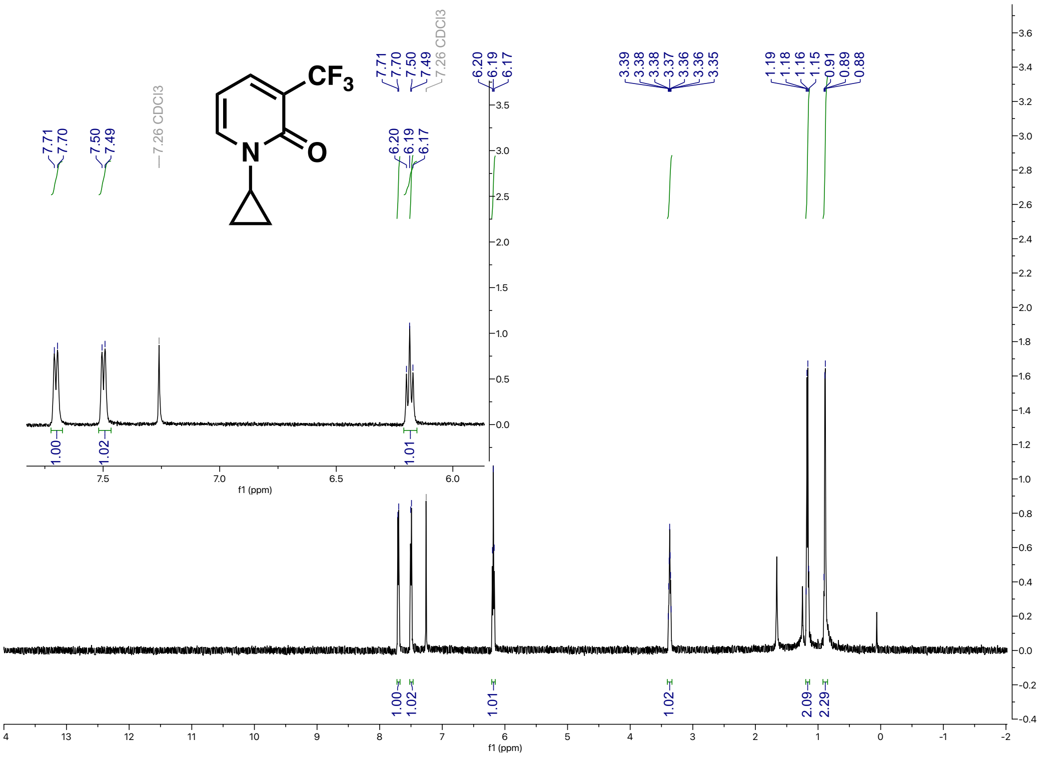

3f – ^{19}F NMR (376 MHz, $CDCl_3$)

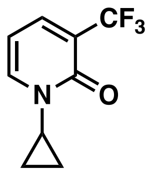

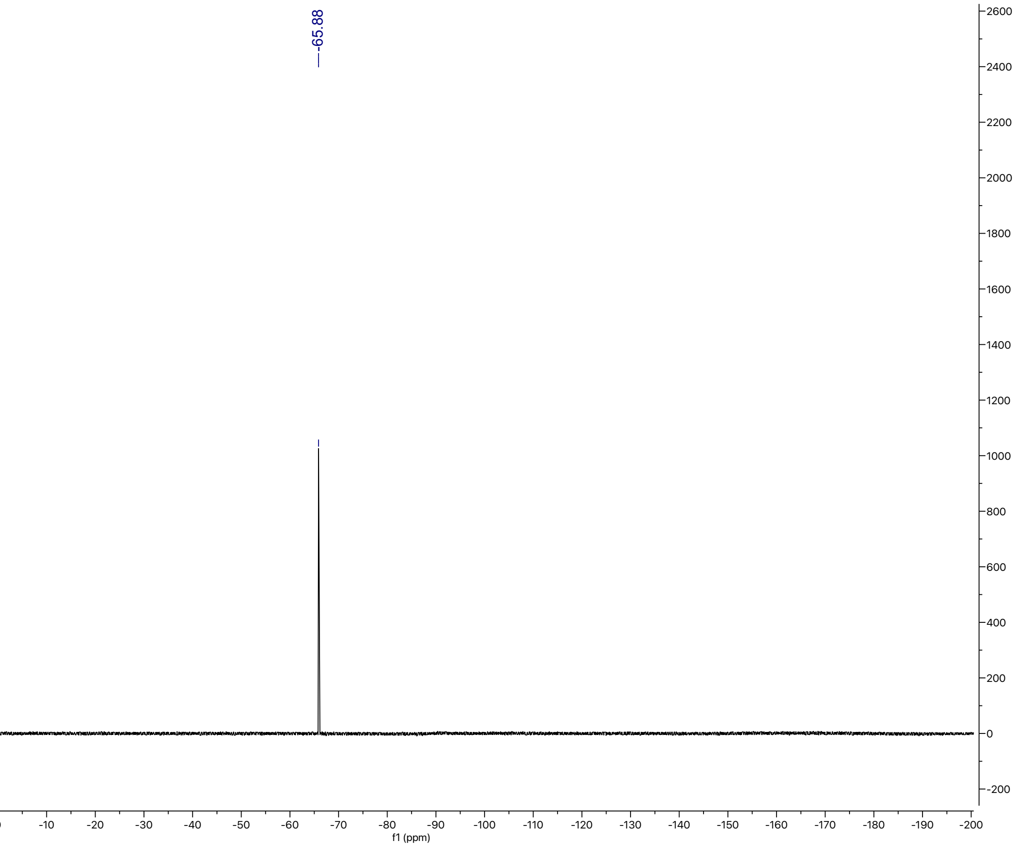

3f – ^{13}C NMR (126 MHz, $CDCl_3$)

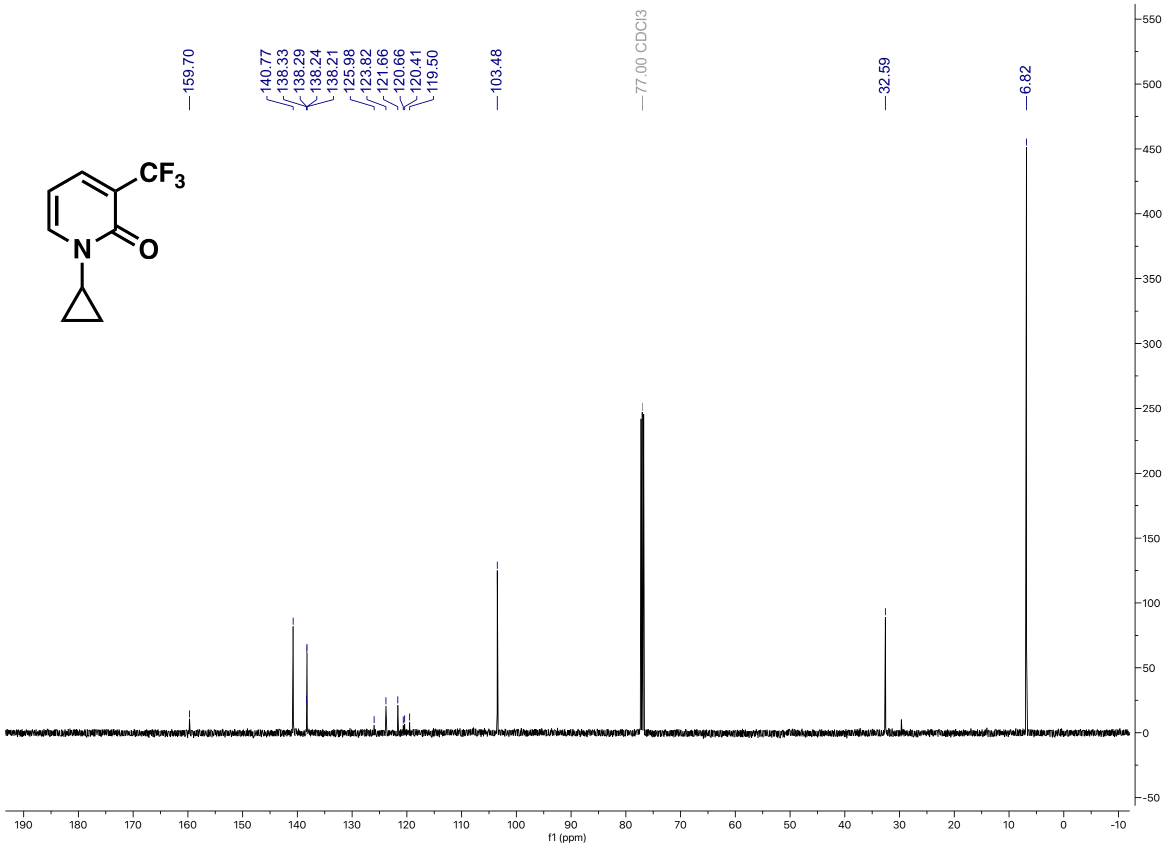

3f – HRMS (ESI)

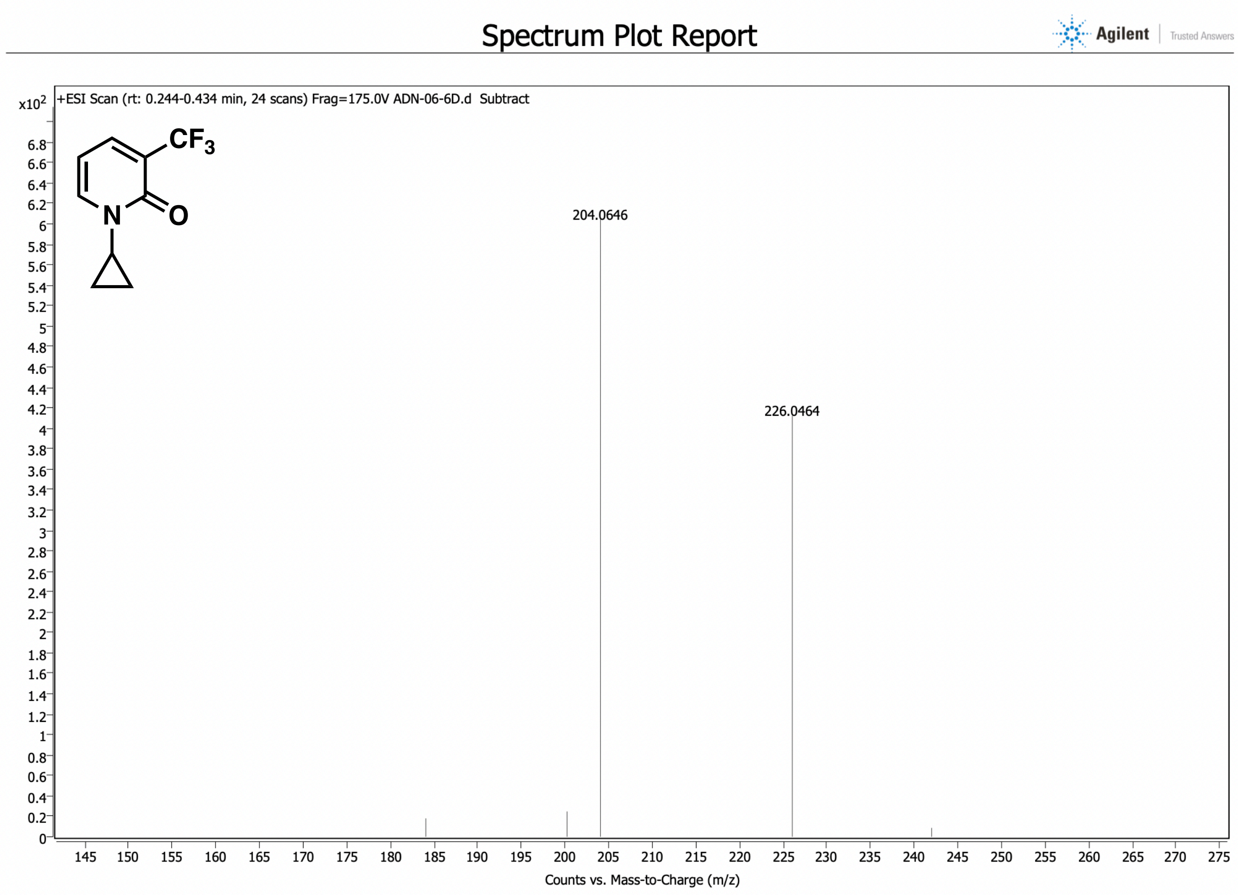

Spectrum Plot Report
Agilent
Trusted Answers
x10^2
+ESI Scan (rt: 0.244-0.434 min, 24 scans) Frag=175.0V ADN-06-6D.d Subtract
CF3
N
O
204.0646
226.0464
Counts vs. Mass-to-Charge (m/z)

(3g) 3-(1,1-difluoroethyl)pyridine-2(1H)-one

3g – ^{1}H NMR (400 MHz, $CDCl_3$)

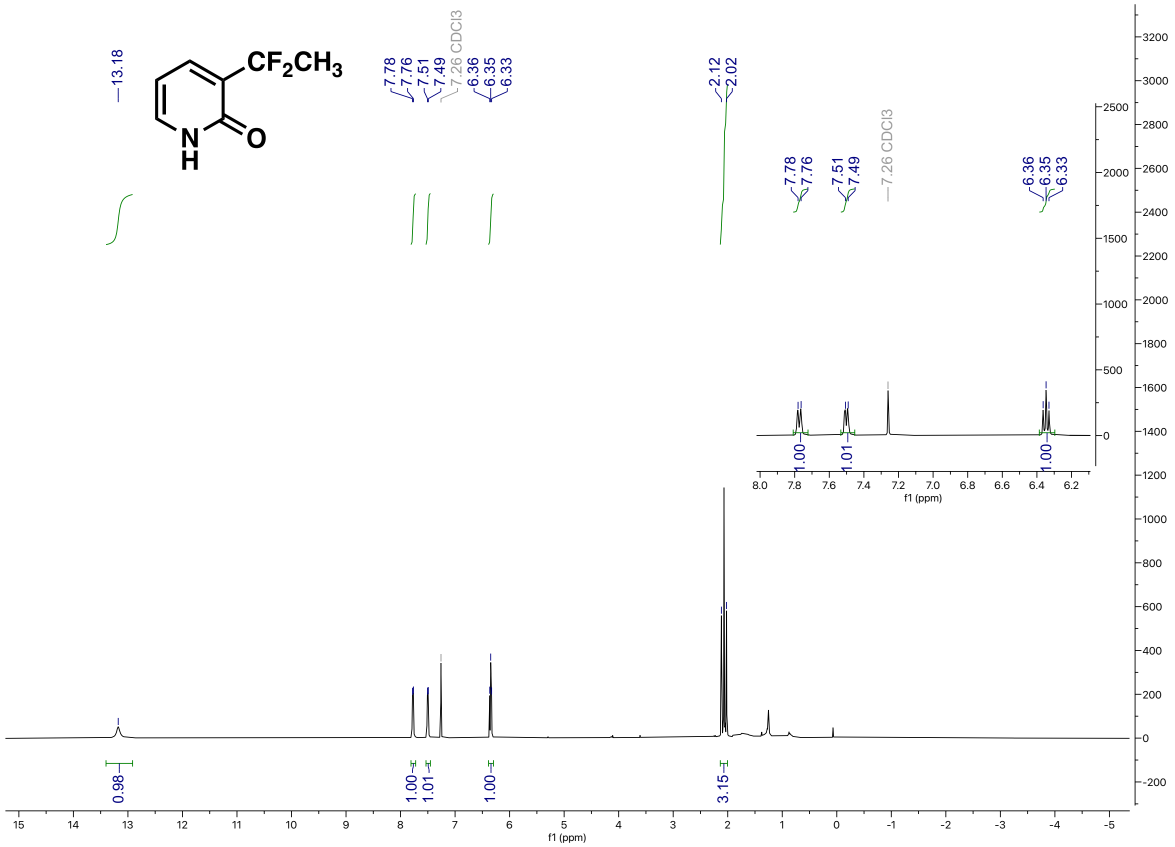

3g – ^{19}F NMR (376 MHz, $CDCl_3$)

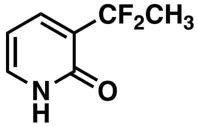

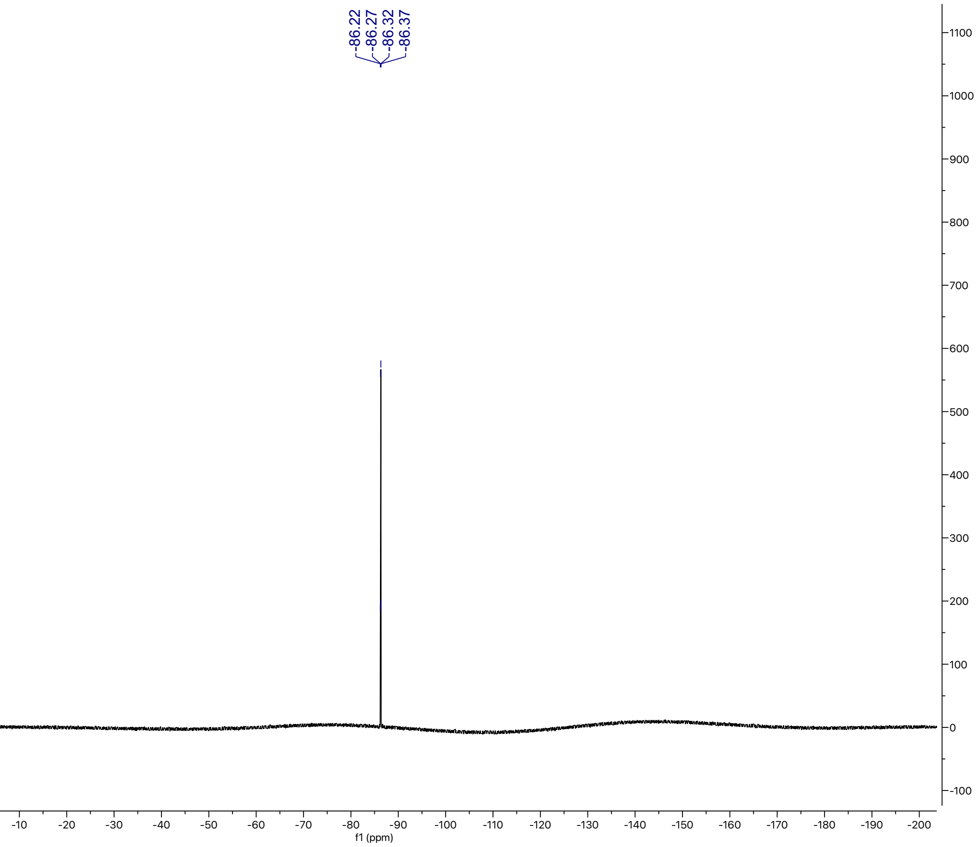

3g – ^{13}C NMR (126 MHz, $CDCl_3$)

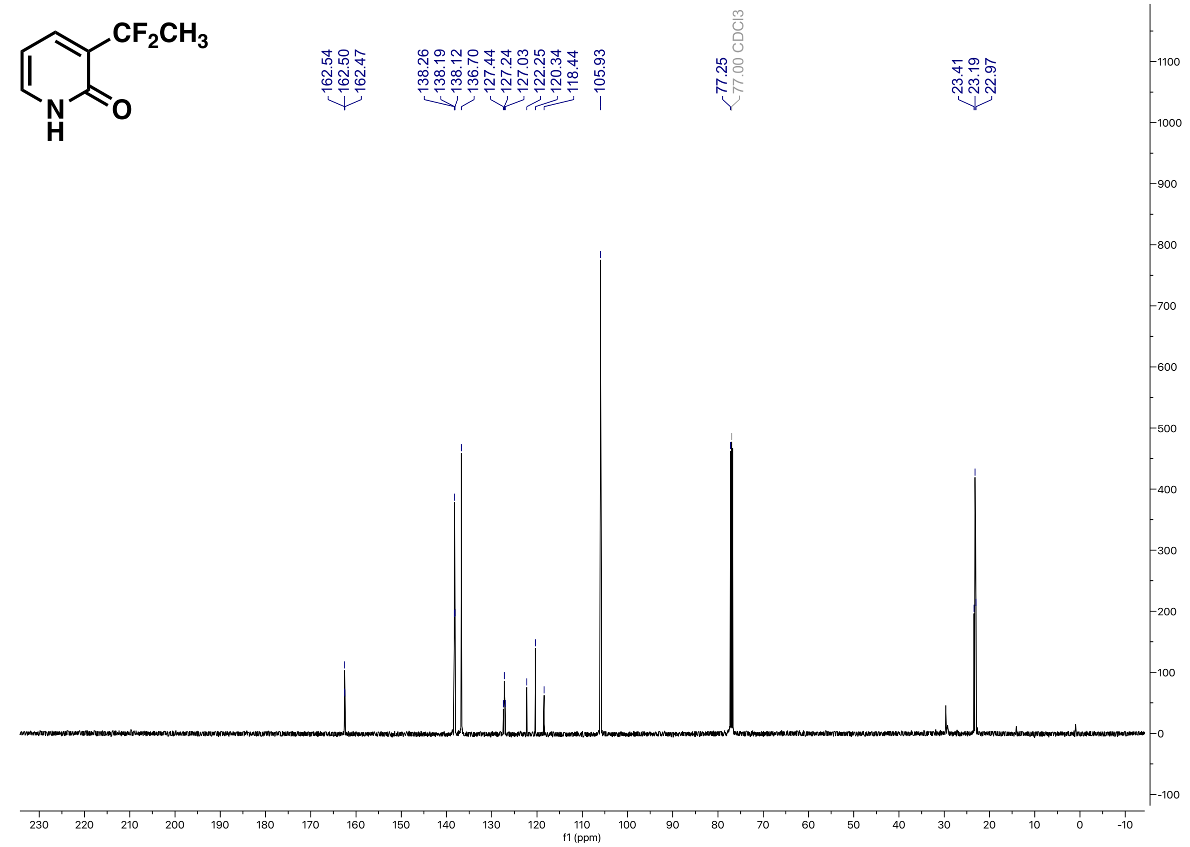

3g – HRMS (ESI)

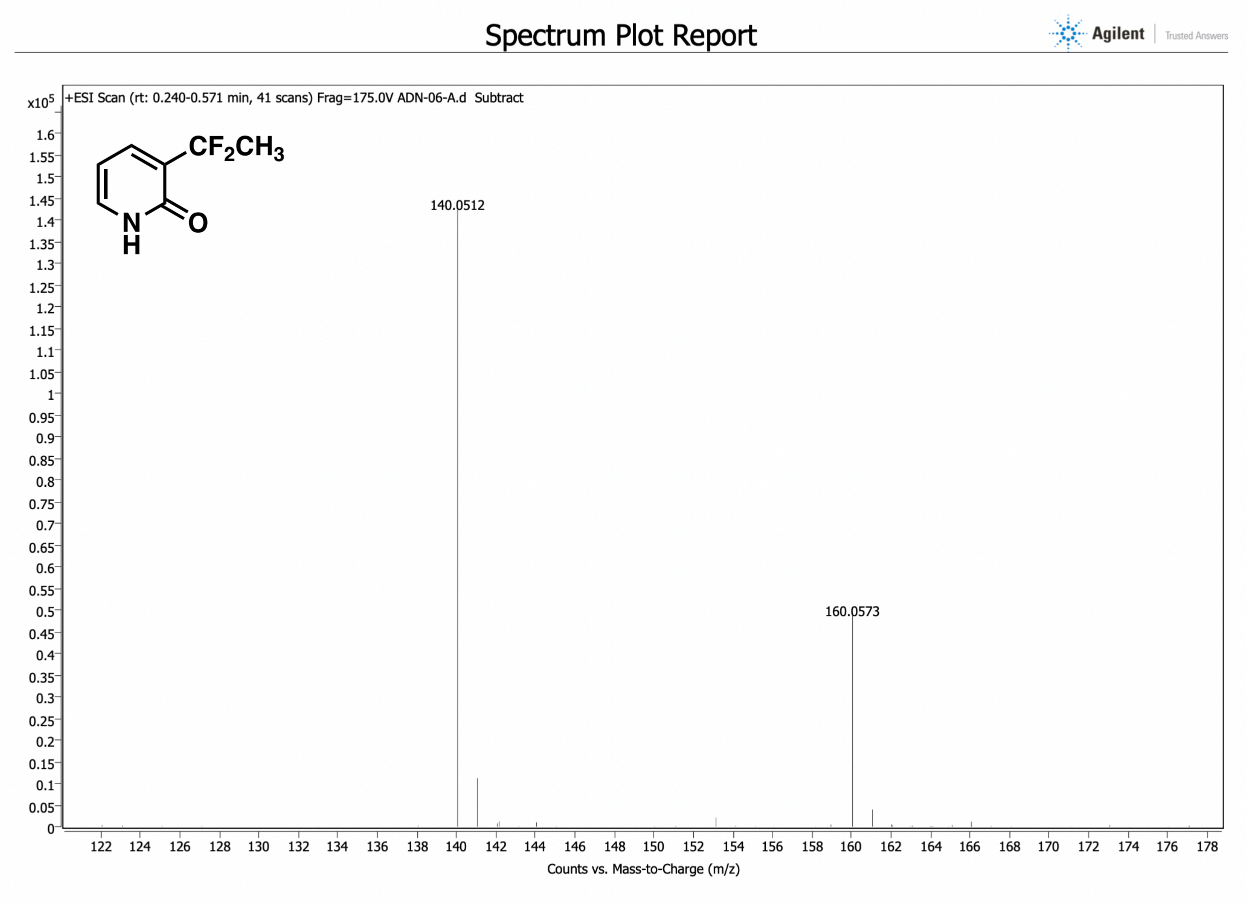

Spectrum Plot Report
Agilent
Trusted Answers
+ESI Scan (rt: 0.240-0.571 min, 41 scans) Frag=175.0V ADN-06-A.d Subtract
x10^5
CF2CH3
N
H
O
140.0512
160.0573
Counts vs. Mass-to-Charge (m/z)

(3h) 3-(difluoromethyl)pyridin-2(1H)-one

3h – ^{1}H NMR (500 MHz, $CDCl_3$)

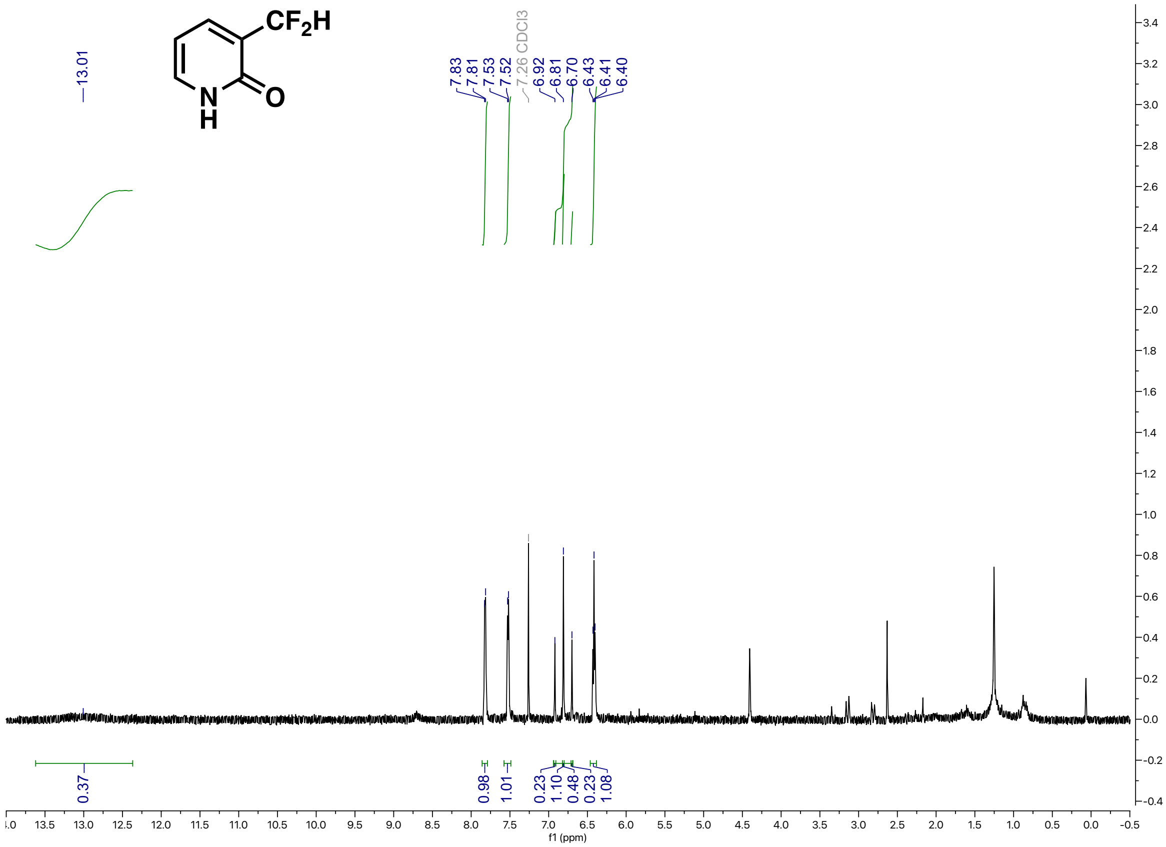

3h – ^{19}F NMR (376 MHz, $CDCl_3$)

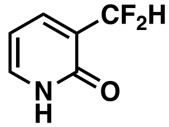

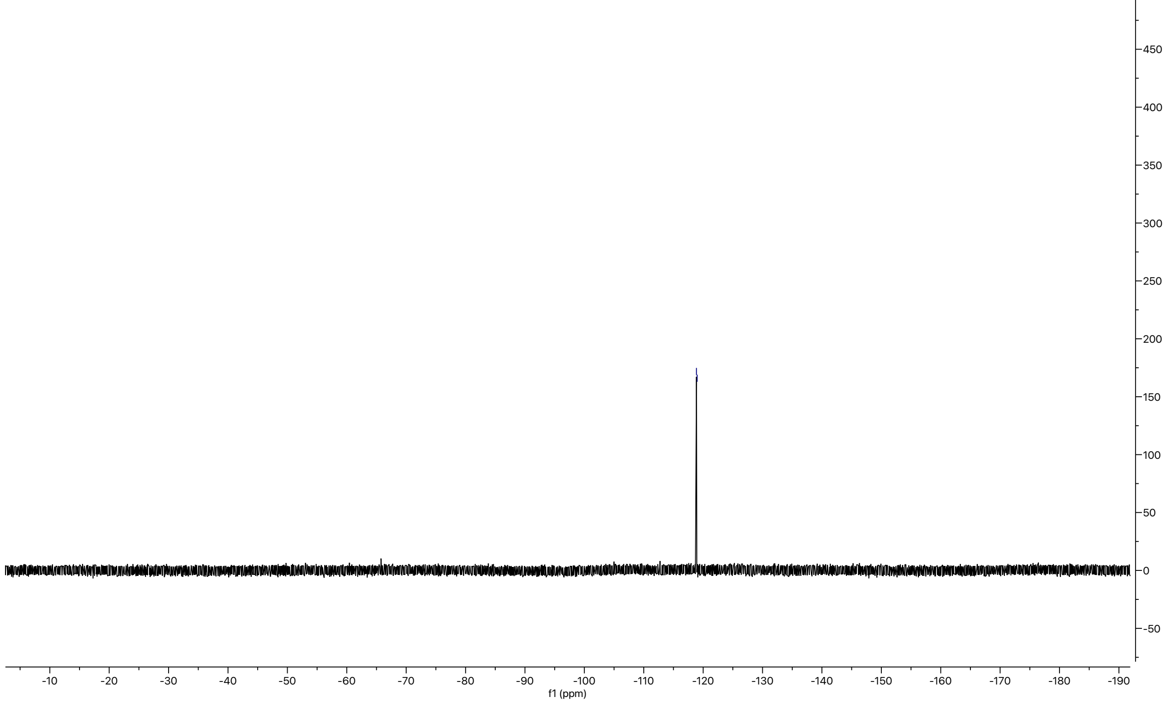

3h – ^{13}C NMR (126 MHz, $CDCl_3$)

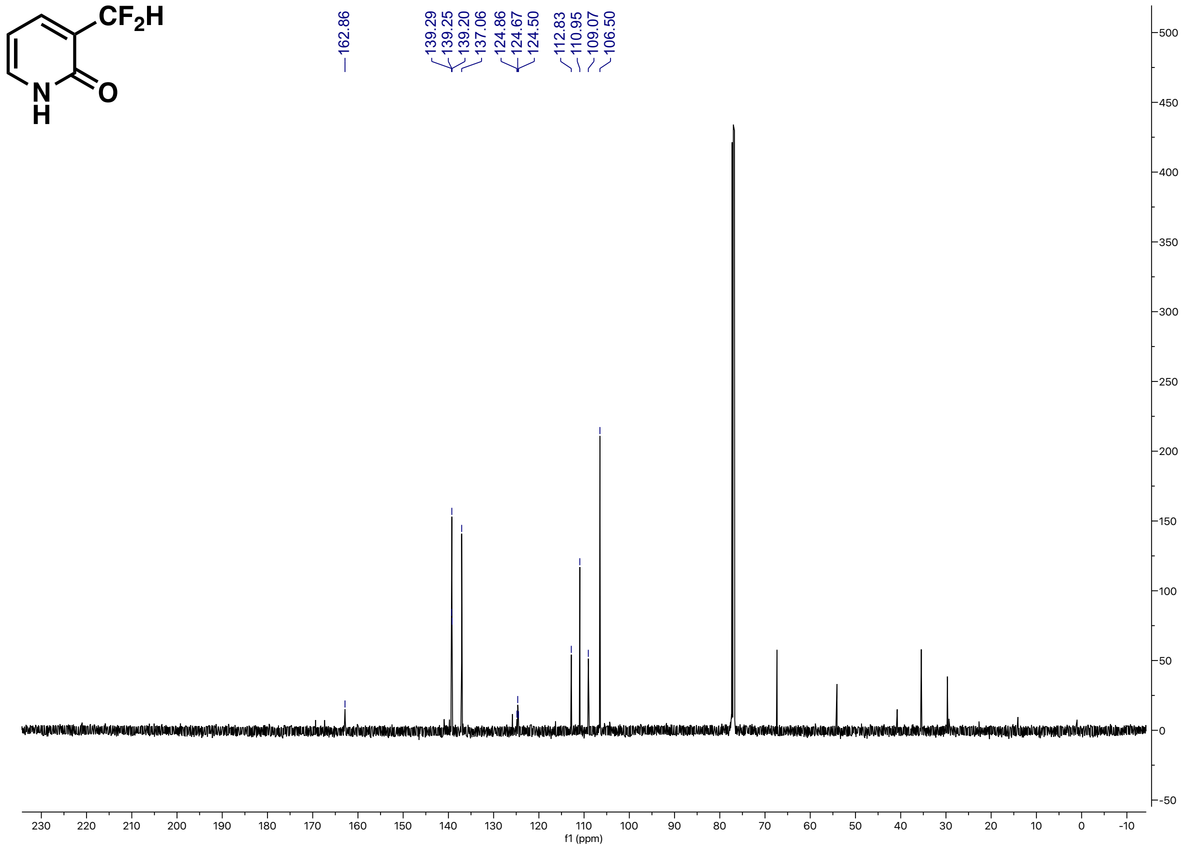

3h – HRMS (ESI)

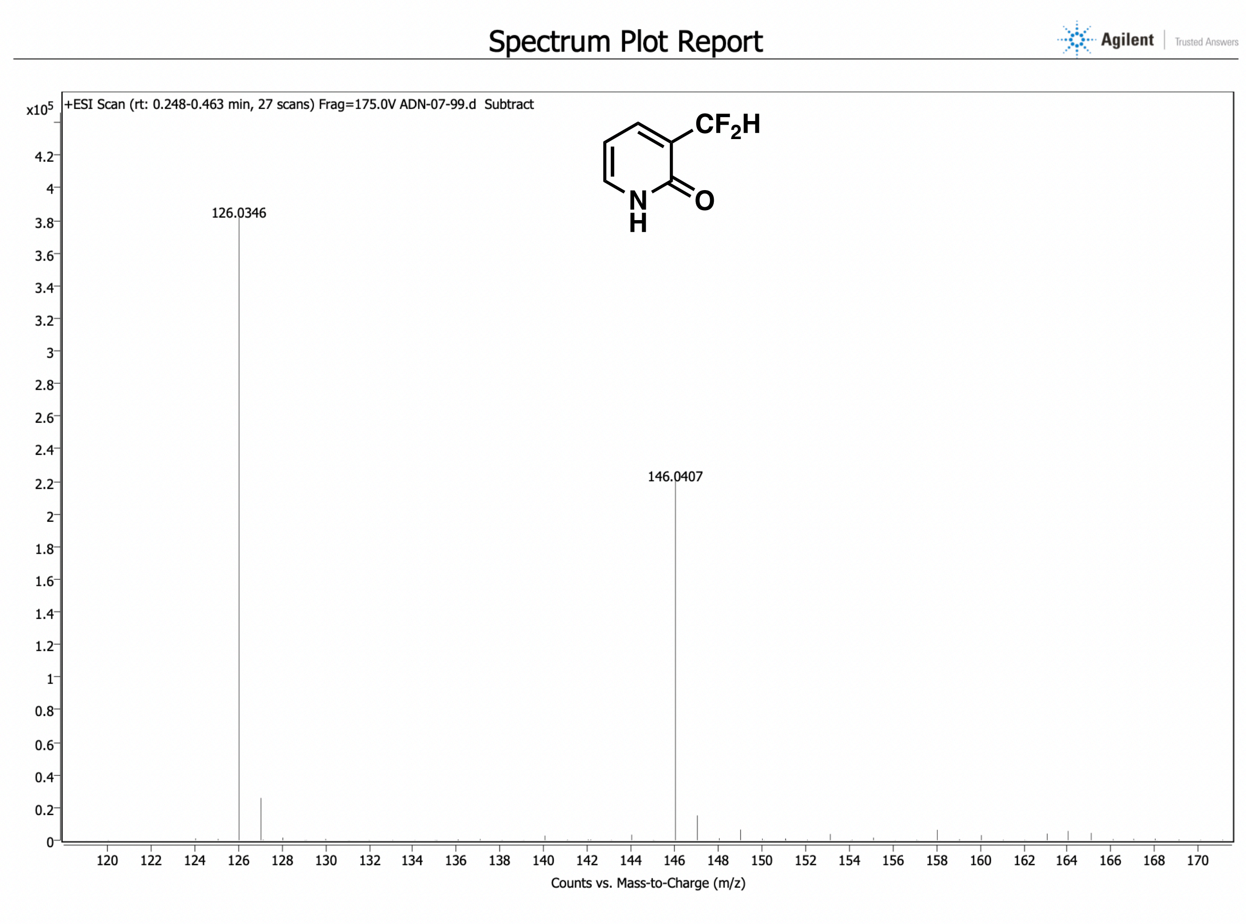

Spectrum Plot Report
Agilent
Trusted Answers
x10^5
+ESI Scan (rt: 0.248-0.463 min, 27 scans) Frag=175.0V ADN-07-99.d Subtract
CF2H
N
H
O
126.0346
146.0407
Counts vs. Mass-to-Charge (m/z)

(3i) 3-(2-(4-bromophenyl)-1,1-difluoromethyl)pyridin-2(1H)-one

3i – ^{1}H NMR (400 MHz, $CDCl_3$)

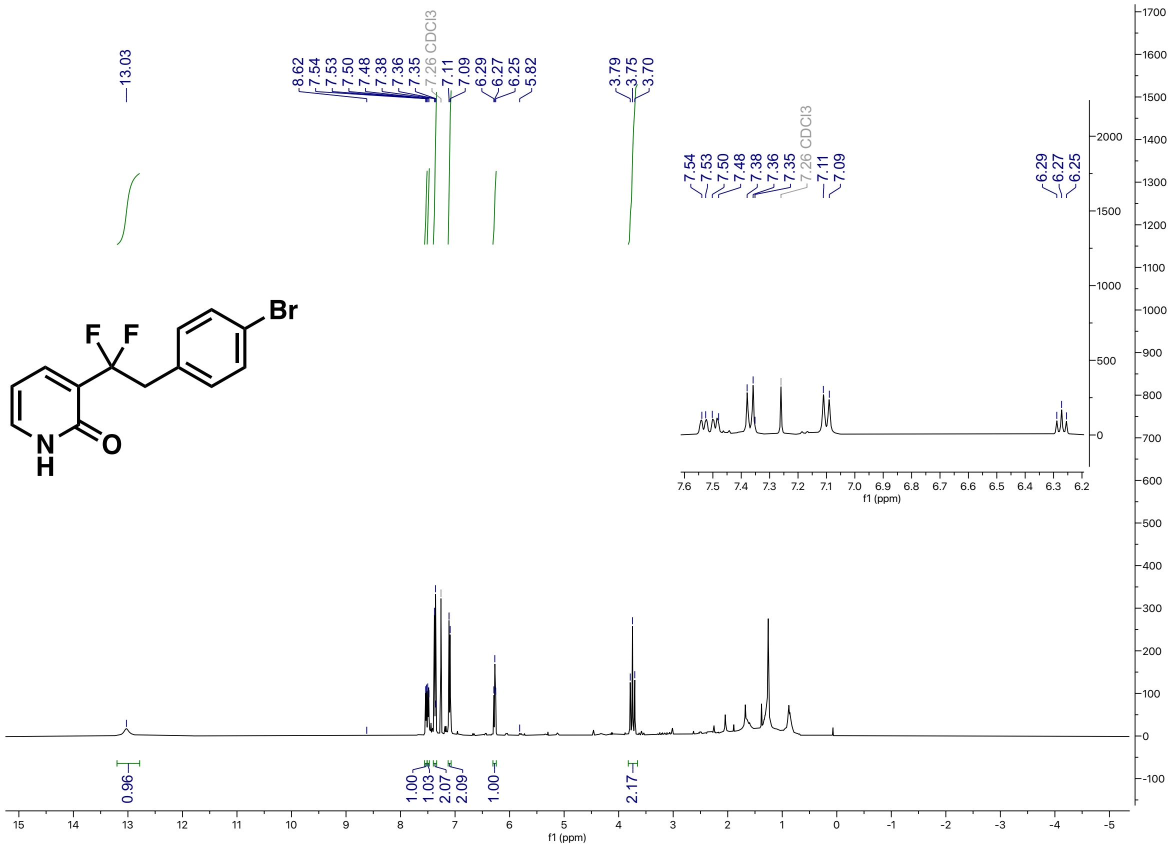

3i – ^{19}F NMR (376 MHz, $CDCl_3$)

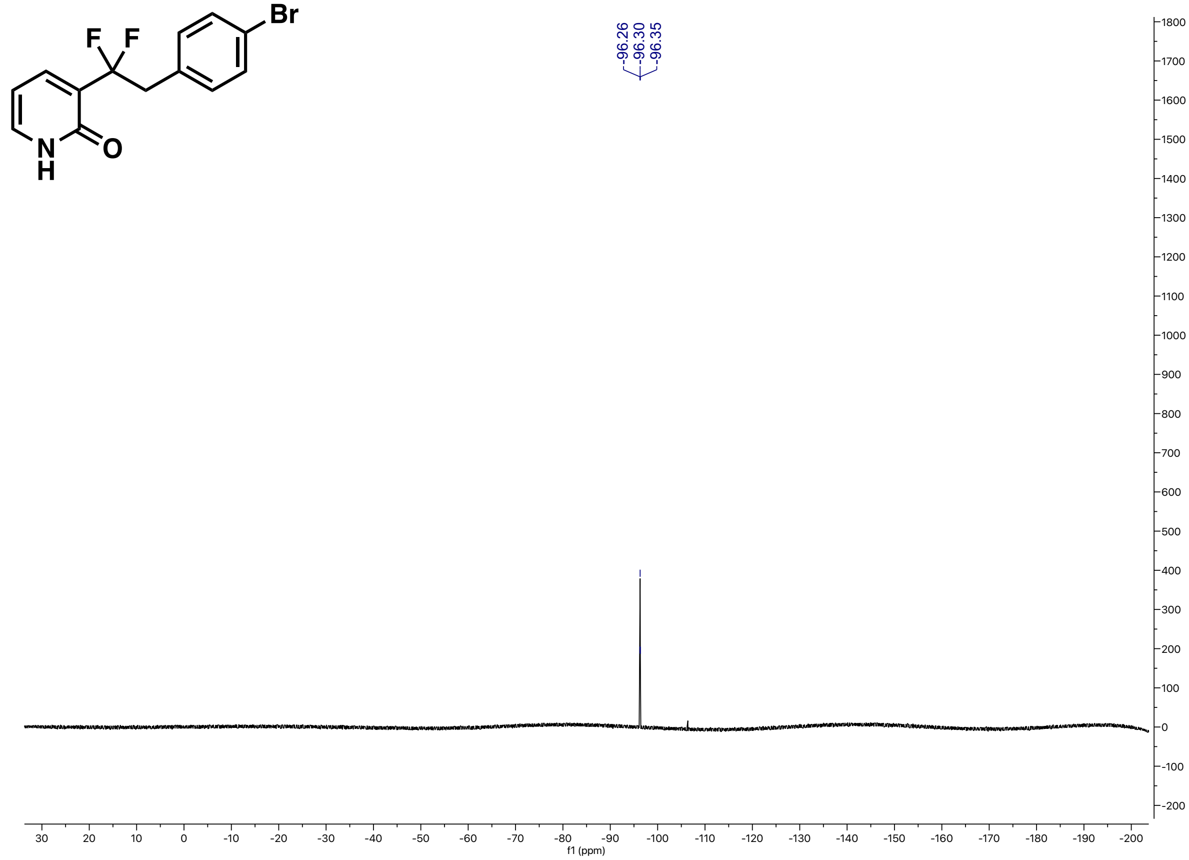

3i – ^{13}C NMR (126 MHz, $CDCl_3$)

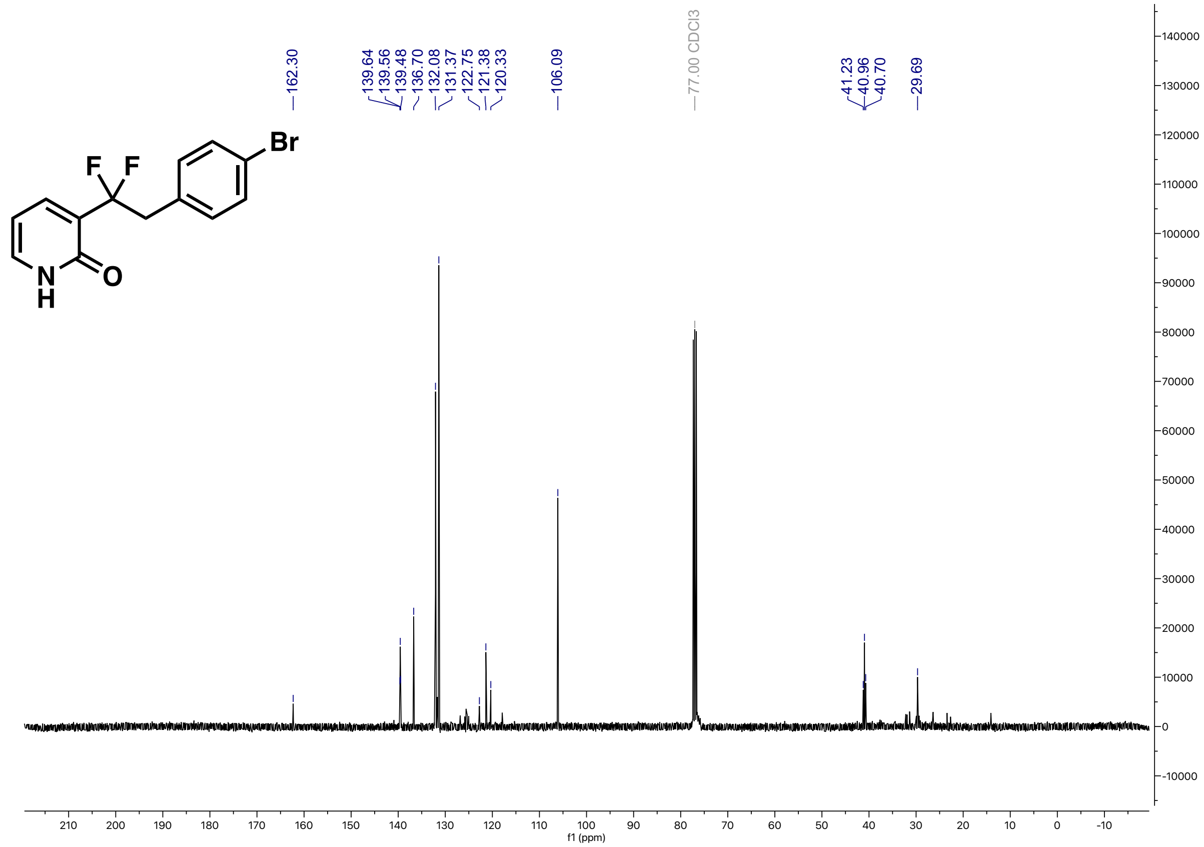

3i – HRMS (ESI)

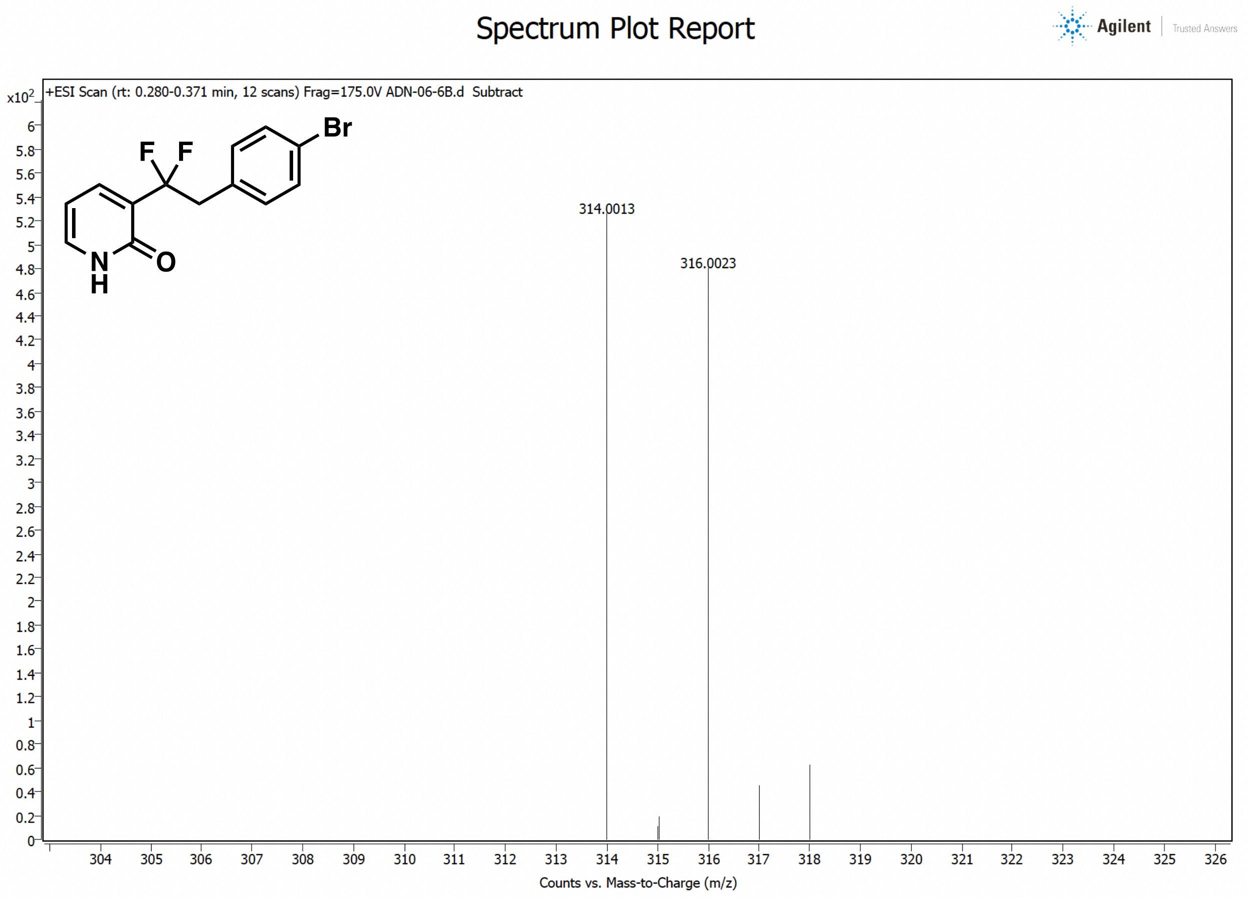

Spectrum Plot Report
Agilent
Trusted Answers
x10^2
+ESI Scan (rt: 0.280-0.371 min, 12 scans) Frag=175.0V ADN-06-6B.d Subtract
F
F
Br
N
H
O
314.0013
316.0023
6
5.8
5.6
5.4
5.2
5
4.8
4.6
4.4
4.2
4
3.8
3.6
3.4
3.2
3
2.8
2.6
2.4
2.2
2
1.8
1.6
1.4
1.2
1
0.8
0.6
0.4
0.2
0
304
305
306
307
308
309
310
311
312
313
314
315
316
317
318
319
320
321
322
323
324
325
326
Counts vs. Mass-to-Charge (m/z)

(3j) 3-(trifluoromethyl)pyridin-4(1H)-one

3j - ^{1}H NMR (500 MHz, $CO(CD_3)_2$)

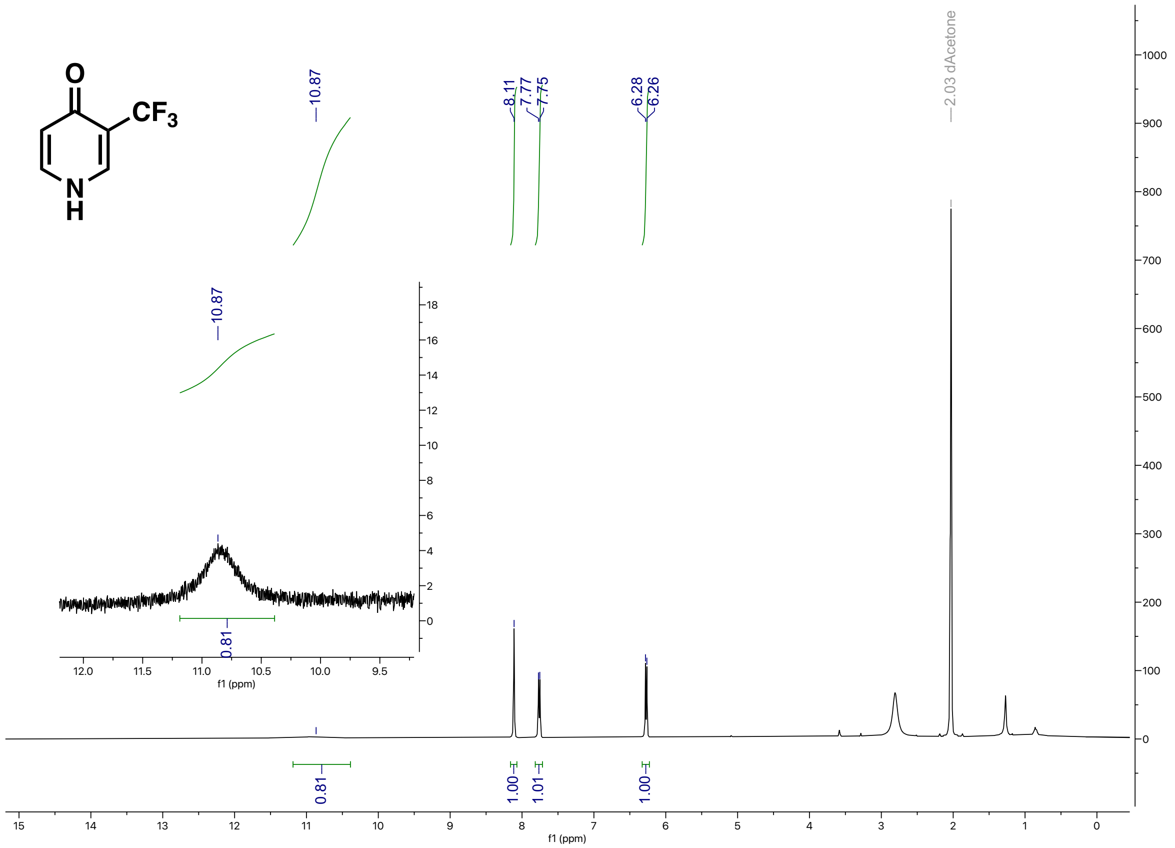

3j -^{19}F NMR (376 MHz, $CO(CD_3)_2$)

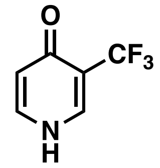

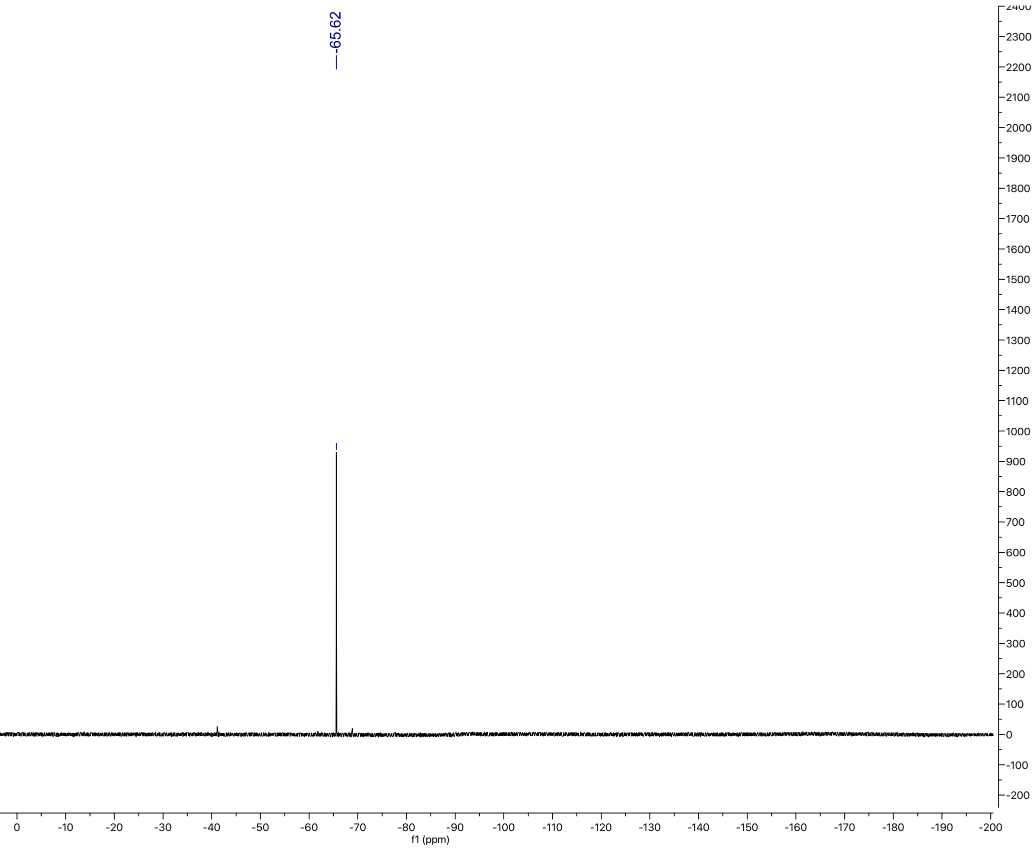

3j -^{13}C NMR (126 MHz, MeOD)

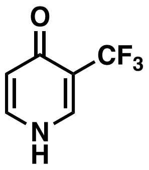

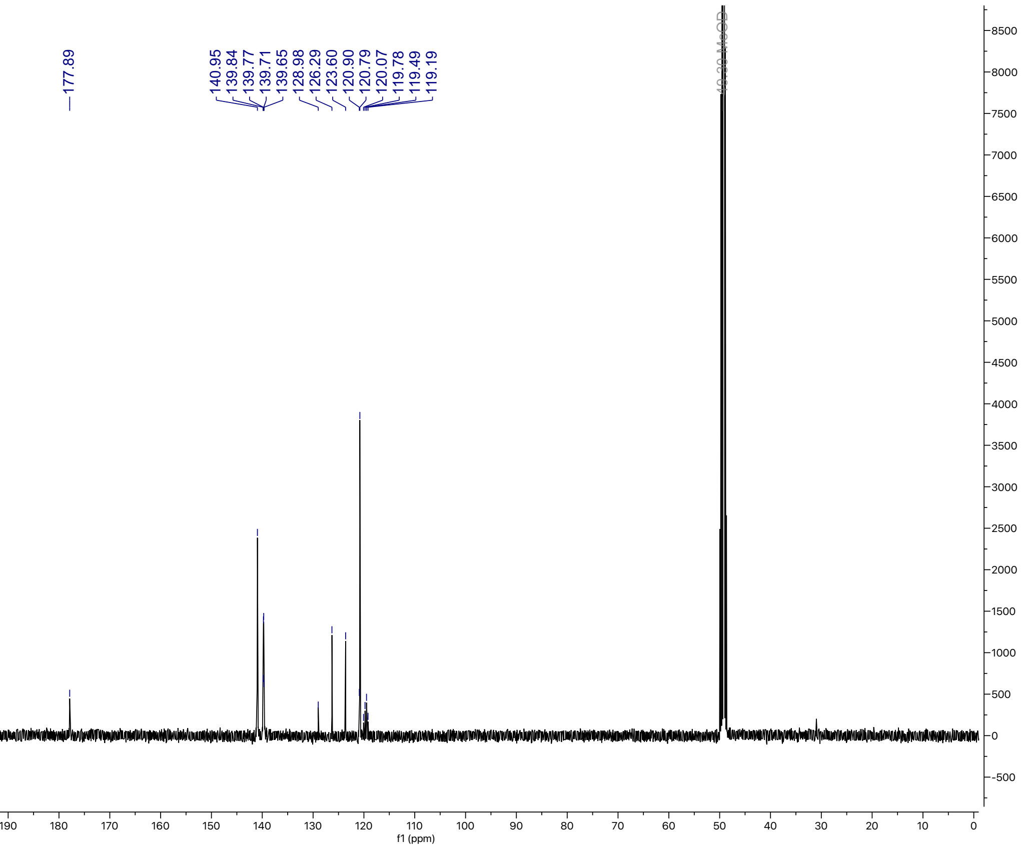

3j -HRMS (ESI)

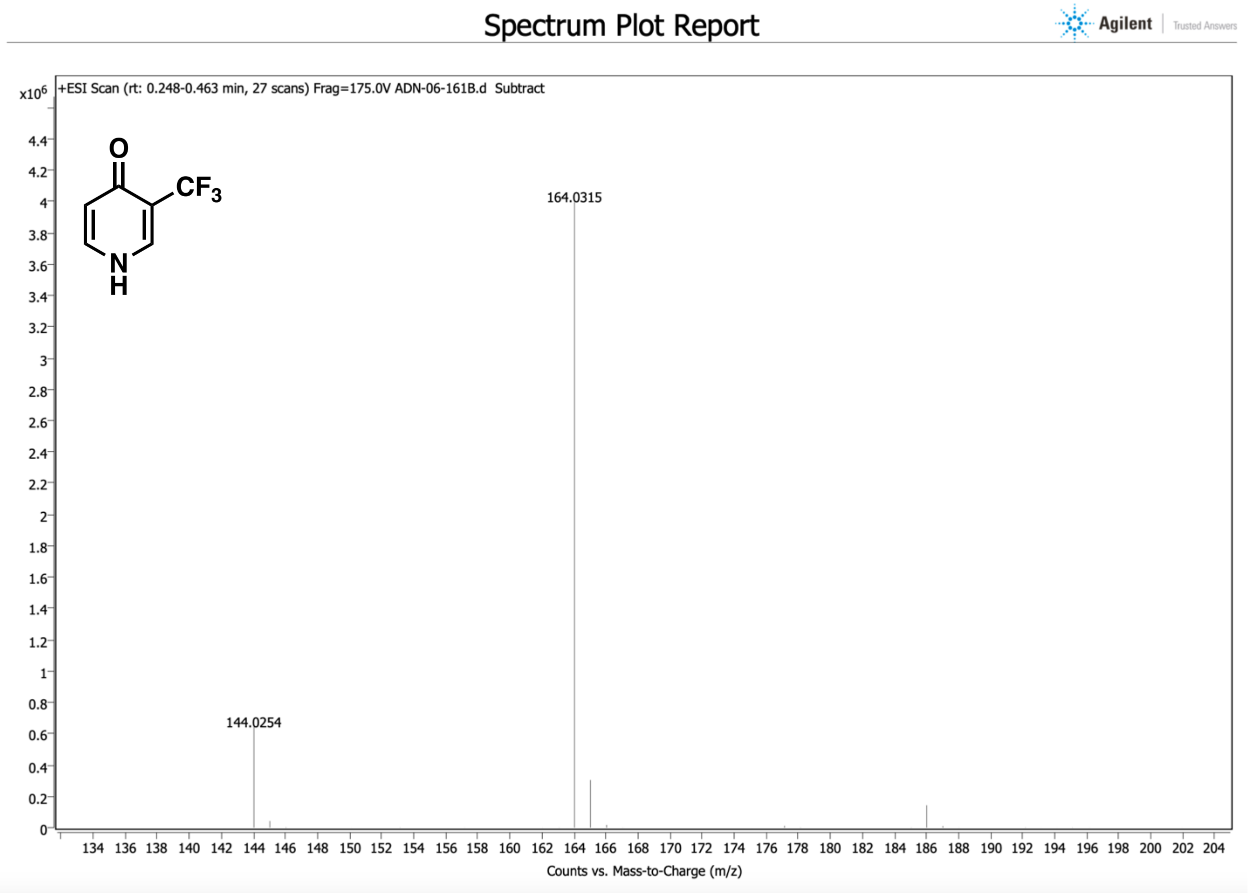
Spectrum Plot Report
Agilent
Trusted Answers
x10^6
+ESI Scan (rt: 0.248-0.463 min, 27 scans) Frag=175.0V ADN-06-161B.d Subtract
164.0315
144.0254
Counts vs. Mass-to-Charge (m/z)

(3k) 3,5-bis(trifluoromethyl)pyridin-4-ol

3k - ^{1}H NMR (400 MHz, MeOD)

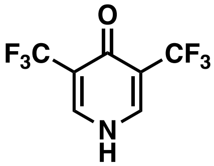

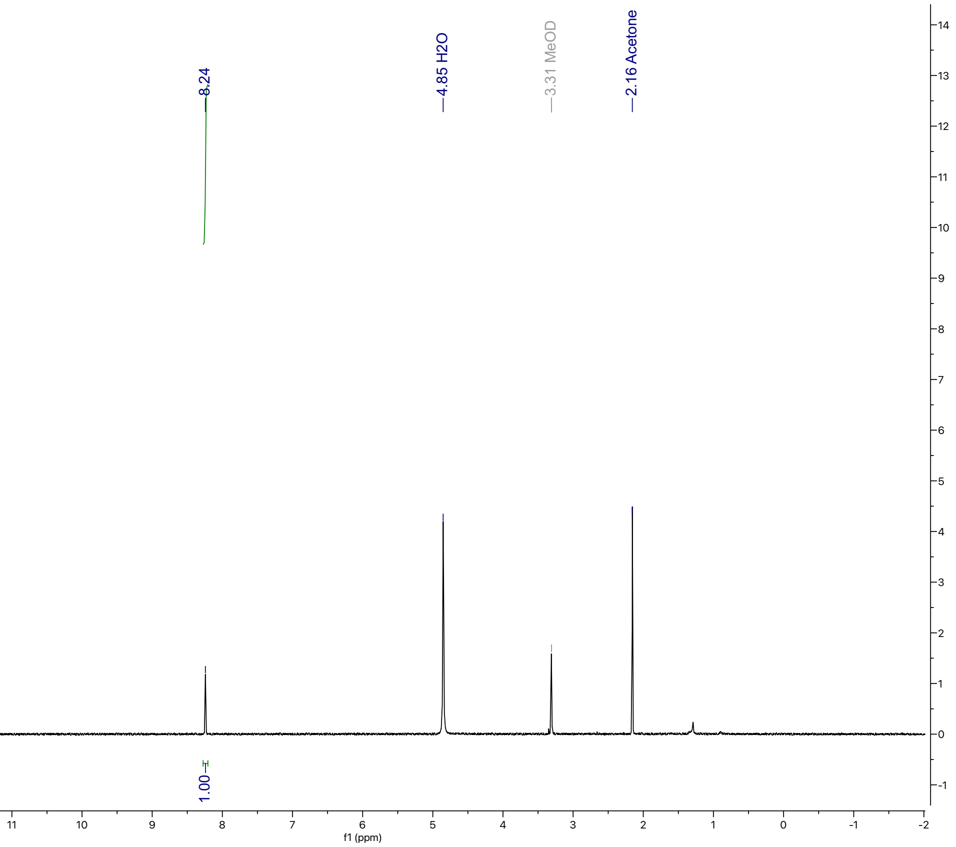

3k - ^{19}F NMR (376 MHz, MeOD)

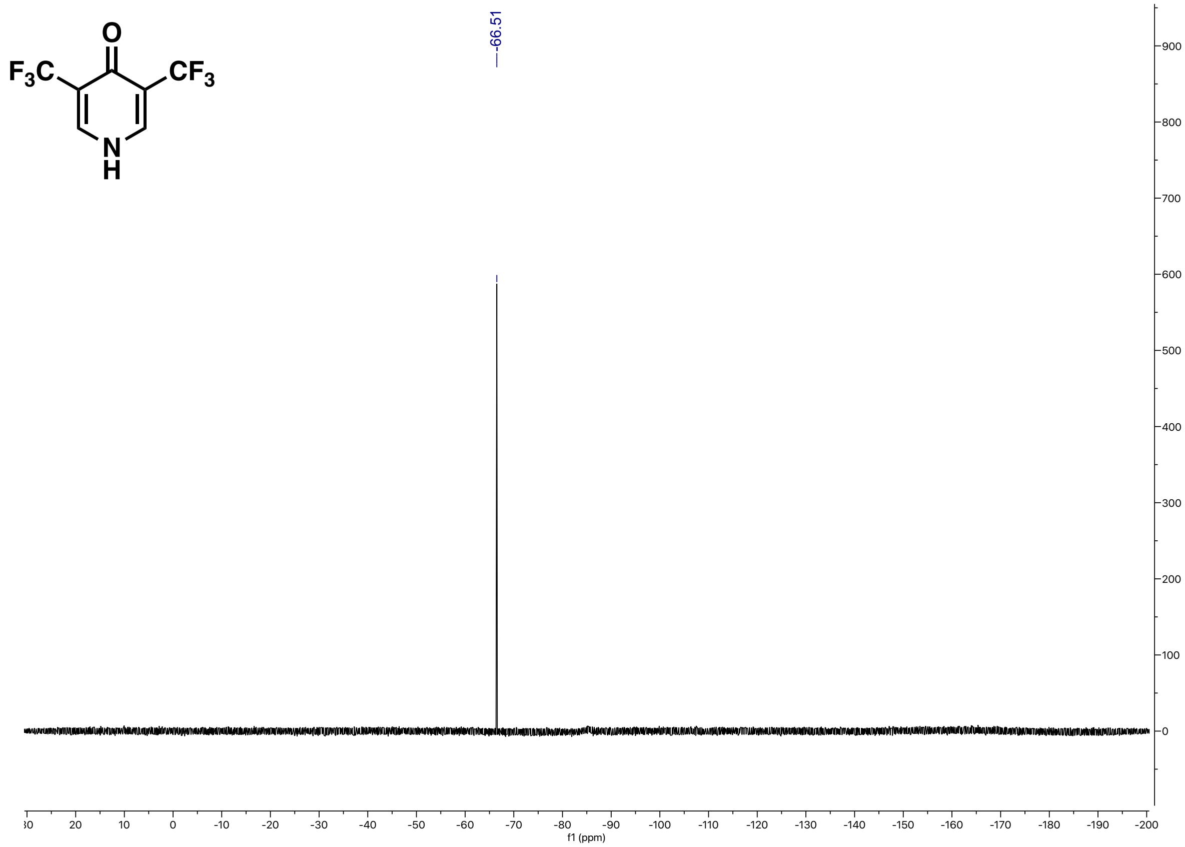

3k - ^{13}C NMR (126 MHz, MeOD)

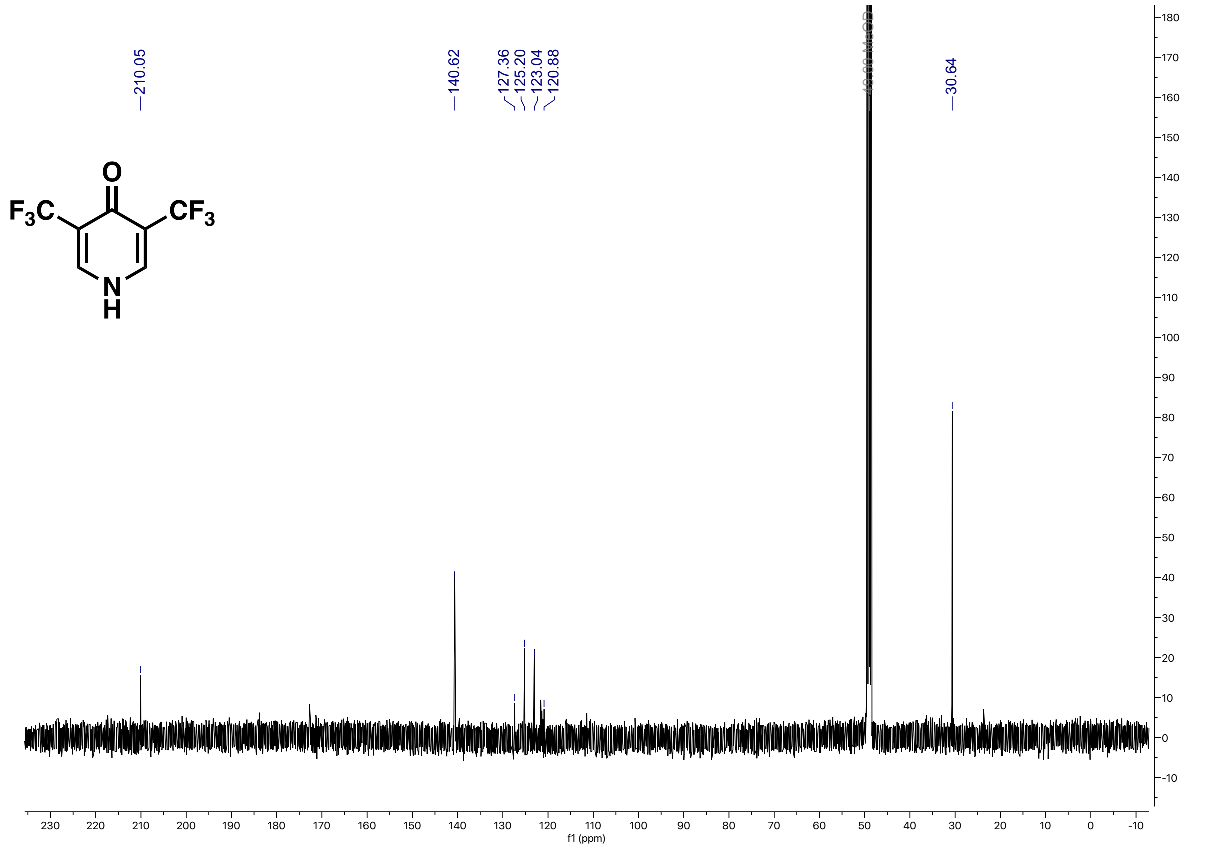

3k – HRMS (ESI)

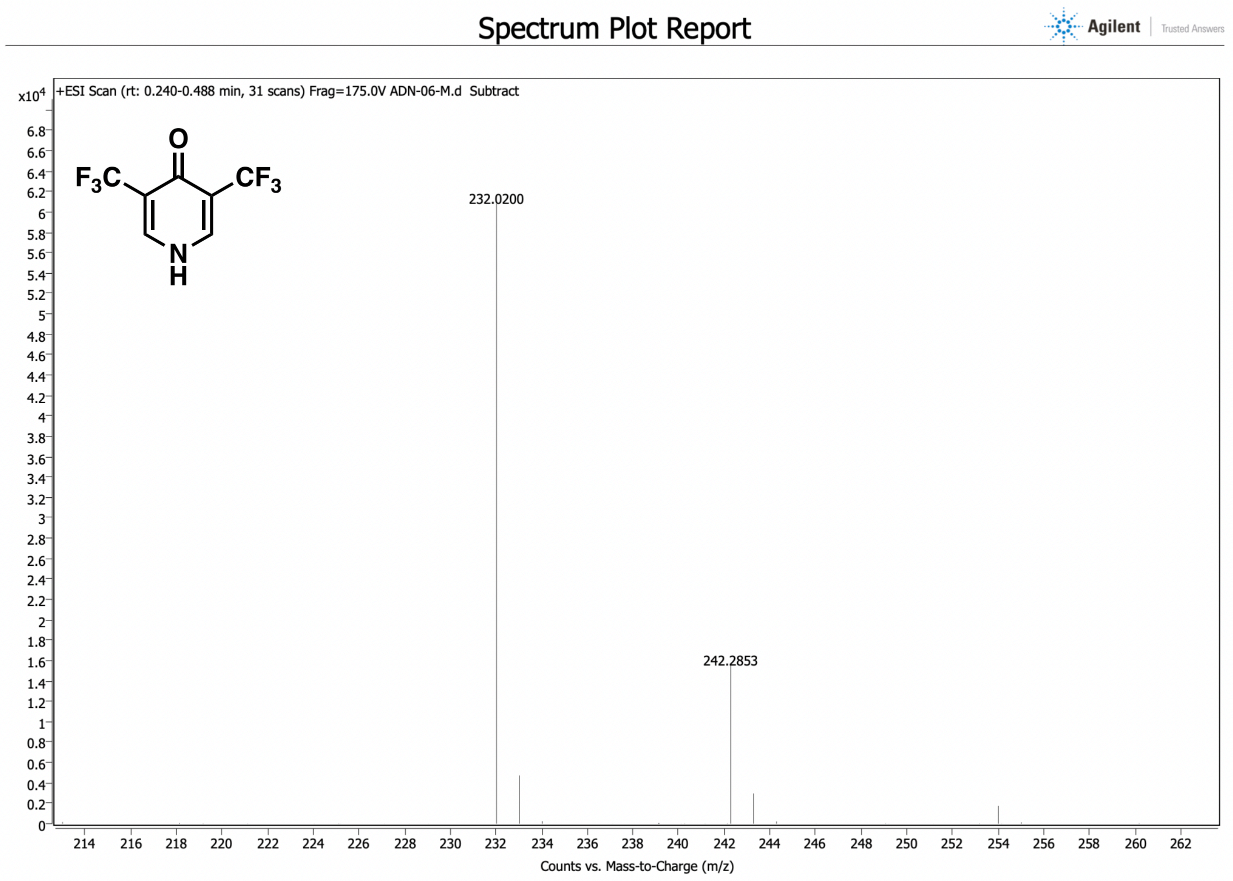

Spectrum Plot Report
Agilent
Trusted Answers
x10^4
+ESI Scan (rt: 0.240-0.488 min, 31 scans) Frag=175.0V ADN-06-M.d Subtract
O
F3C
CF3
N
H
232.0200
242.2853
Counts vs. Mass-to-Charge (m/z)

(3l) 5-(trifluoromethyl)pyrimidine-2,4(1H,3H)-dione

This compound is also described in *Tetrahedron.* **1982**, *23*, 4099-4100 and *Chem. Comm*. **2018**, *54*, 13662-13665

3l - ^1H NMR (400 MHz, MeOD)

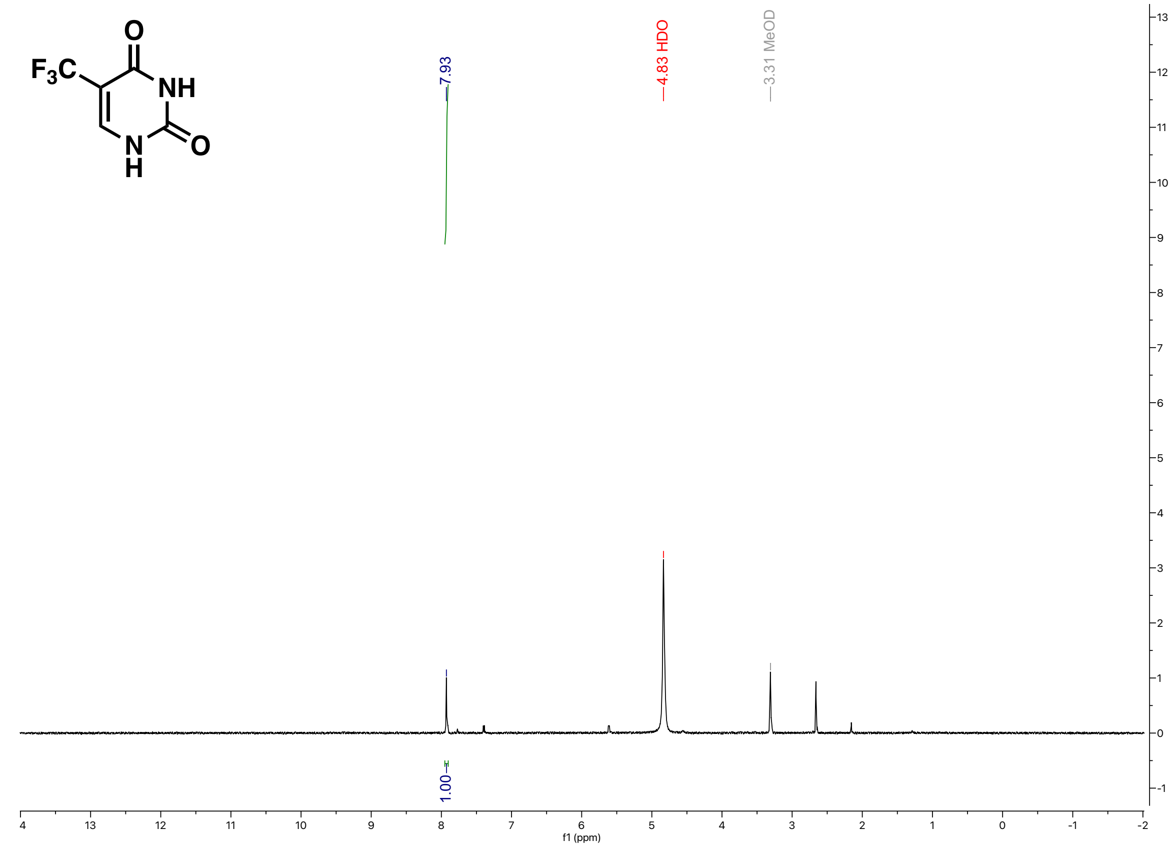

3l - ^{19}F NMR (376 MHz, MeOD)

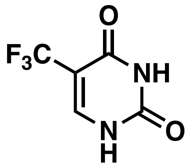

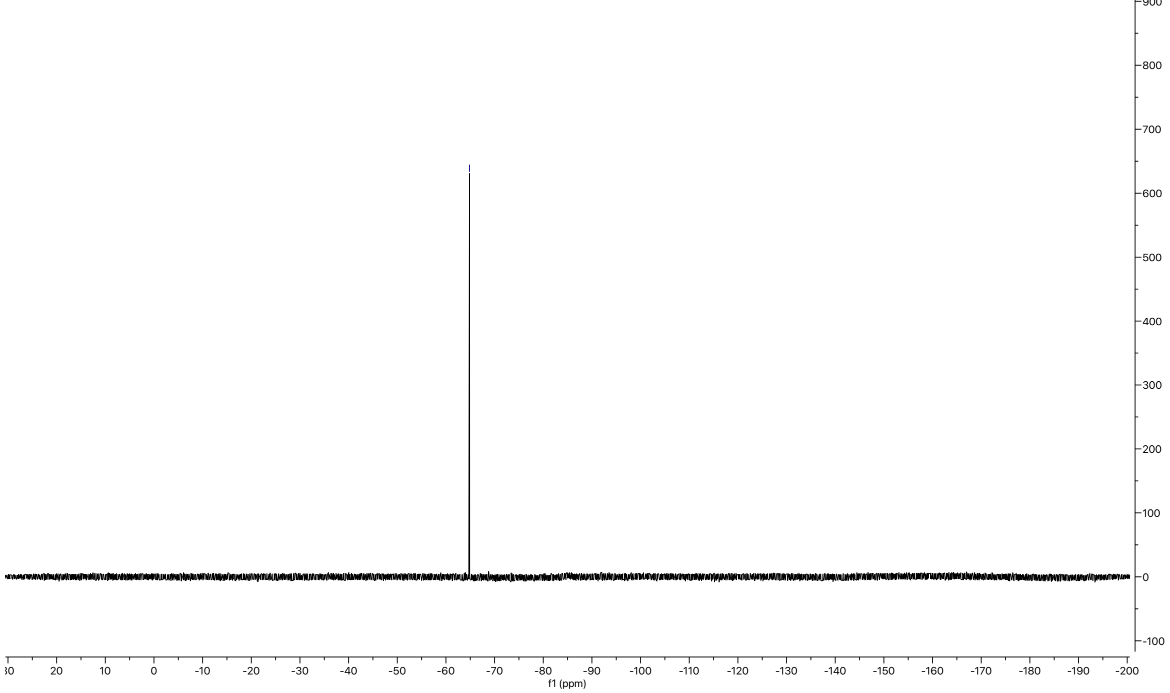

3l – HRMS (ESI)

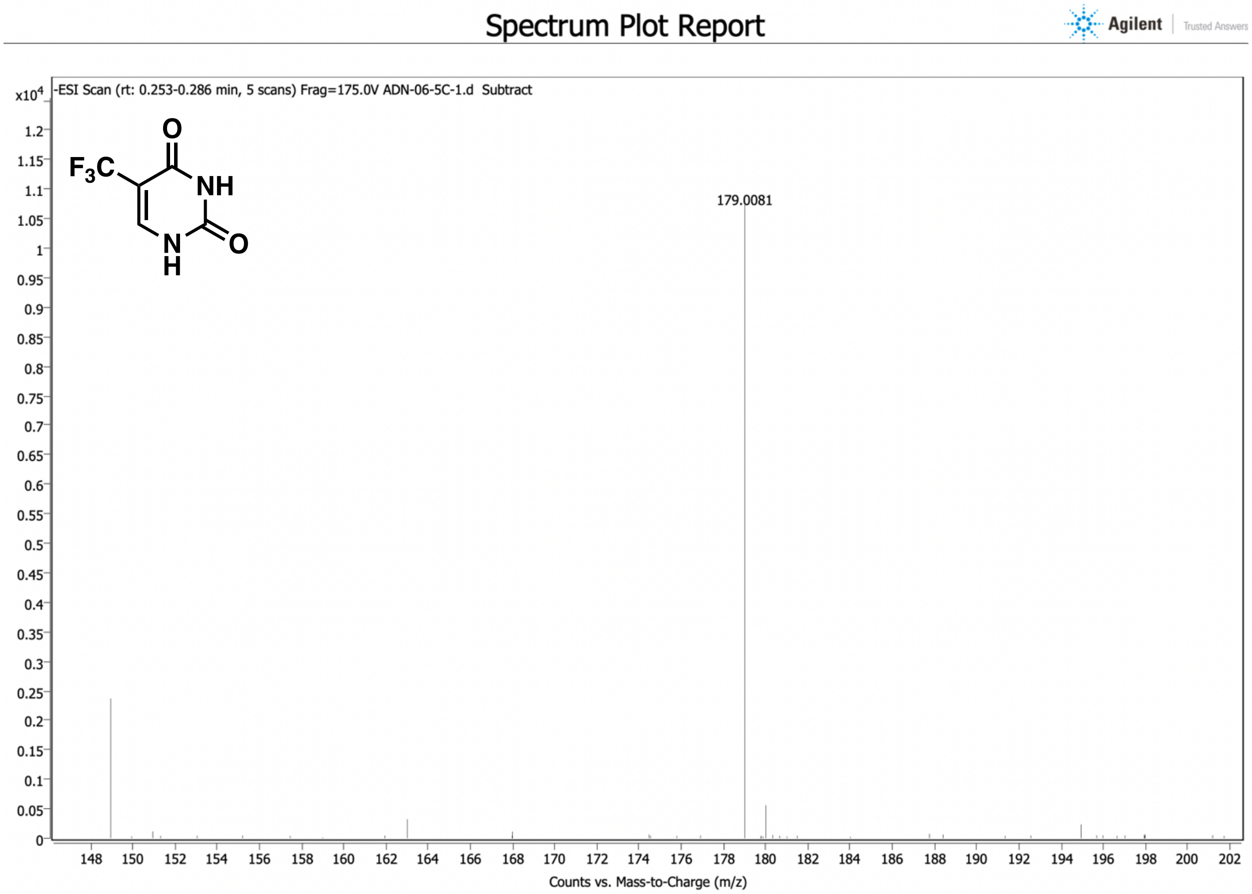

Spectrum Plot Report
Agilent
Trusted Answers
x10^4
-ESI Scan (rt: 0.253-0.286 min, 5 scans) Frag=175.0V ADN-06-5C-1.d Subtract
F3C
NH
N
H
O
O
179.0081
Counts vs. Mass-to-Charge (m/z)

(3m_1) 3-(trifluoromethyl)-1H-pyrrolo[2,3-b]pyridine

This compound is also described in *J. Am. Chem. Soc.* **2019**, *141*, 12872-12879

3m_1 - ^{1}H NMR (400 MHz, $CDCl_3$)

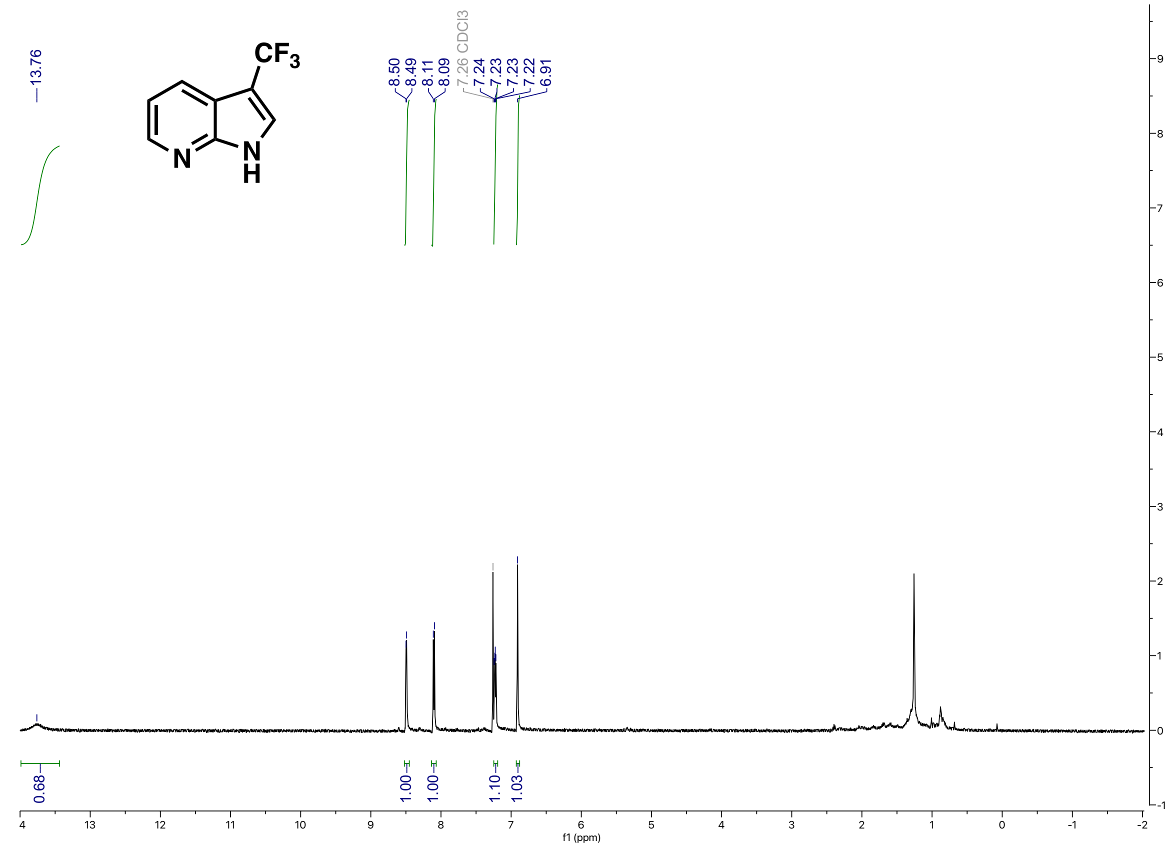

3m_1 - ^{19}F NMR (376 MHz, $CDCl_3$)

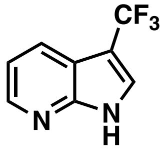

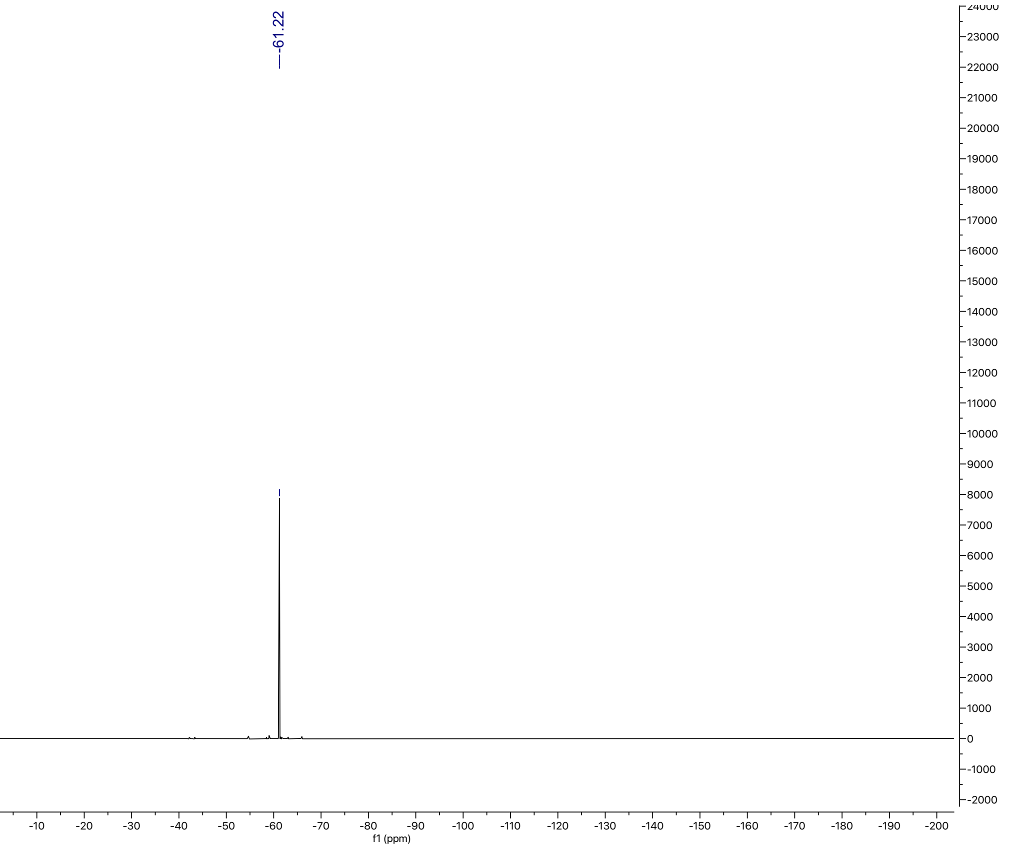

(3m_2) 2-(trifluoromethyl)-1H-pyrrolo[2,3-b]pyridine

This compound is also described in *J. Am. Chem. Soc.* **2019**, *141*, 12872-12879

3m_2 - ^{1}H NMR (400 MHz, $CDCl_3$)

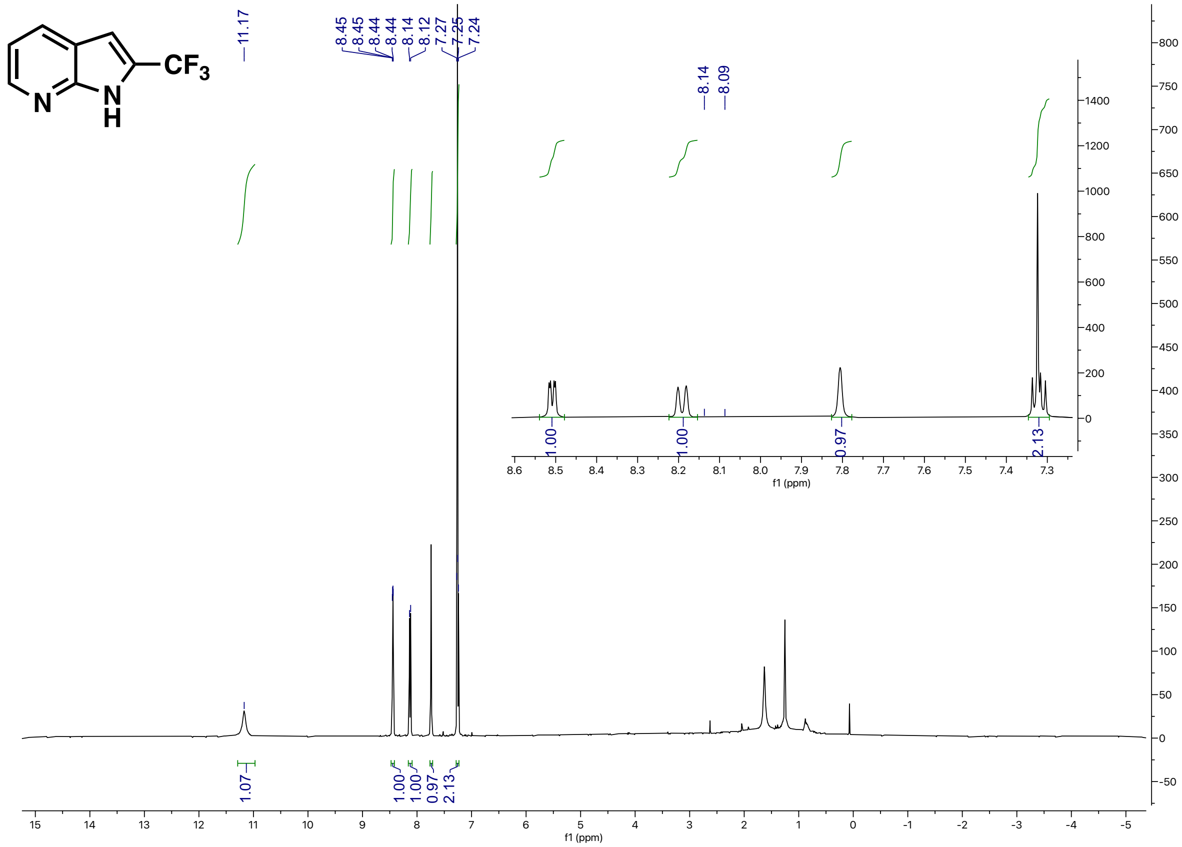

3m_2 - ^{19}F NMR (376 MHz, $CDCl_3$)

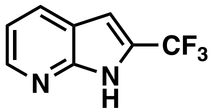

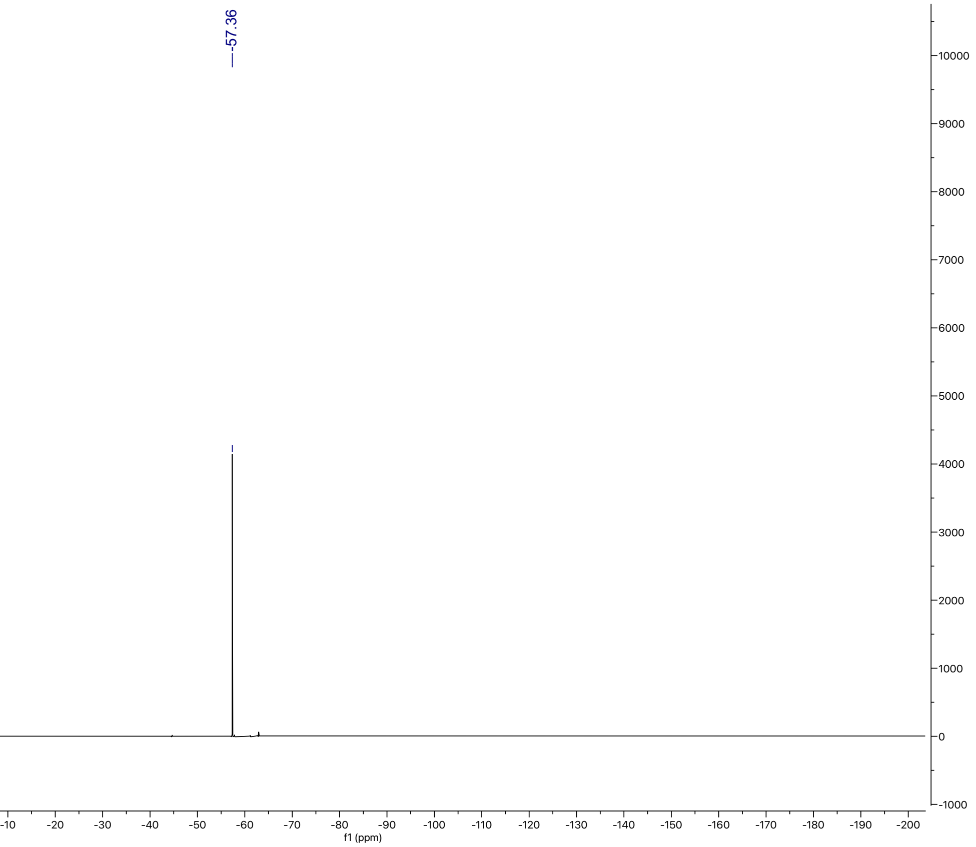

3m - HRMS, submitted as mixture of both isomers prior to separation

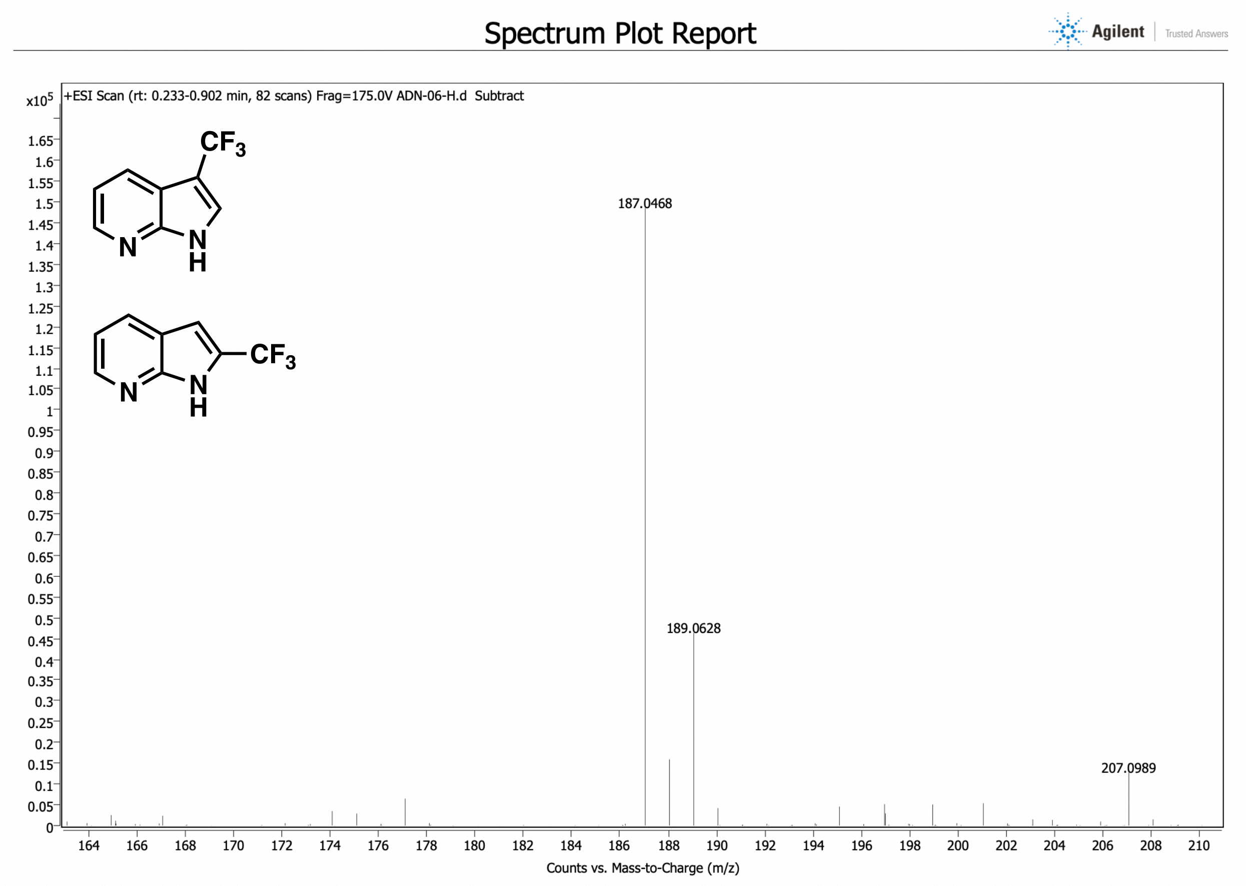

(3n) 3-(trifluoromethyl)quinoxalin-2(1H)-one

This compound is also described in *Org. Chem. Front.* **2019**, *6*, 2392-2397

3n - ^{1}H NMR (400 MHz, $CDCl_3$)

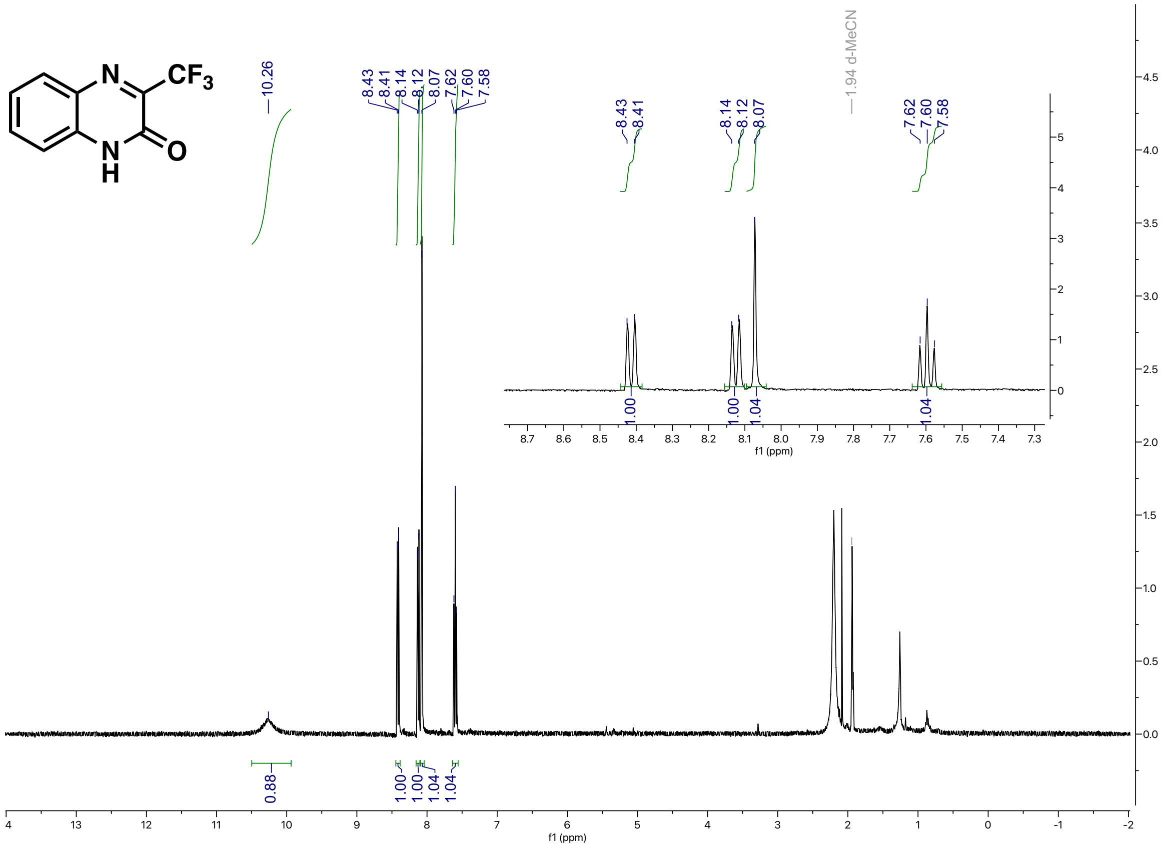

3n - ^{19}F NMR (376 MHz, $CDCl_3$)

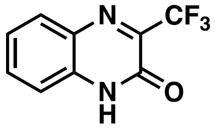

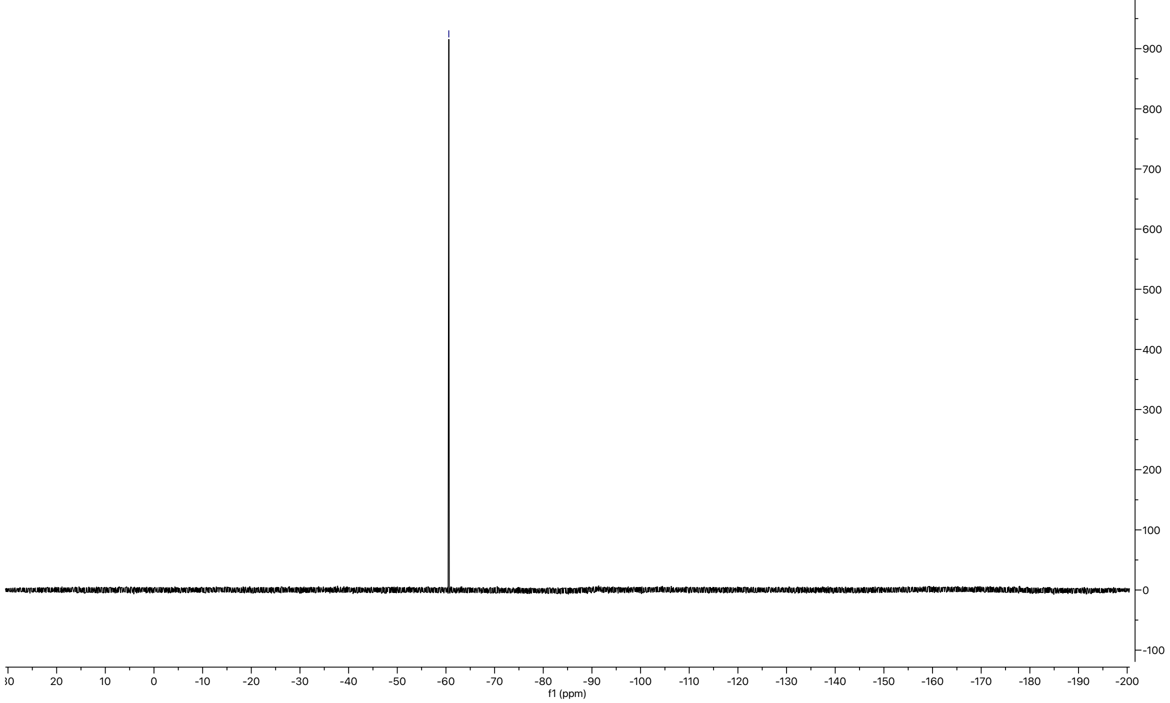

3n – HRMS (ESI)

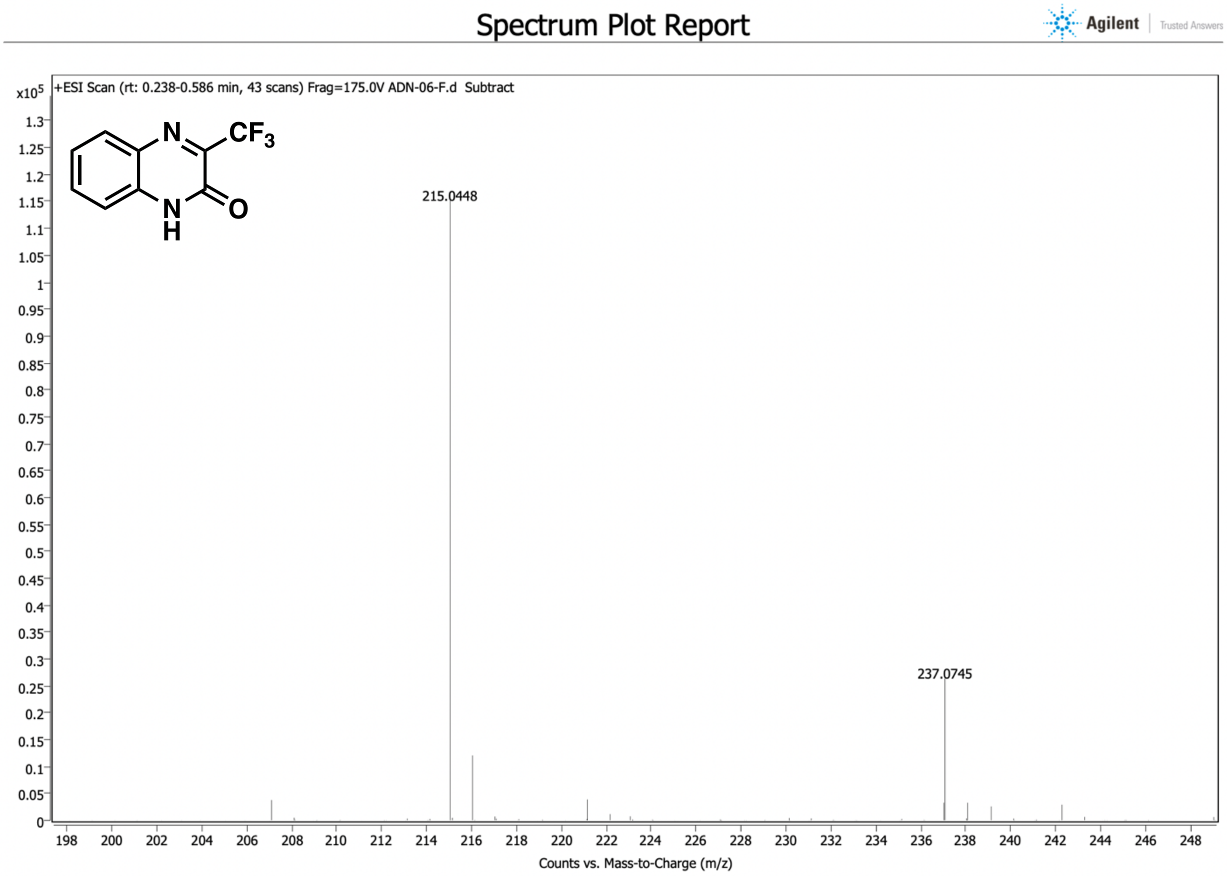

Spectrum Plot Report
Agilent
Trusted Answers
x10^5
+ESI Scan (rt: 0.238-0.586 min, 43 scans) Frag=175.0V ADN-06-F.d Subtract
N
CF3
N
H
O
215.0448
237.0745
1.3
1.25
1.2
1.15
1.1
1.05
1
0.95
0.9
0.85
0.8
0.75
0.7
0.65
0.6
0.55
0.5
0.45
0.4
0.35
0.3
0.25
0.2
0.15
0.1
0.05
0
198 200 202 204 206 208 210 212 214 216 218 220 222 224 226 228 230 232 234 236 238 240 242 244 246 248
Counts vs. Mass-to-Charge (m/z)

(3o) 6-(quinolin-2-yl)-3-(trifluoromethyl)pyridin-2(1H)-one

1o – ^{1}H NMR (400MHz, CDCl3), synthesized using previously published methods (see S16)

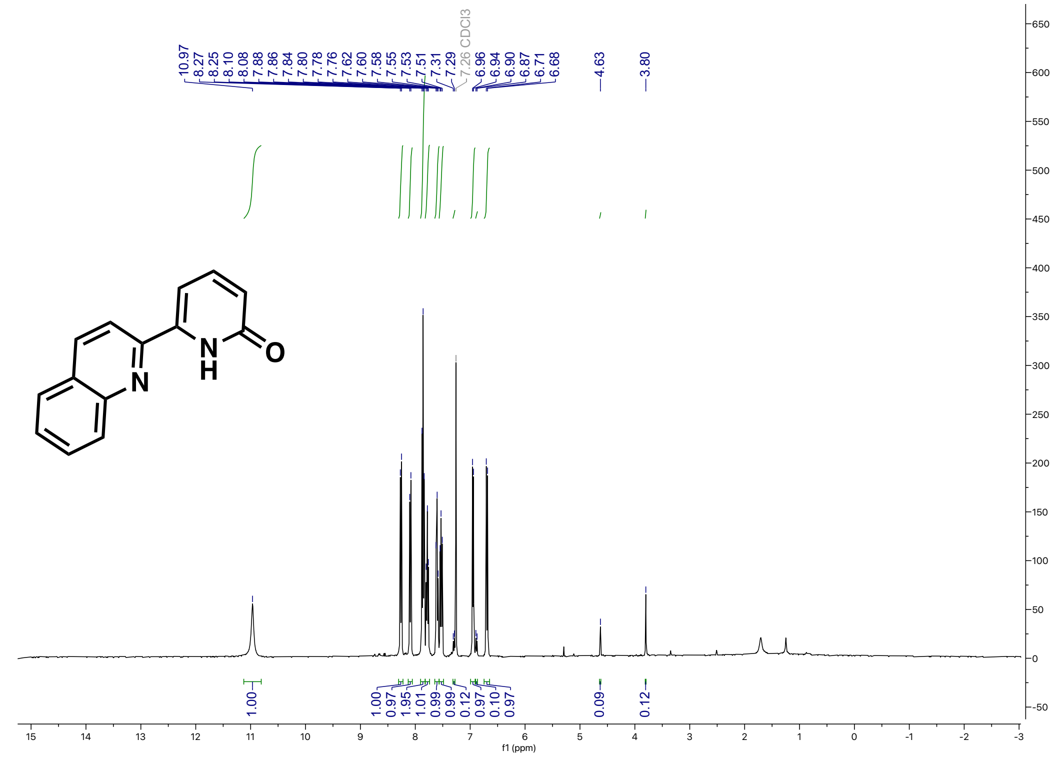

3o - ^{1}H NMR (400 MHz, $CDCl_3$)

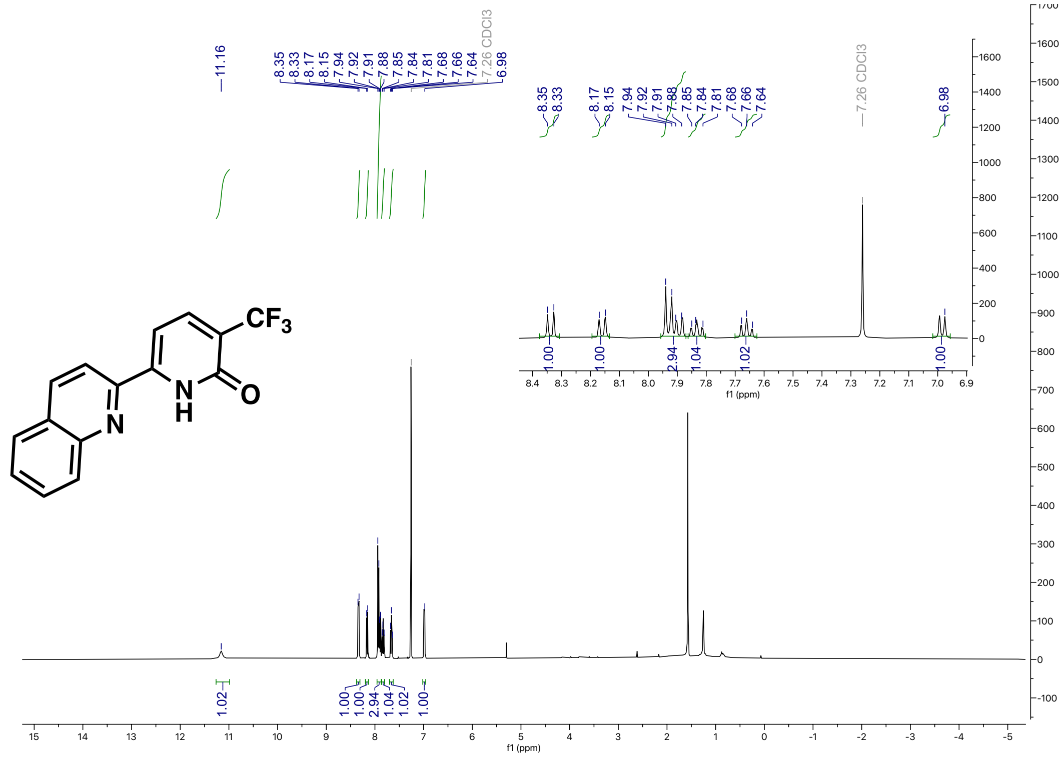

3o - ^{19}F NMR (376 MHz, $CDCl_3$)

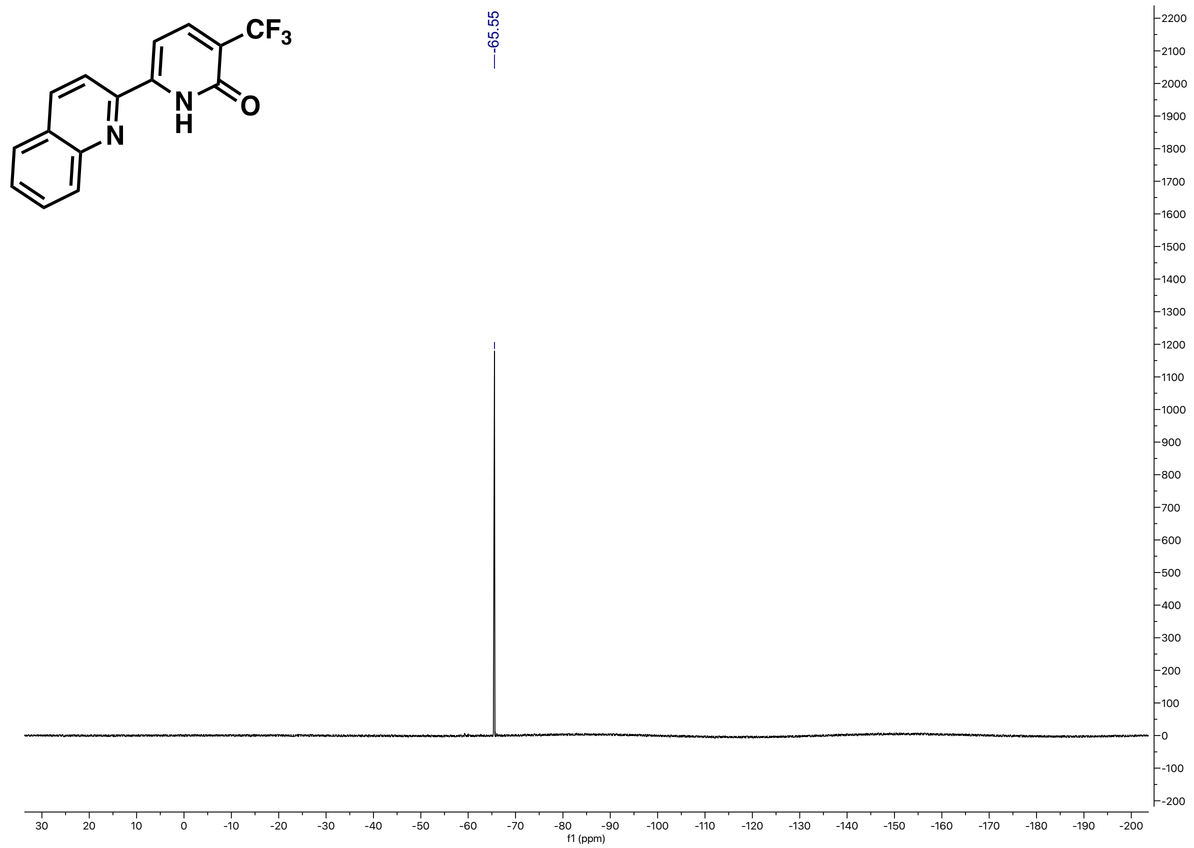

3o - ^{13}C NMR (126 MHz, $CDCl_3$)

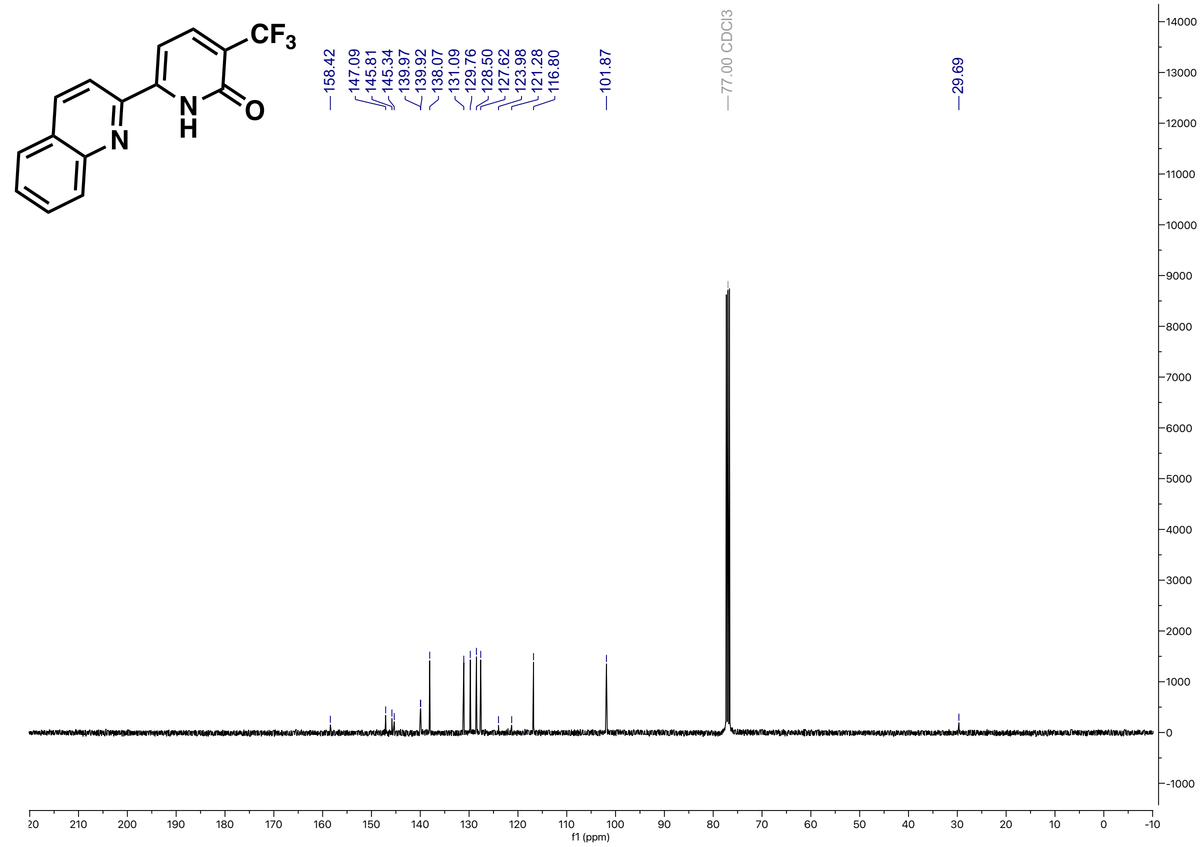

3o – HRMS (ESI)

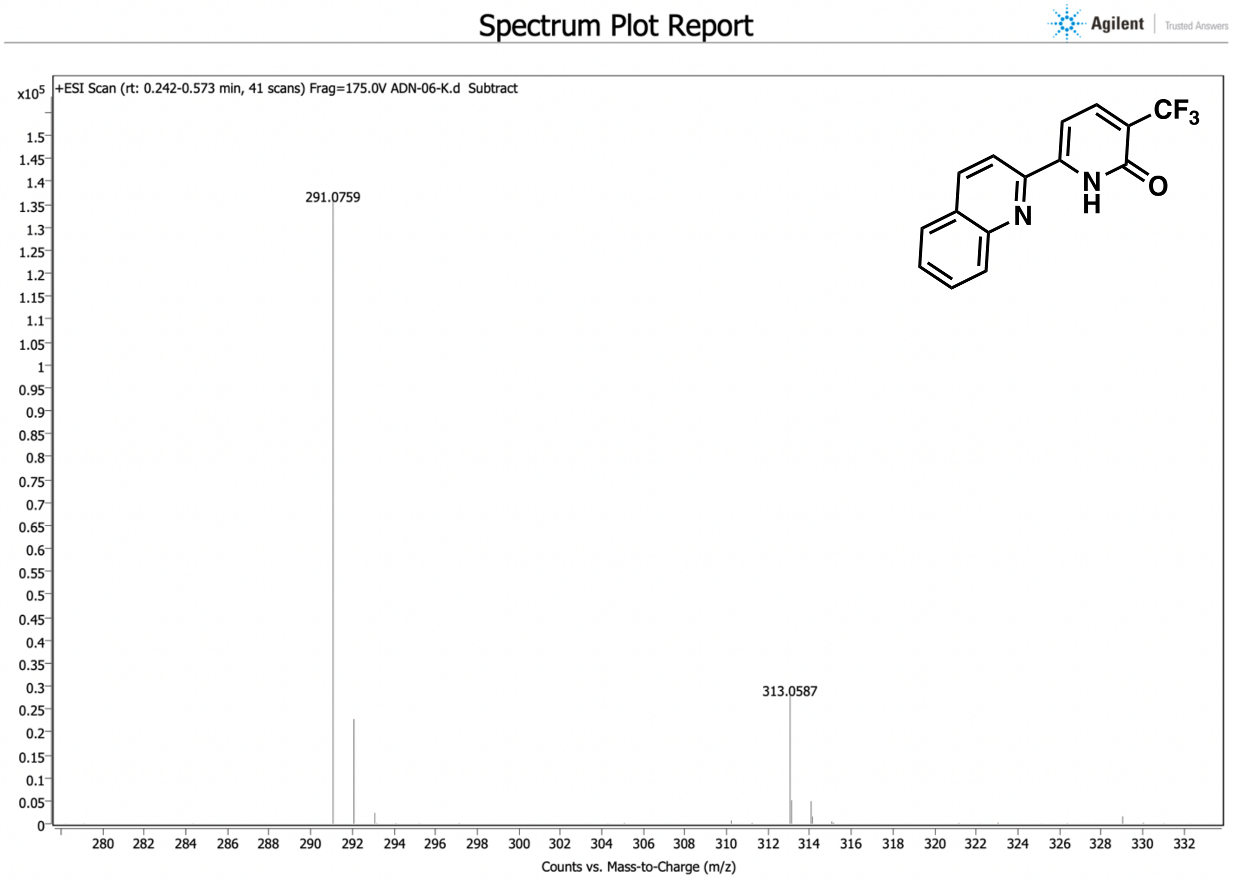

Spectrum Plot Report
Agilent
Trusted Answers
x10^5
+ESI Scan (rt: 0.242-0.573 min, 41 scans) Frag=175.0V ADN-06-K.d Subtract
291.0759
313.0587
CF3
N
H
O
N
Counts vs. Mass-to-Charge (m/z)

(3p) 5-(trifluoromethyl)-[2,2'-bipyridin]-6(1*H*)-one

1p – 1H NMR (400MHz, $CDCl_3$), synthesized by previously published methods (see S17)

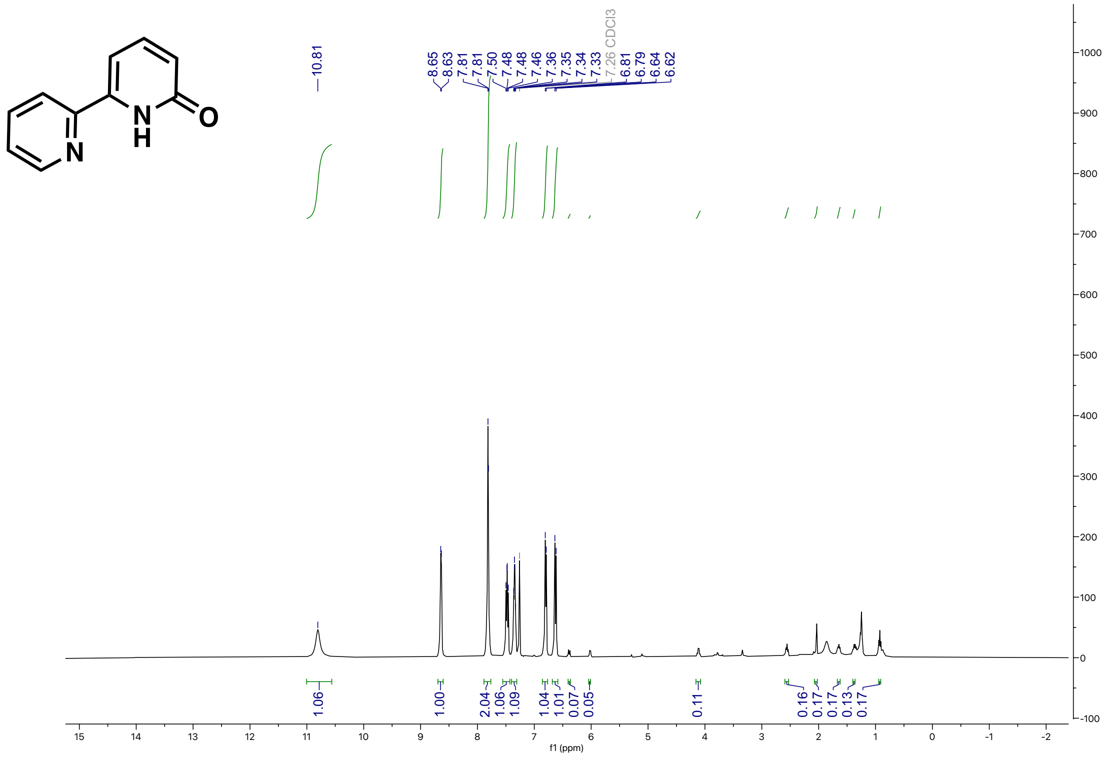

3p – ^{1}H NMR (400 MHz, CD_2Cl_2)

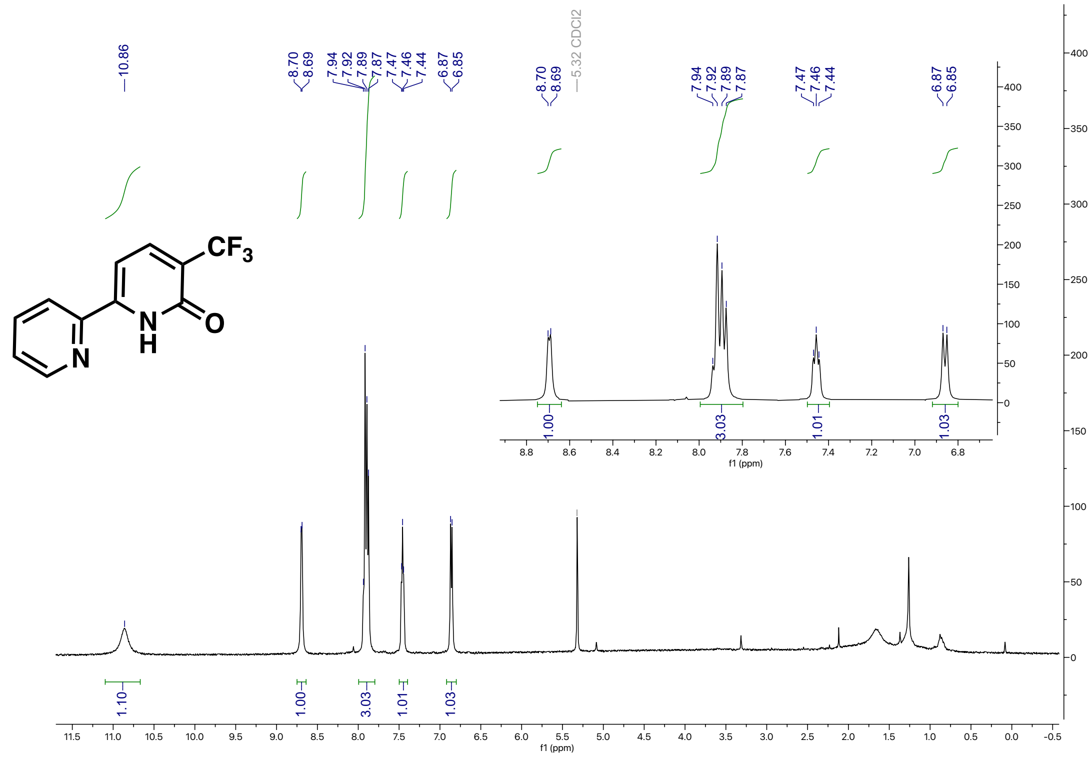

3p – ^{19}F NMR (376 MHz, CD_2Cl_2)

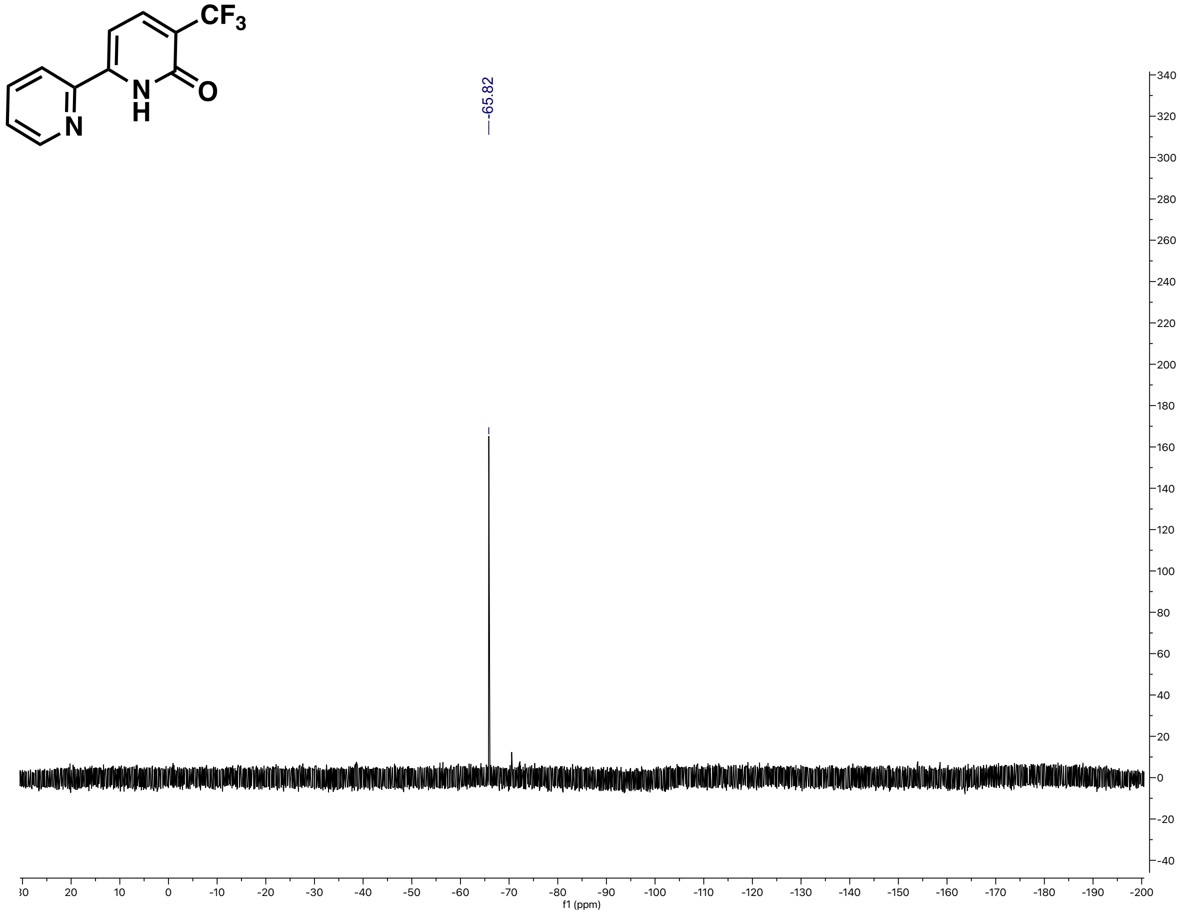

3p – ^{13}C NMR (126 MHz, CD_3OD)

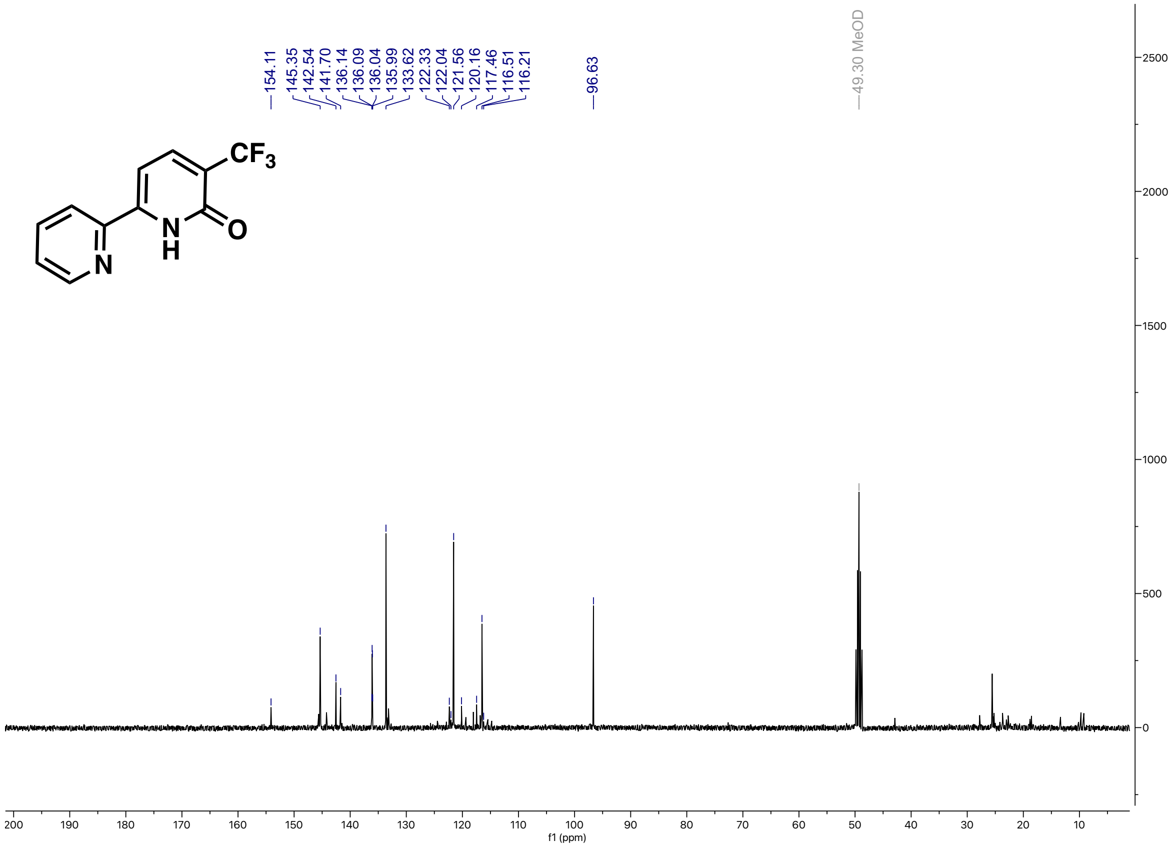

3p – HRMS (ESI)

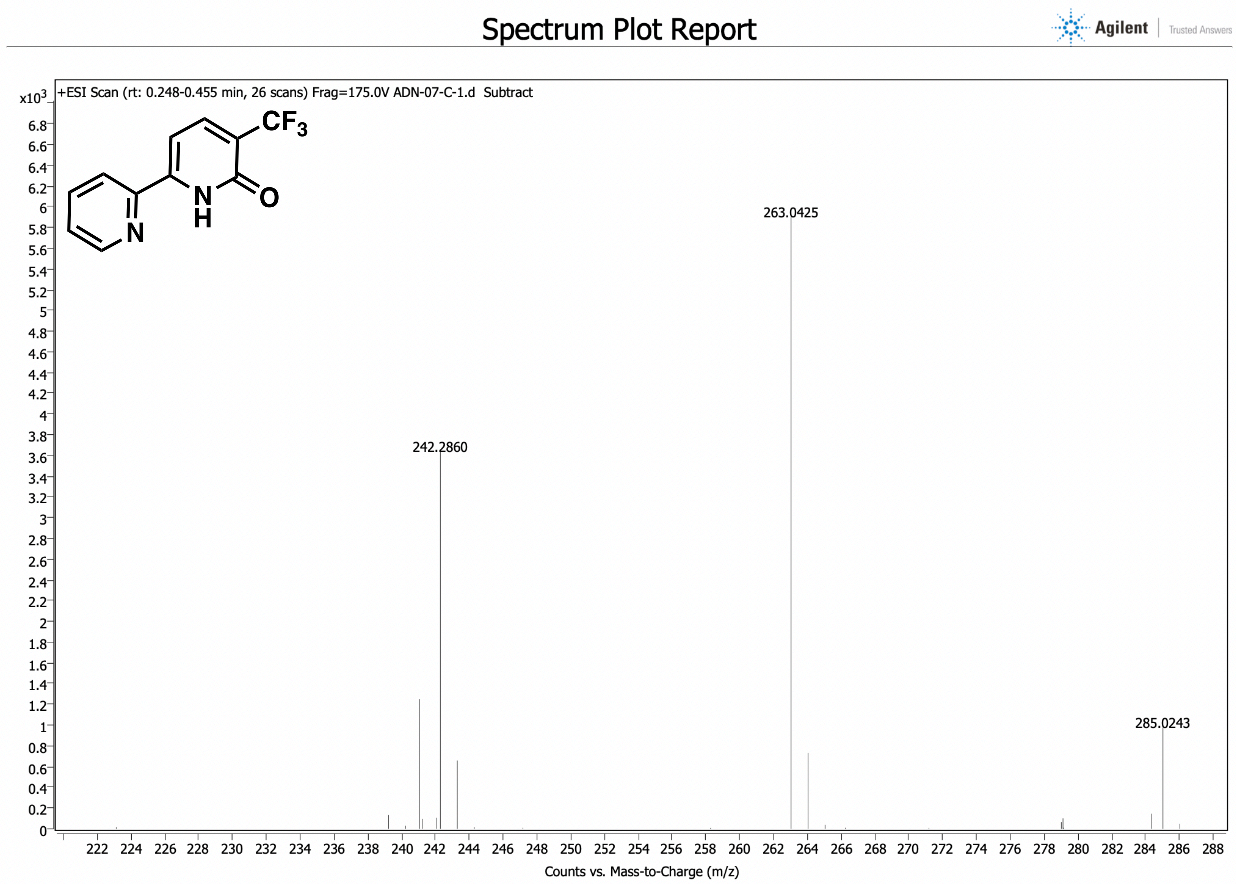
Spectrum Plot Report
Agilent
Trusted Answers
+ESI Scan (rt: 0.248-0.455 min, 26 scans) Frag=175.0V ADN-07-C-1.d Subtract
x10^3
CF3
N
H
O
N
263.0425
242.2860
285.0243
Counts vs. Mass-to-Charge (m/z)

(3s) [Pirfenidone-CF3]; 5-methyl-1-phenyl-3-(trifluoromethyl)pyridin-2(1H)-one

This compound is also described in *Chem. Commun*. **2018**, *54*, 10574-10577

3s – ^{1}H NMR (400 MHz, $CDCl_3$)

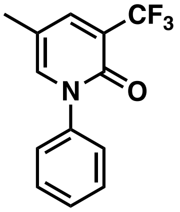

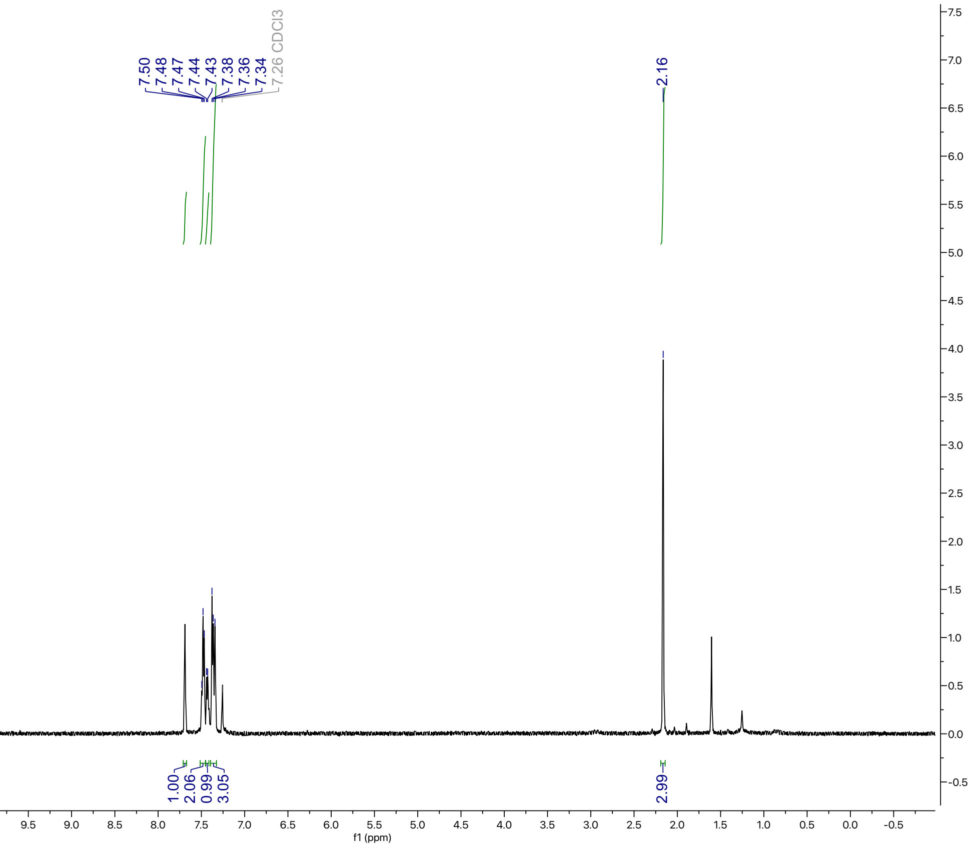

3s – ^{19}F NMR (376 MHz, $CDCl_3$)

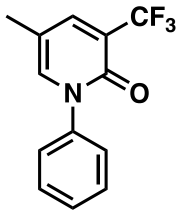

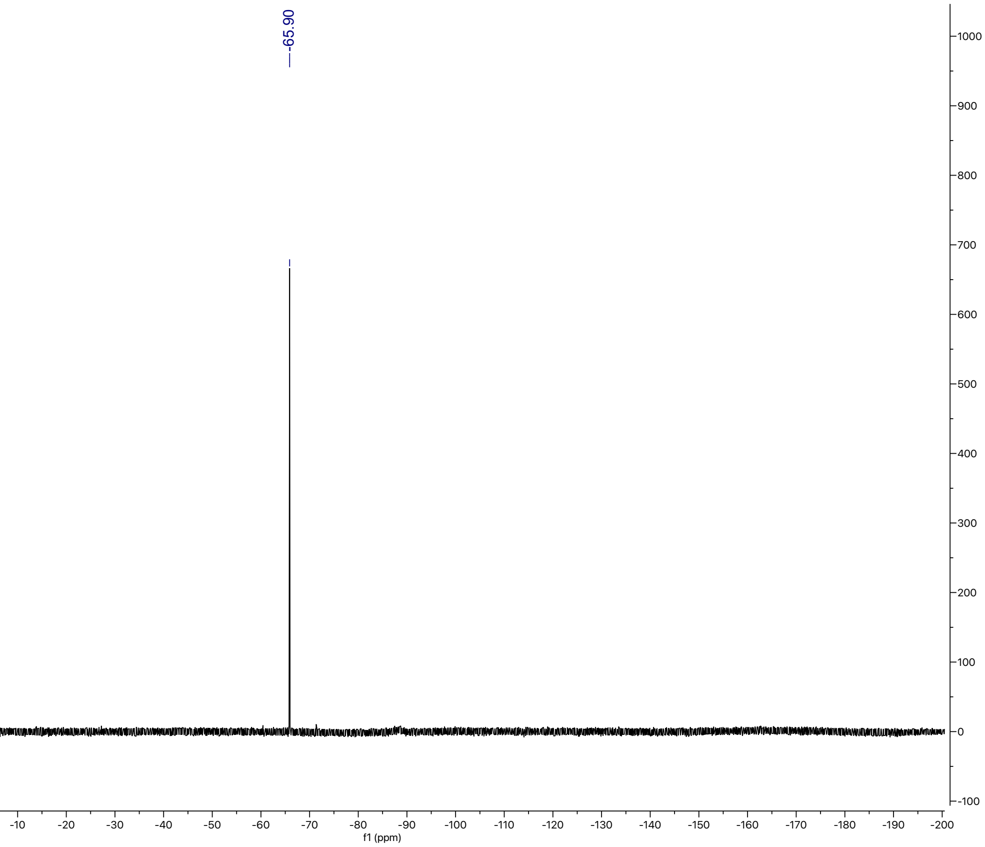

3s – ^{13}C NMR (126 MHz, $CDCl_3$)

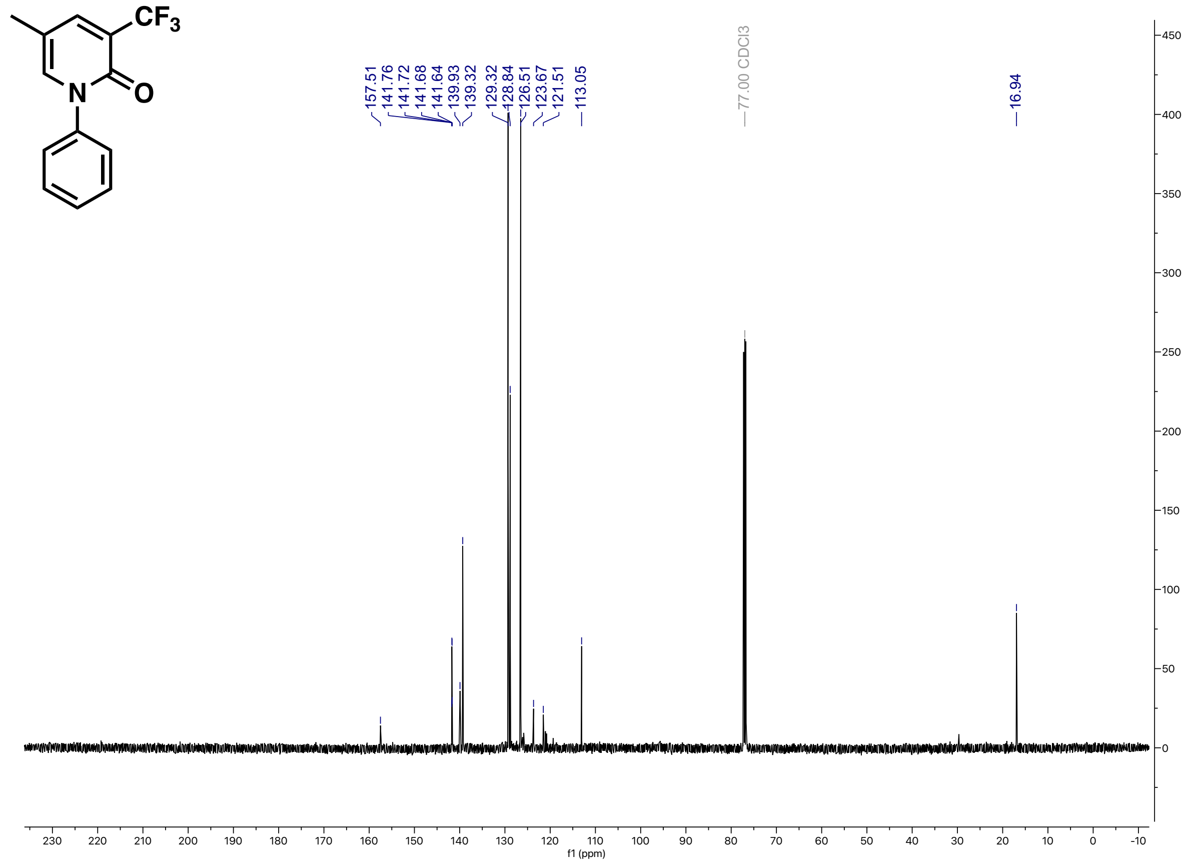

3s – HRMS (ESI)

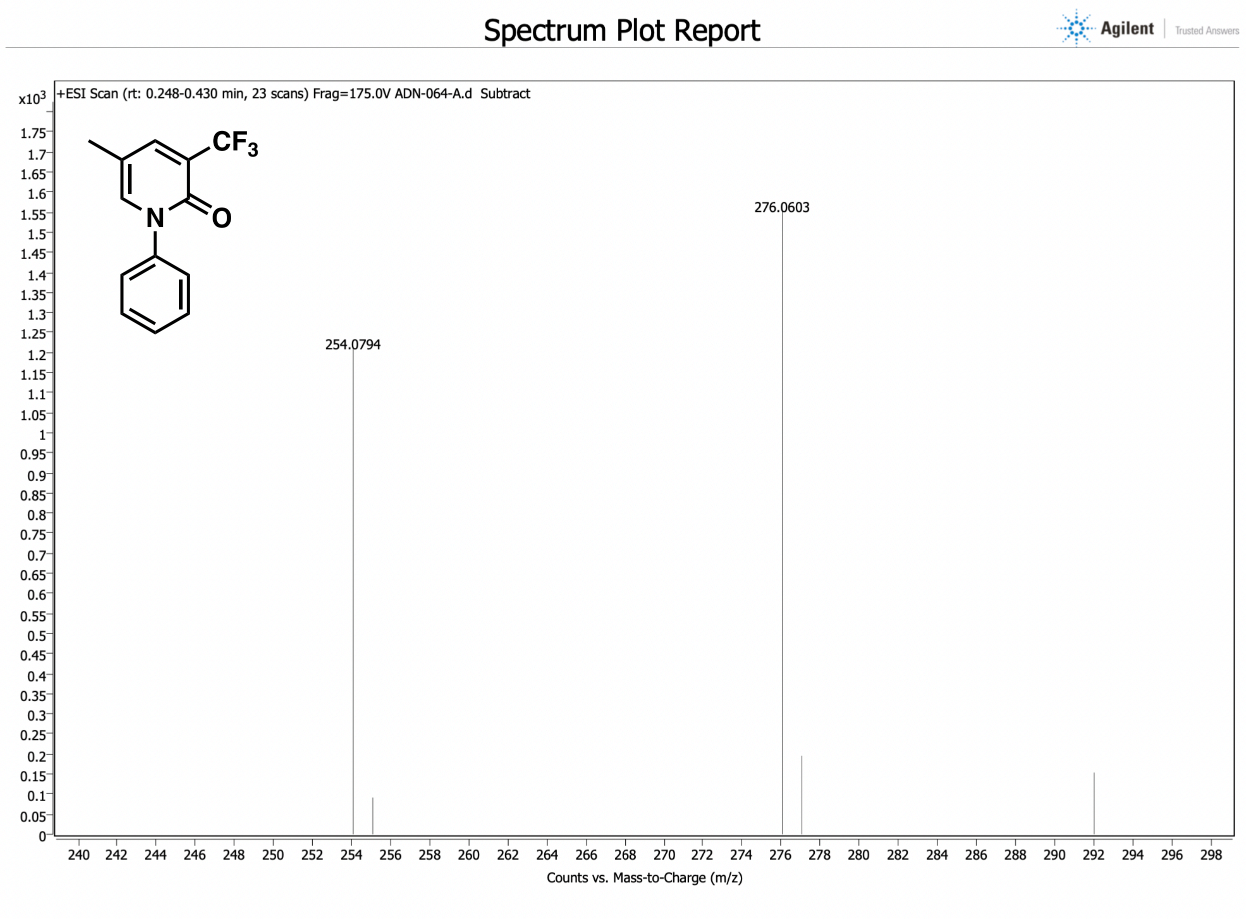

Spectrum Plot Report
Agilent
Trusted Answers
x10^3
+ESI Scan (rt: 0.248-0.430 min, 23 scans) Frag=175.0V ADN-064-A.d Subtract
CF3
N
O
276.0603
254.0794
Counts vs. Mass-to-Charge (m/z)

(3t) [Ciclopirox-CF3]; 6-cyclohexyl-4-methyl-3-(trifluoromethyl)pyridin-2(1H)-one

3t - ^{1}H NMR (400 MHz, $CDCl_3$)

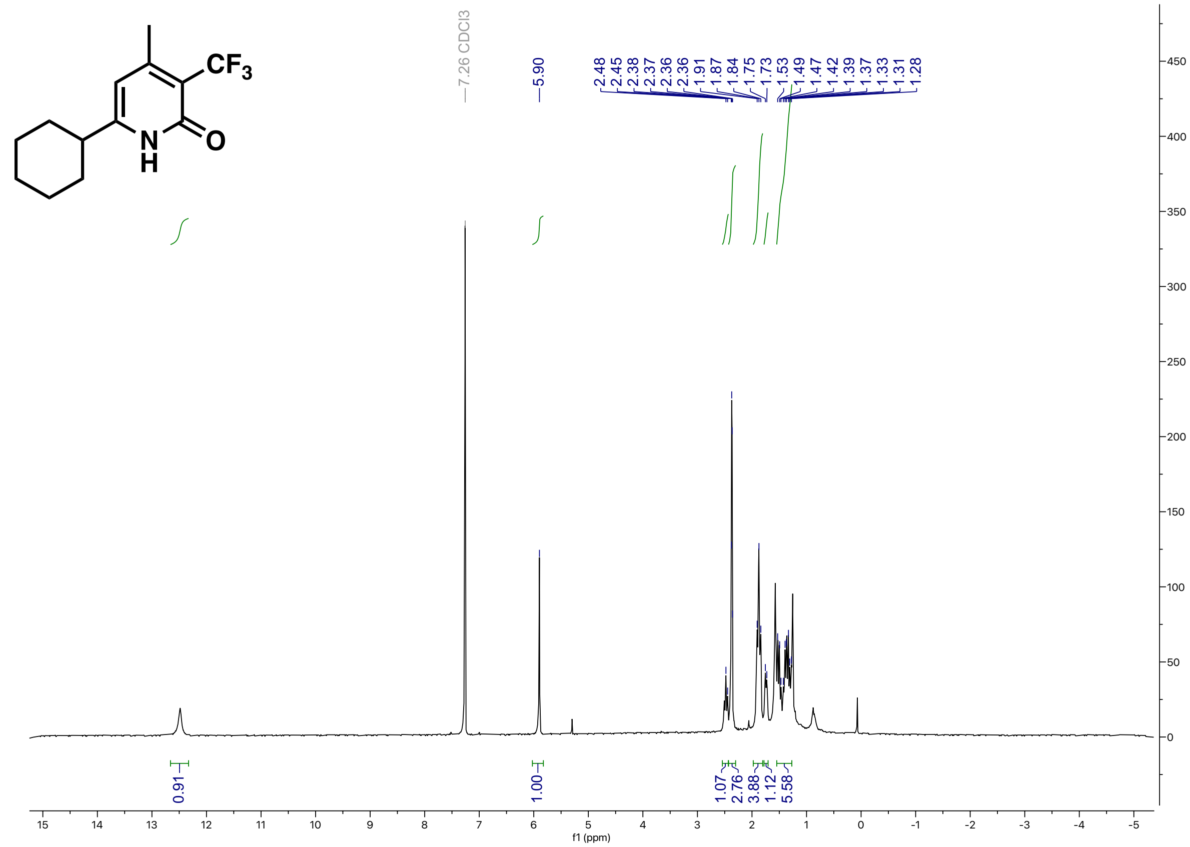

3t - ^{19}F NMR (376 MHz, $CDCl_3$)

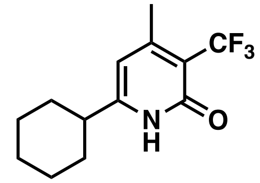

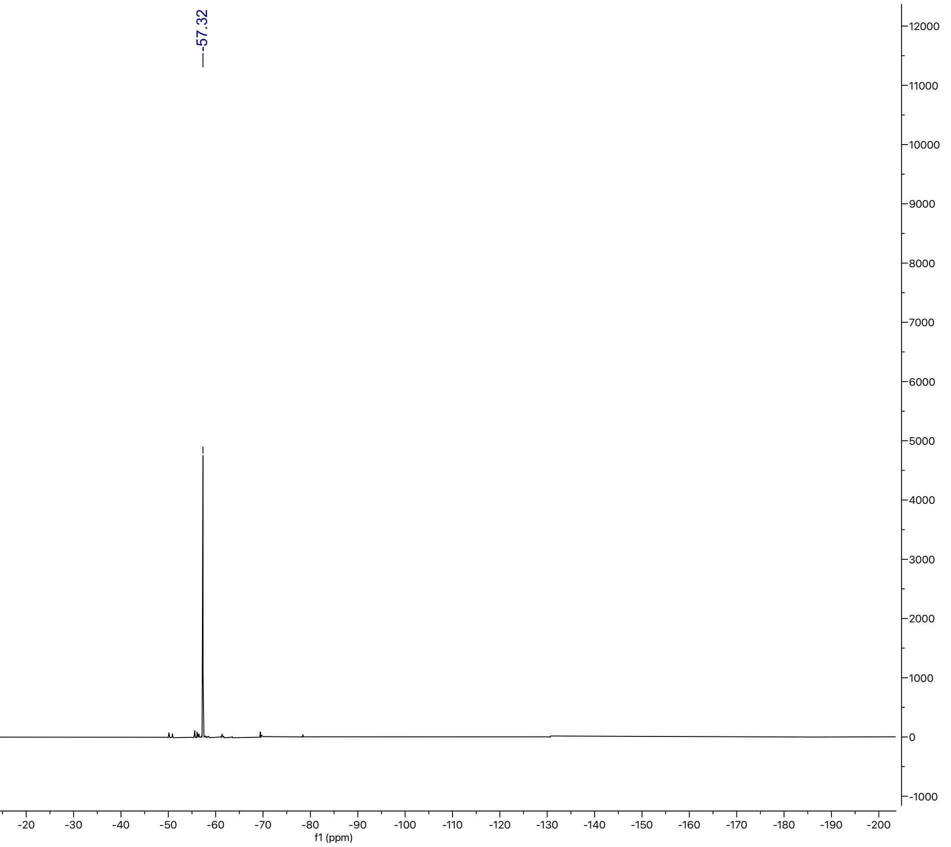

3t - ^{13}C NMR (126 MHz, $CDCl_3$)

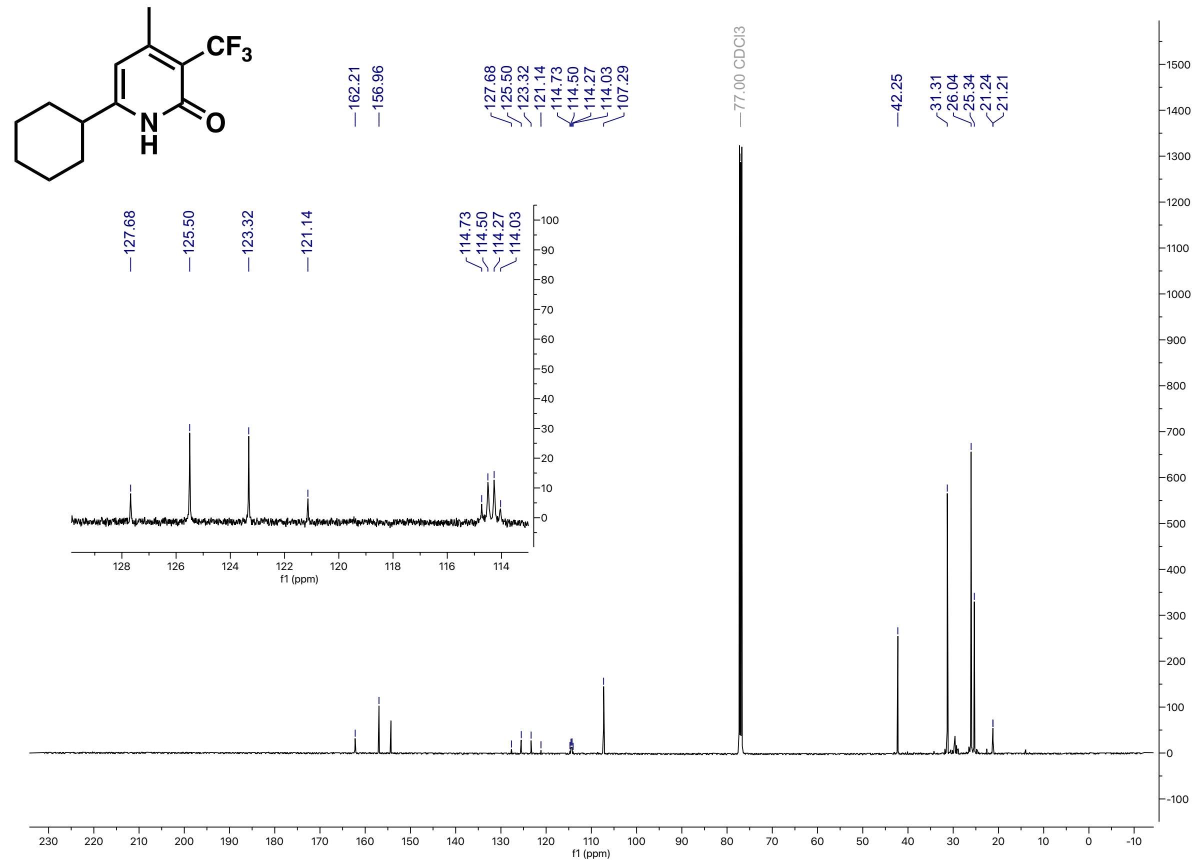

3t – HRMS (ESI)

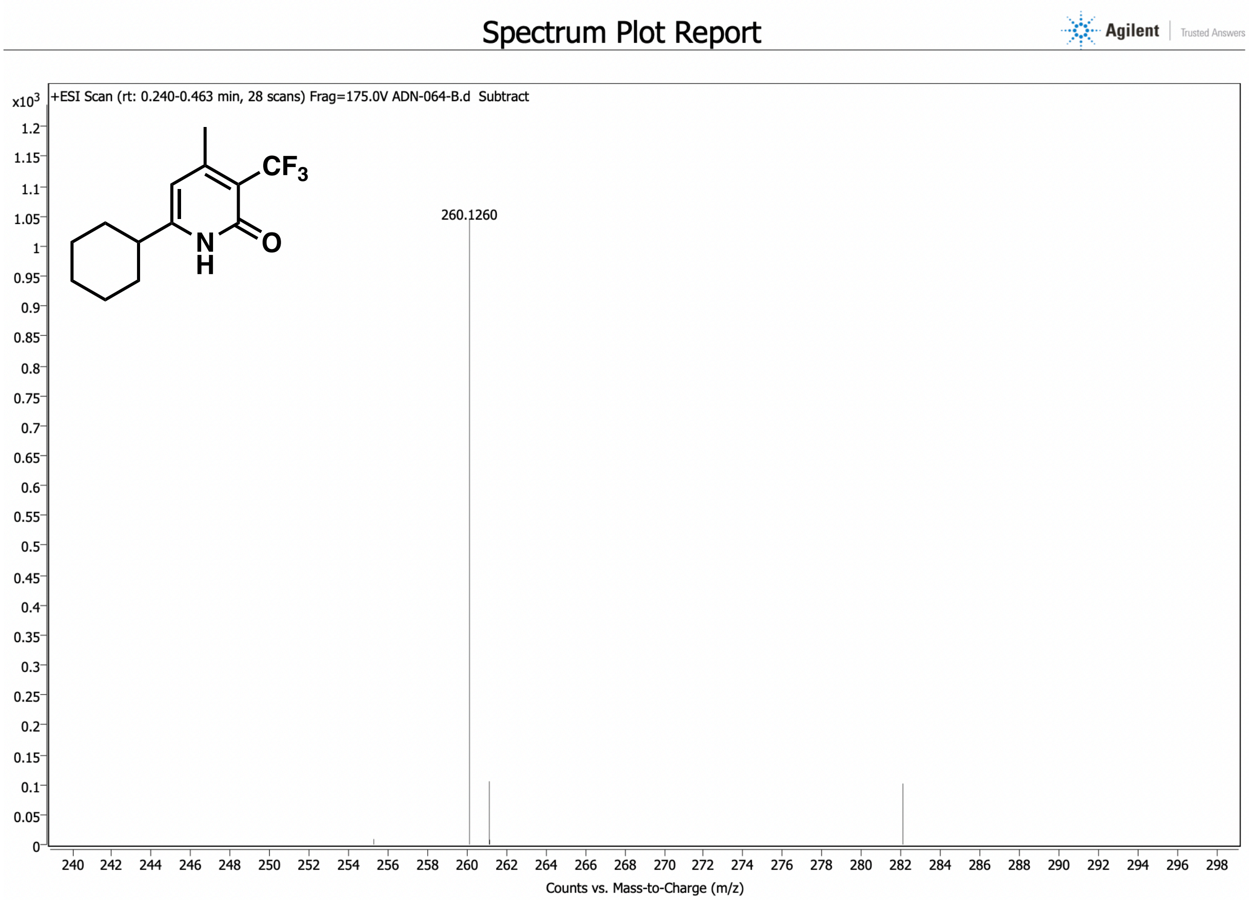

Spectrum Plot Report
Agilent
Trusted Answers
+ESI Scan (rt: 0.240-0.463 min, 28 scans) Frag=175.0V ADN-064-B.d Subtract
x10^3
260.1260
CF3
N
H
O
Counts vs. Mass-to-Charge (m/z)

(3u) [N-Boc Cytisine-CF_3]; tert-butyl (1R,5R)-8-oxo-9-(trifluoromethyl)-1,5,6,8-tetrahydro-2H-1,5-methanopyrido[1,2-a][1,5]diazocine-3(4H)-carboxylate

1u – ^{1}H NMR (400MHz, $CDCl_3$), synthesized by previously published methods (see S18)

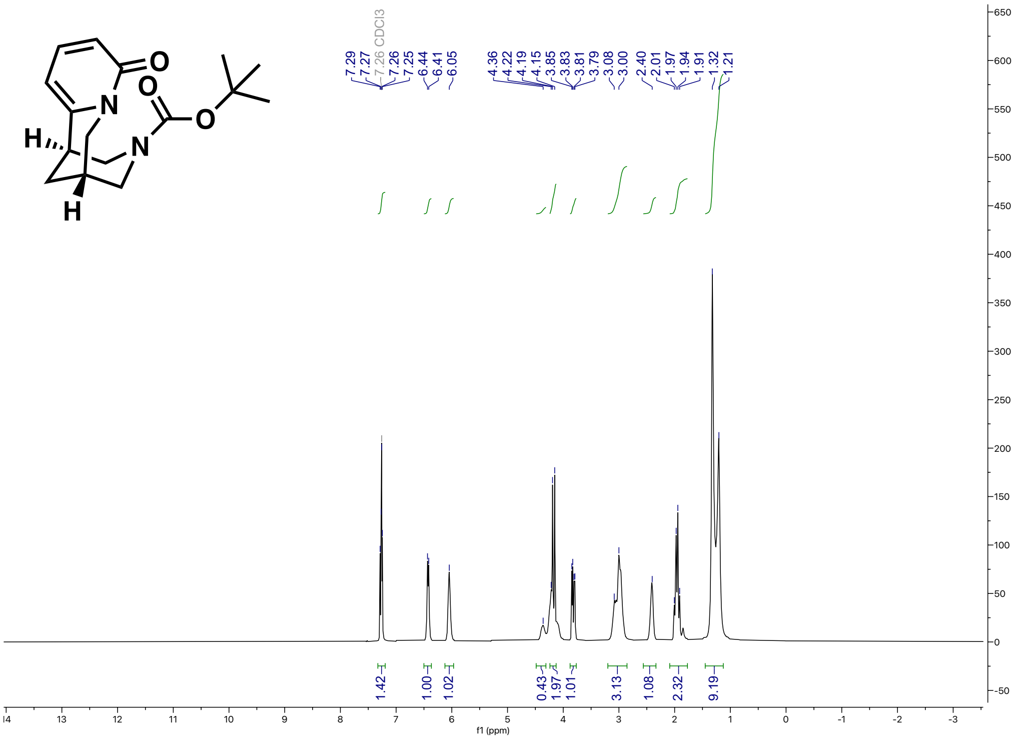

3u – ^{1}H NMR (400 MHz, $CDCl_3$)

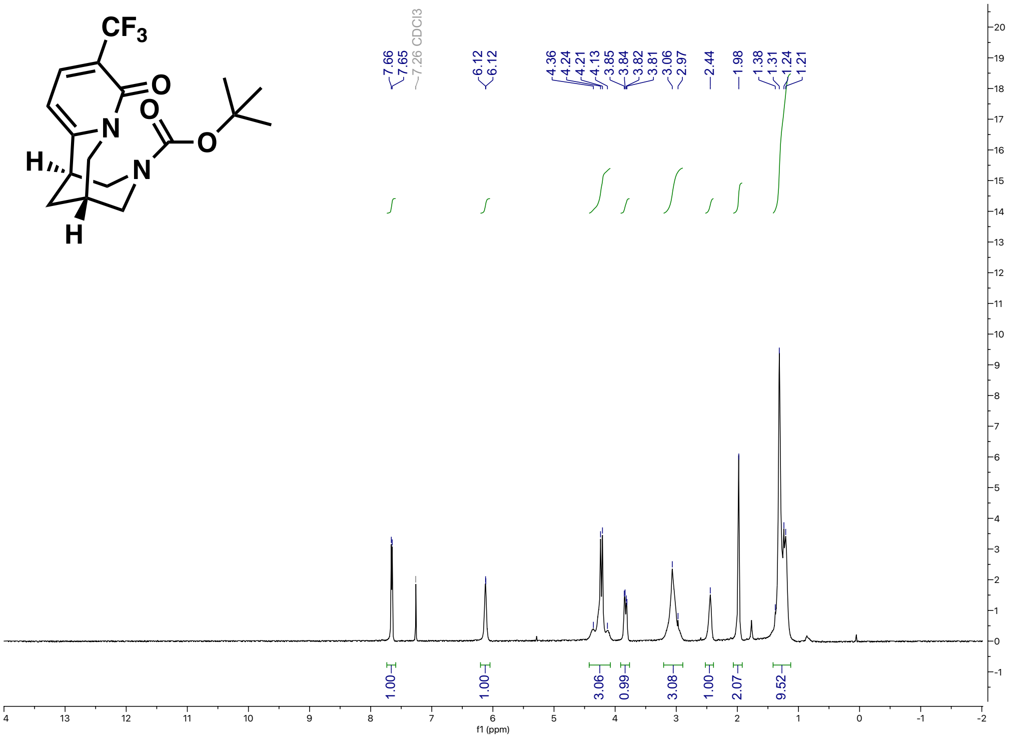

3u – ^{19}F NMR (376 MHz, $CDCl_3$)

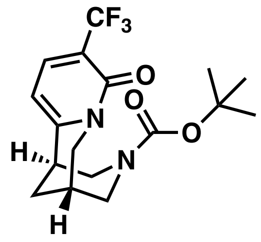

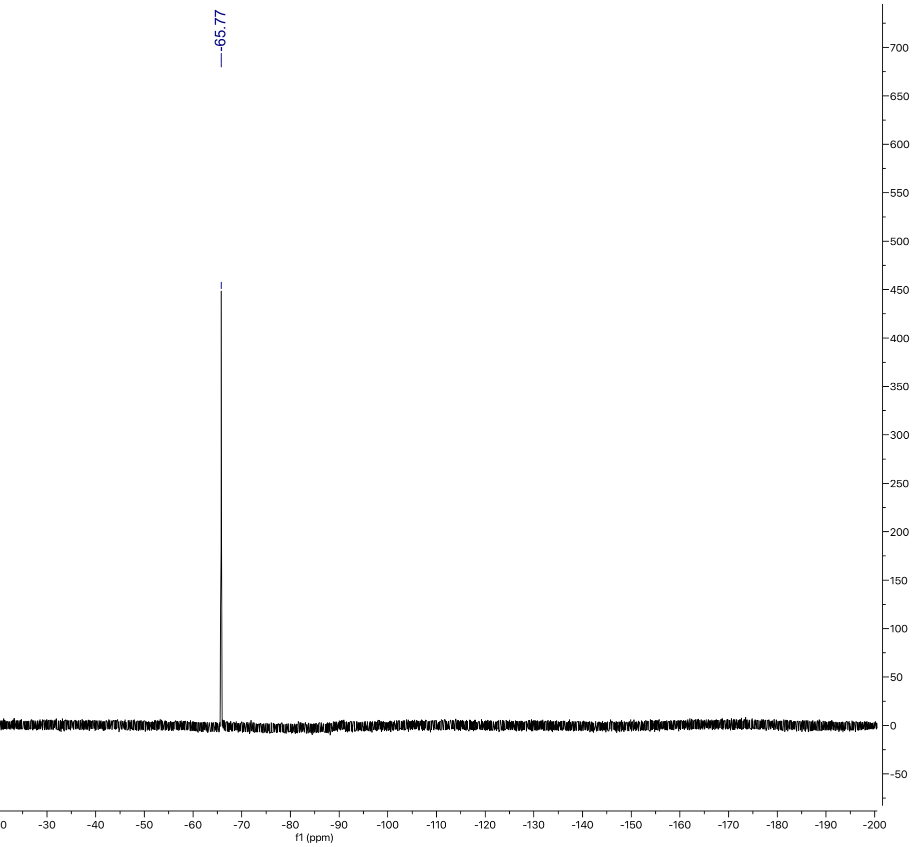

3u – ^{13}C NMR (126 MHz, $CDCl_3$)

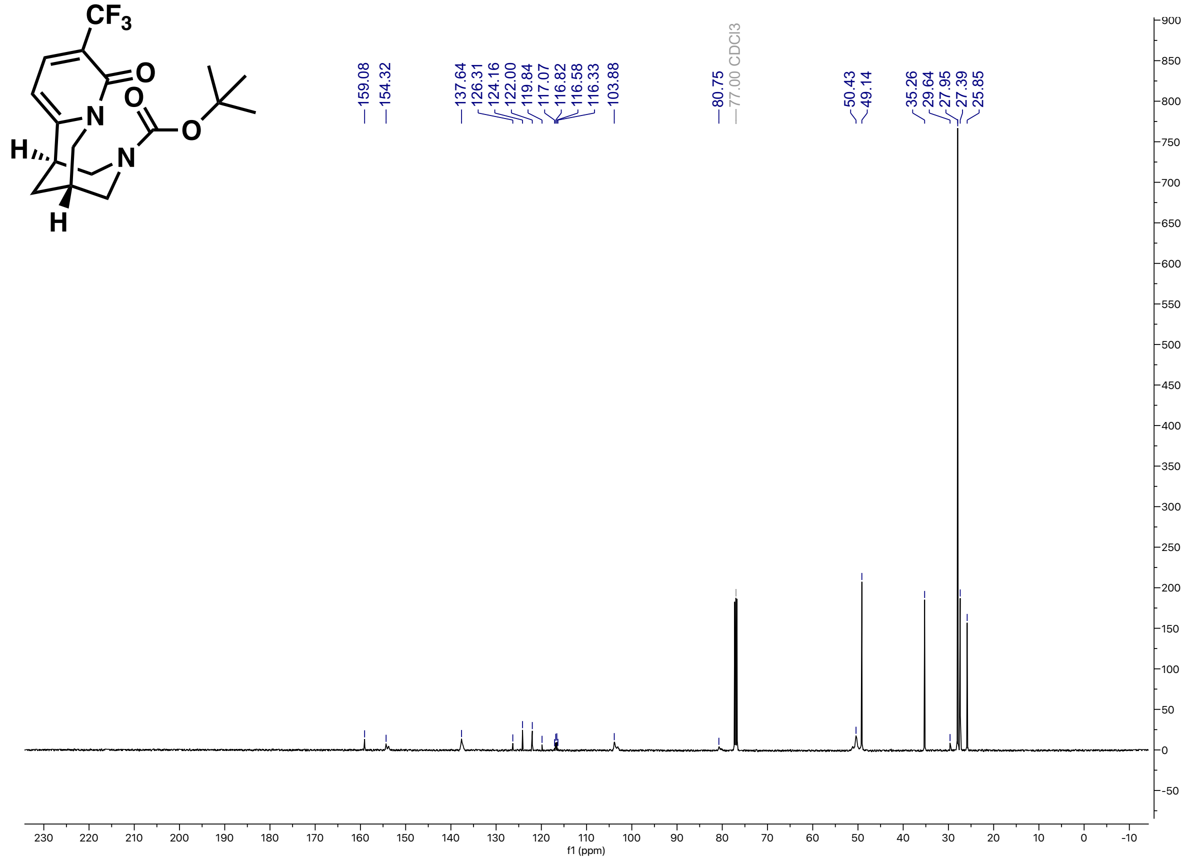

3u – HRMS (ESI)

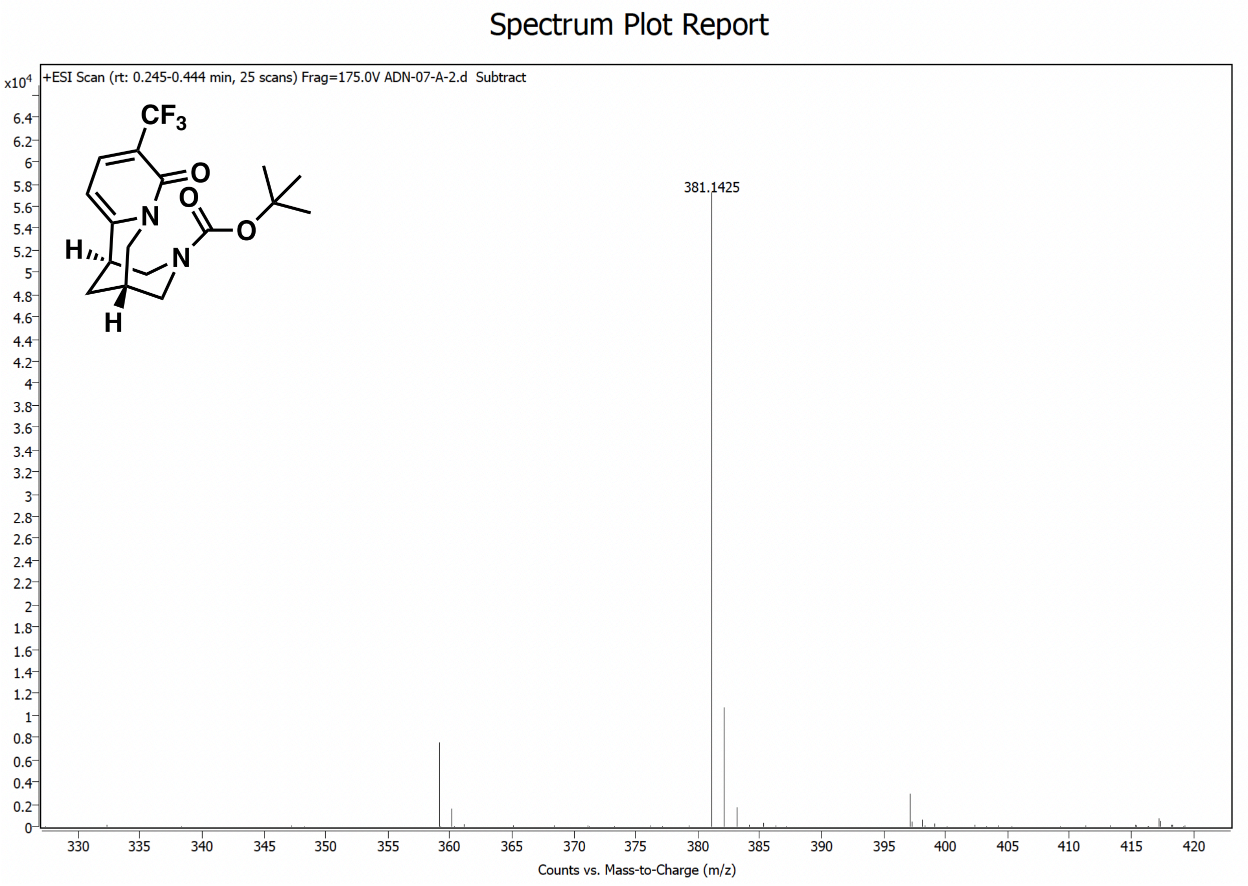

Spectrum Plot Report
x10^4
+ESI Scan (rt: 0.245-0.444 min, 25 scans) Frag=175.0V ADN-07-A-2.d Subtract
CF3
O
O
N
O
N
H
H
381.1425
Counts vs. Mass-to-Charge (m/z)

(3v) [Trifluridine]; 1-((2R,4S,5R)-4-hydroxy-5-(hydroxymethyl)tetrahydrofuran-2-yl)-5-(trifluoromethyl)pyrimidine-2,4(1H,3H)-dione

This compound is also described in *Proc. Natl. Acad. Sci. USA*. **2011**, *35*, 14411-14415.

3v – ^{1}H NMR (400 MHz, MeOD)

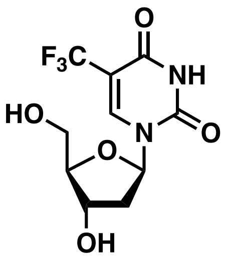

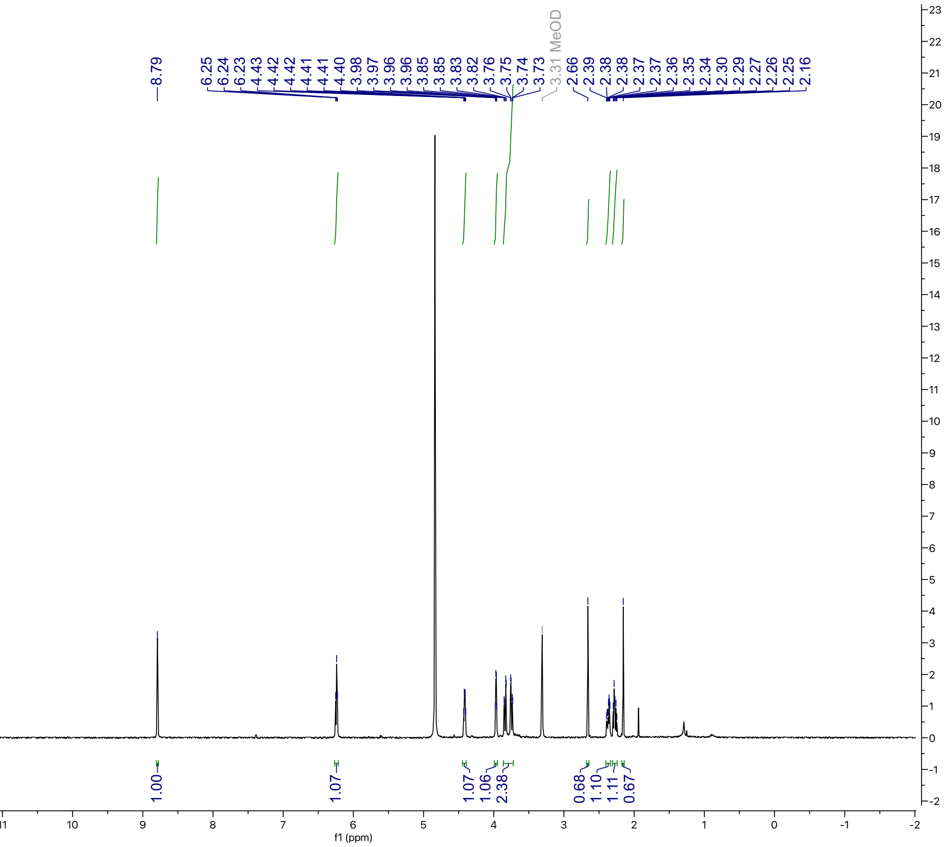

3v – ^{19}F NMR (376 MHz, MeOD)

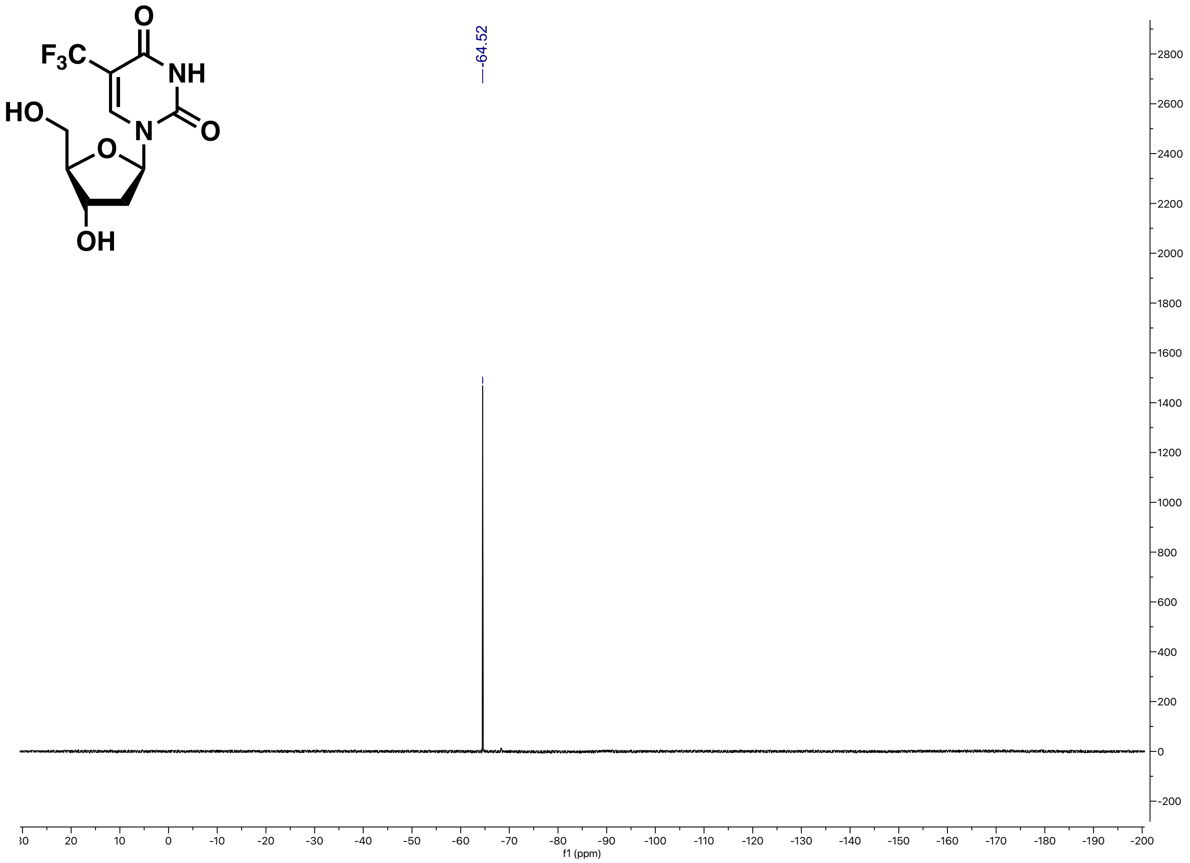

3v – ^{13}C NMR (126 MHz, MeOD)

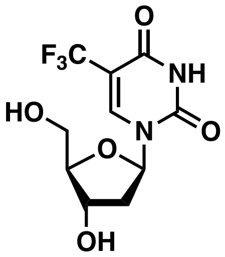

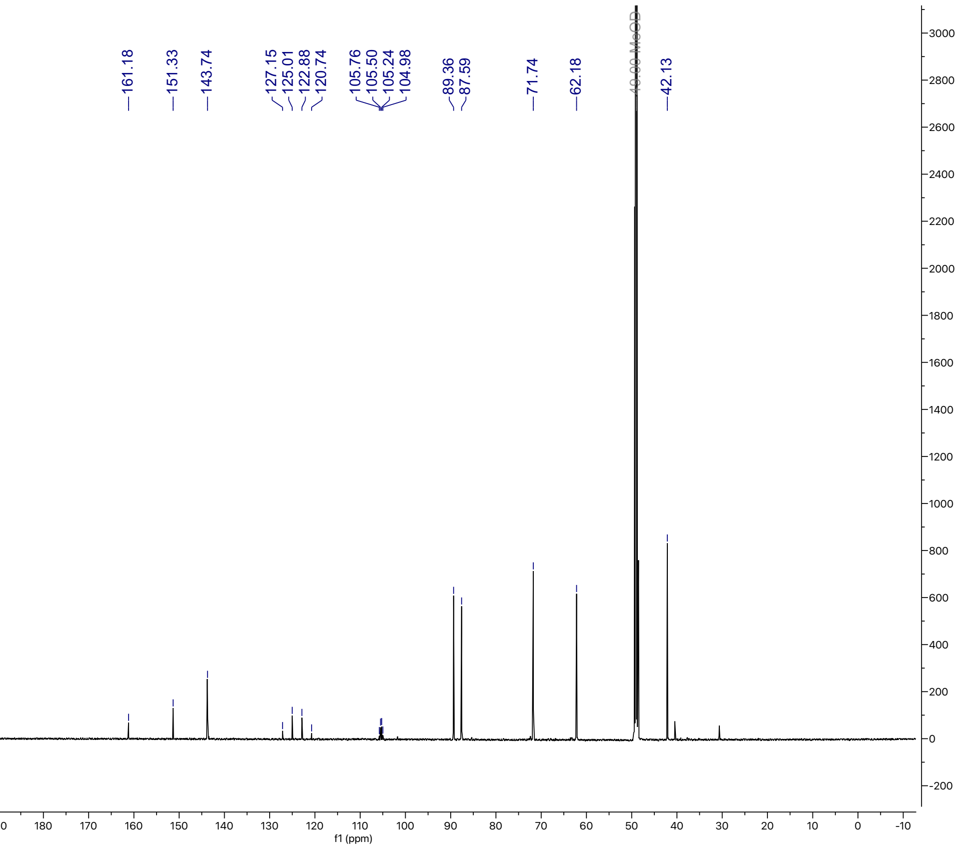

3v – HRMS (ESI)

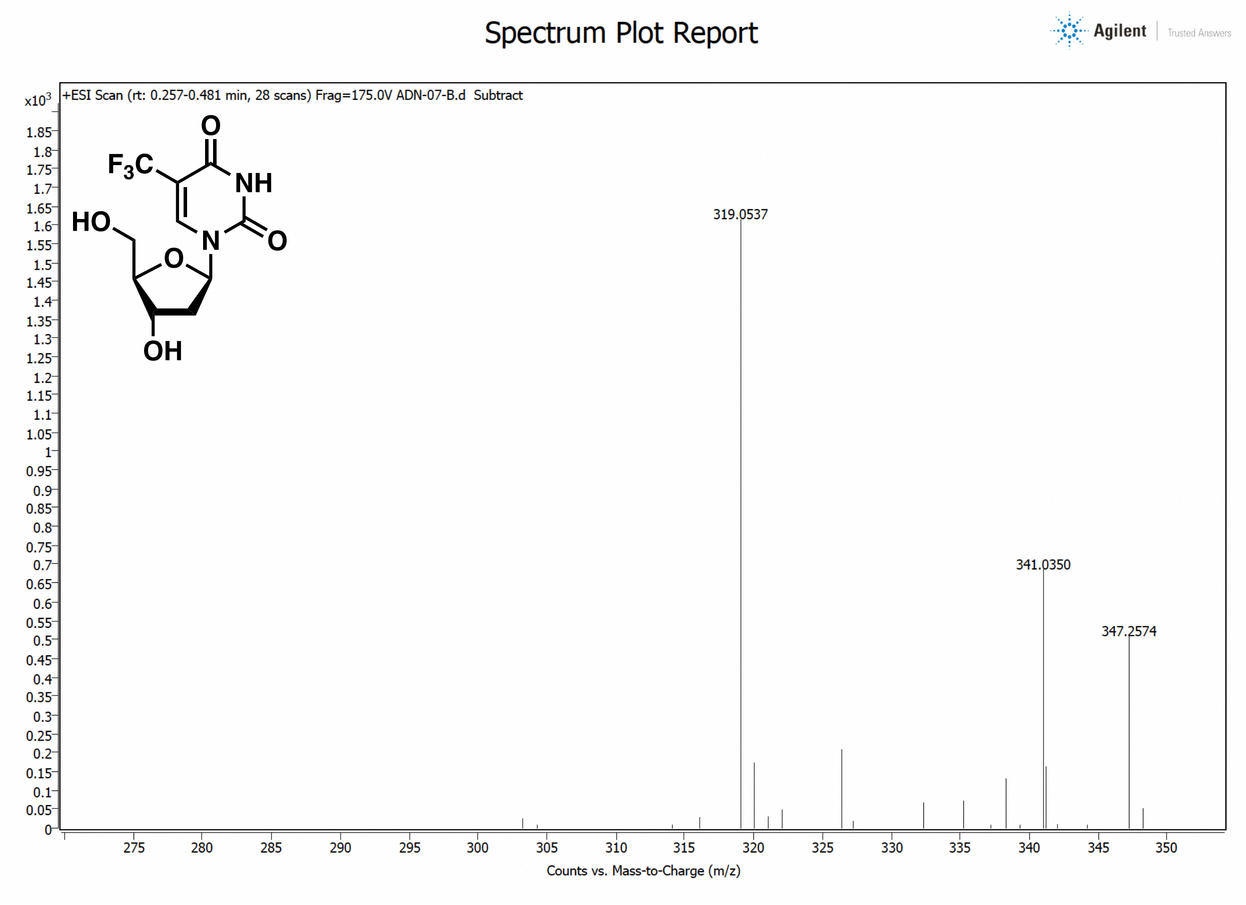

G. Light On/Off Procedure

G1. Preparation of reaction mixture.

2-pyridone (2.97mg, 0.03125mmol) was added to an NMR tube with sodium trifluoromethylsulfinate (9.75mg, 0.0625mmol) in 0.5mL of deuterated DMSO. A sealed 0.03125mmol NMR standard in a capillary tube was also added to the reaction mixture. The mixture was shaken prior to irradiation and after irradiation prior to NMR analyses. When not irradiated, the NMR tube was placed in a cardboard box away from light for the same time period when it was irradiated before NMR analyses. Analyses was done in duplicate initially at 20 minute time intervals, and then changed to 60 minute intervals at 200 minutes total up to 560 minutes.

H. UV-Vis Analysis

H1. General Information regarding equipment/reagents

Measurements were carried out on an Agilent Cary 60 UV-Vis spectrophotometer using a Hellma semi-micro quartz cuvette with a 10mm path length. Analyses were exported as a .csv file and processed using Microsoft Excel. Zero/Background subtraction were corrected prior to running the experiment. Both the cuvette and vials storing solutions were oven-dried prior to use. Analyses were run in MeCN, H_2O, and DMSO, however due to overlapping absorbances in DMSO, deionized H_2O was preferred for analyses.

H2. Preparation of solutions

Stock Reagent Solutions:

0.01M solutions of individual reagents were made and stirred for 15 minutes prior to analyses. Aliquots (19.5μL) were added to 1400μL of deionized water for measurements.

- 0.01M of 2-pyridone (**1a**), 0.02mmol (1.90mg) in 2mL deionized H_2O
- 0.01M of Langlois' Reagent (**2a**), 0.02mmol (3.12mg) in 2mL deionized H_2O
- 0.01M of DMSO, 0.02mmol (1.42 μL) in 2mL deionized H_2O

Reaction Mixtures (RM) [**1a** : **2a** : DMSO, overall concentration 0.0625M, aliquot 3.12μL]:

- 1:1:0 – 0.125mmol of **1a** and **2a**
- 1:1:1 – 0.417mmol of **1a**, **2a**, and DMSO
- 2:1:2 – 0.025mmol of **1a**, 0.5 mmol of **2a** and DMSO
- 2:1:5 – 0.016mmol of **1a**, 0.03125 mmol of **2a**, 0.078125 mmol of DMSO
- Standard reaction conditions – 0.125mmol of **1a** and 0.25mmol of **2a** were added to a vial containing 2mL of DMSO. This mixture was stirred for 15 minutes prior to analysis. An aliquot (1.04μL) was added to 1400μL of deionized water used for measurements.

H3. General UV-Vis procedure

Stock Reagent UV-Vis

A 10mm path length quartz cuvette (oven dried) was charged with an aliquot (19.5μL) of **1a**, **2a**, and DMSO separately in 1400μL of deionized water, respectively. All mixtures were shaken prior to analyses.

Irradiation-Study UV-Vis

A vial was charged with all reagents using standard reaction conditions (see G2, RM) and stir bar. A 10mm path length quartz cuvette was charged with an aliquot (1.04μL) in 1400μL of deionized water. The reaction mixture was then irradiated at 390nm at a 4cm distance, for a set time interval where aliquots (1.04μL) were taken at specific time points and measured.

Titration Study UV-Vis

A 10mm path length quartz cuvette (oven dried) was charged with an aliquot (19.5μL) of **1a**, and 1400μL of deionized water, respectively. Aliquots of 2.6μL of **2a** were added to solution, mixed thoroughly, and measured up to 2 equivalences (39μL total).

H4. UV-Vis Spectra SI-S Figures

SI-S1. UV-Vis of **1a**

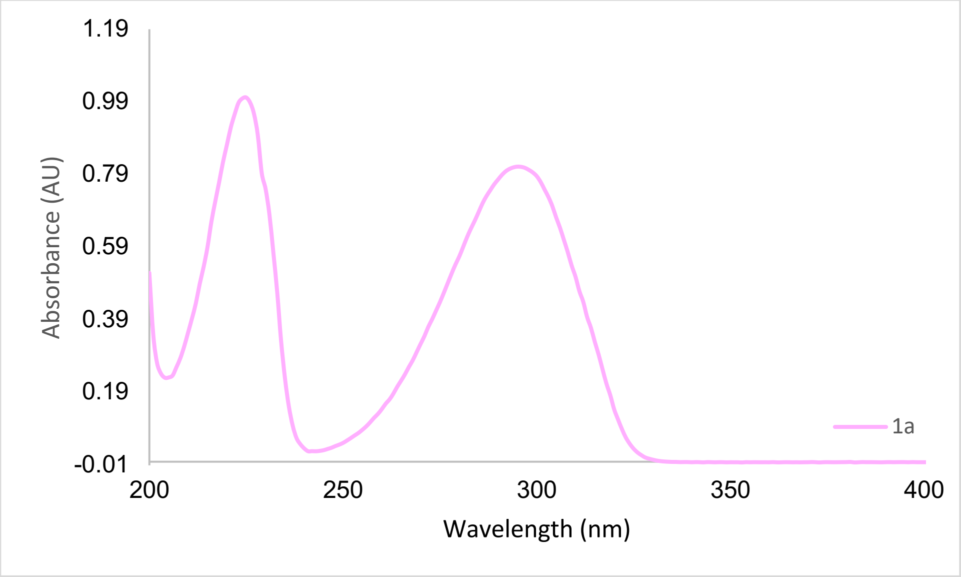

SI-S2. UV-Vis of **2a**

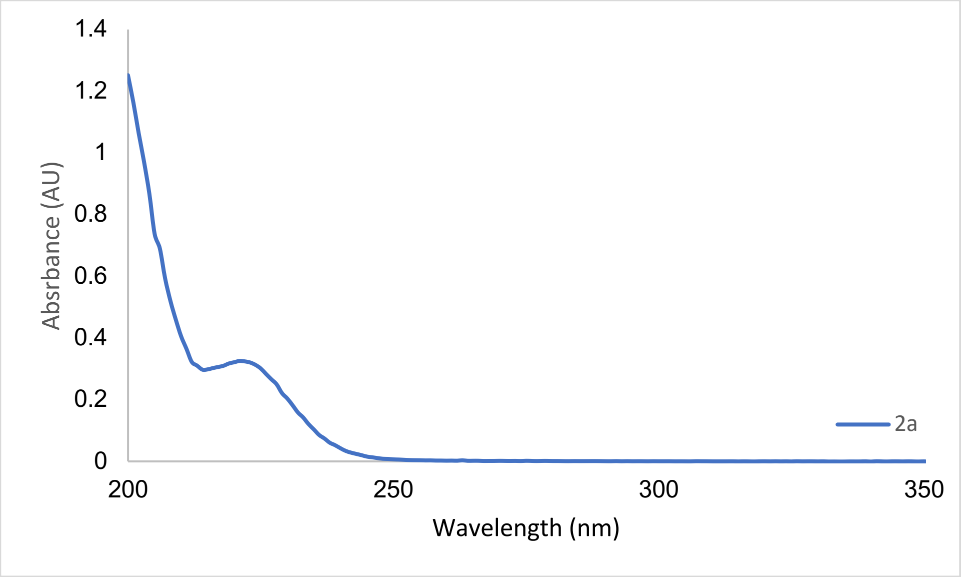

SI-S3. UV-Vis of **DMSO**

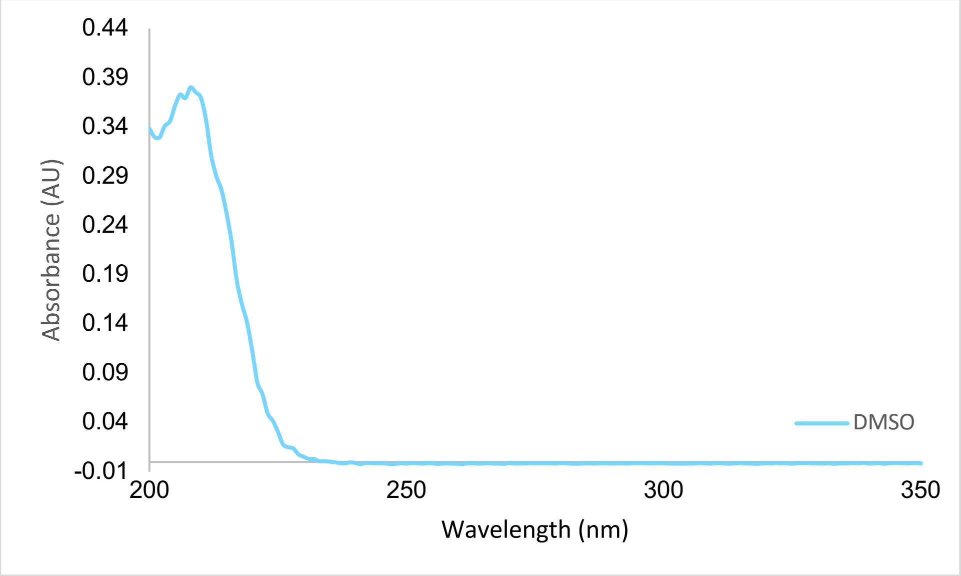

SI-S4. Overlay of reagents

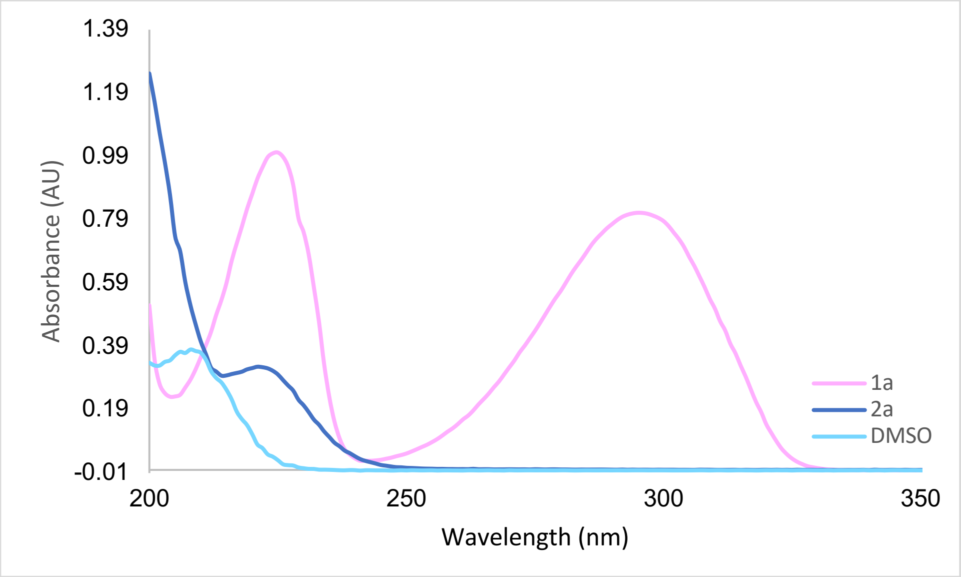

SI-S5. UV-Vis of reaction mixture using standard reaction conditions.

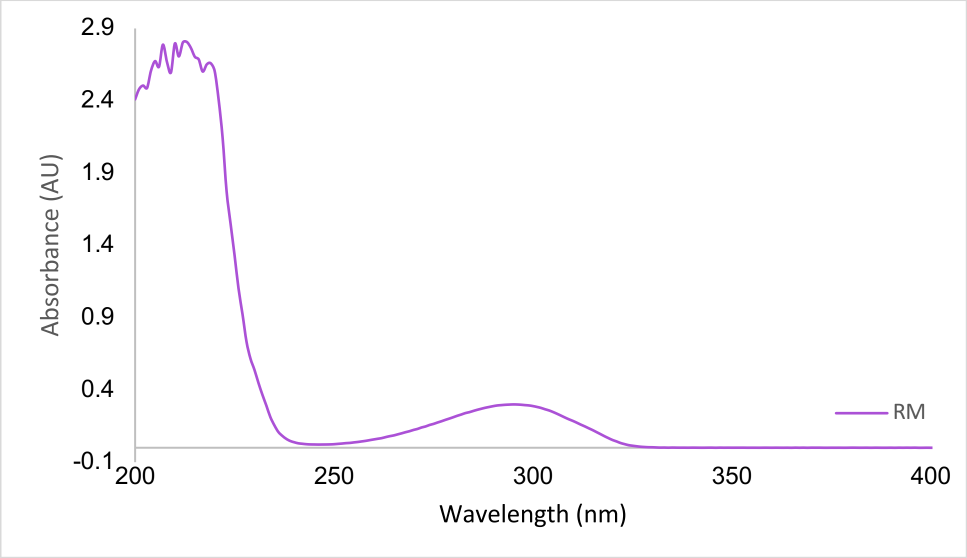

SI-S6. UV-Vis of reaction mixture at different equivalences of reagents (**1a**:**2a**:DMSO).

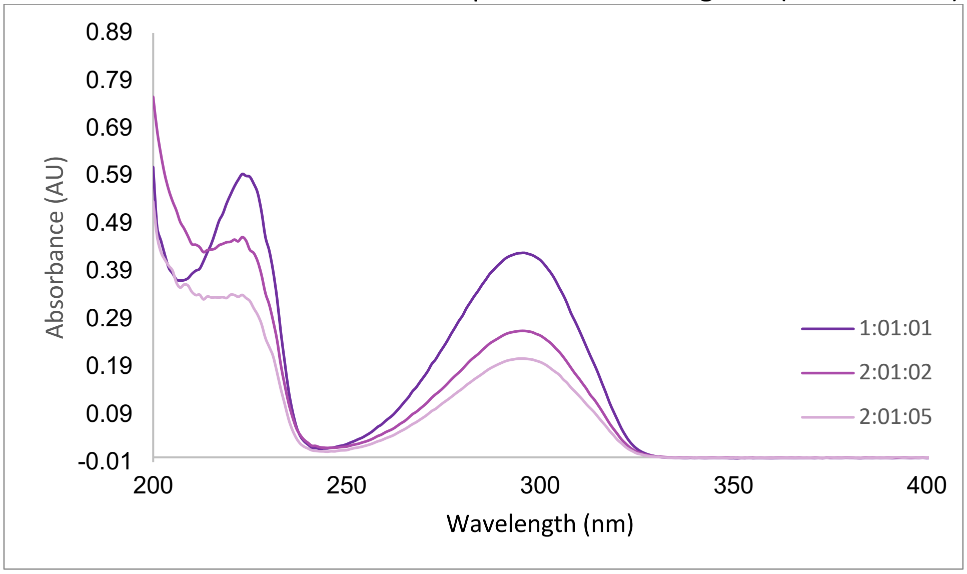

SI-S7. (A) UV-Vis of reaction mixture irradiated in 390 at time points. (B) Magnified at 300-240nm

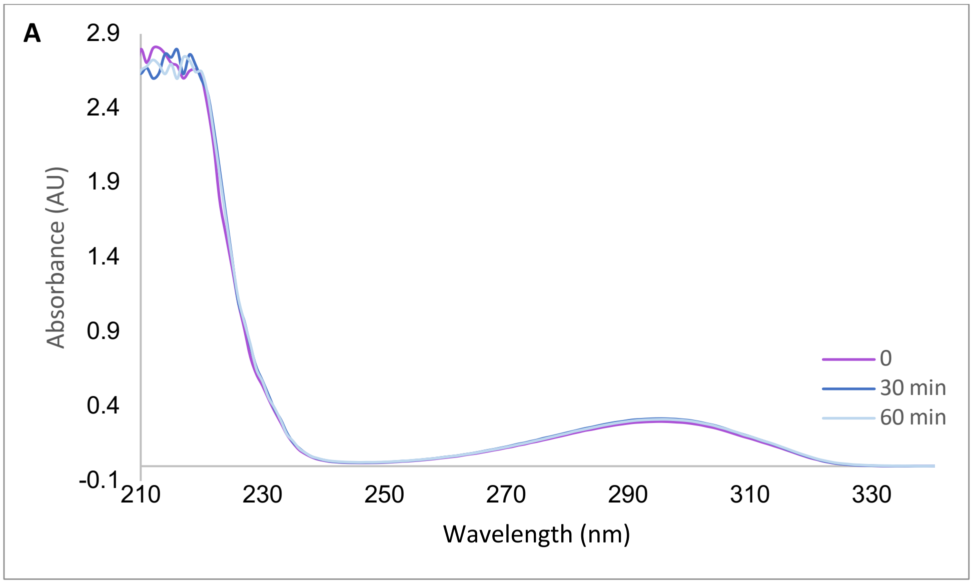

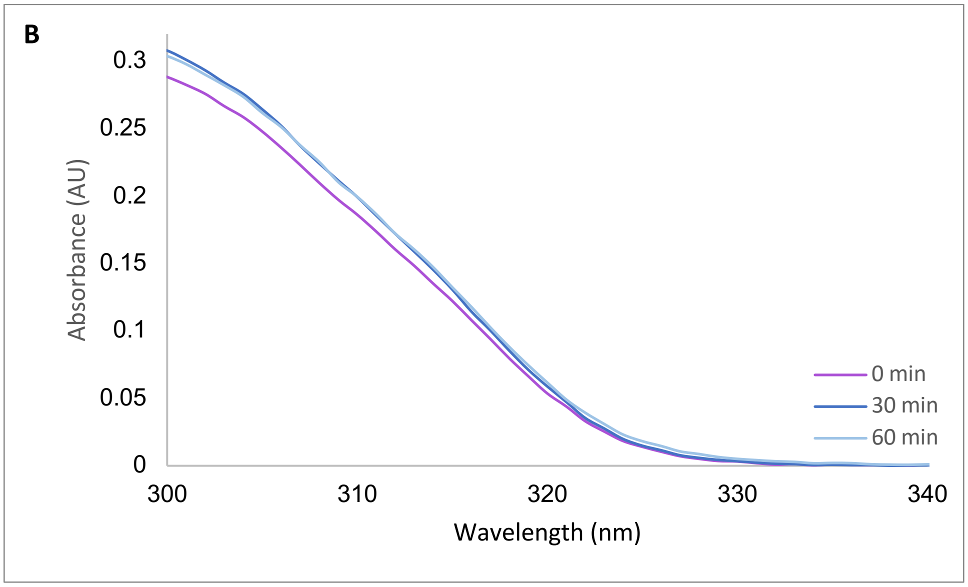

SI-S8. UV-Vis of **1a** titrated with **2a** to 2 equivalences.

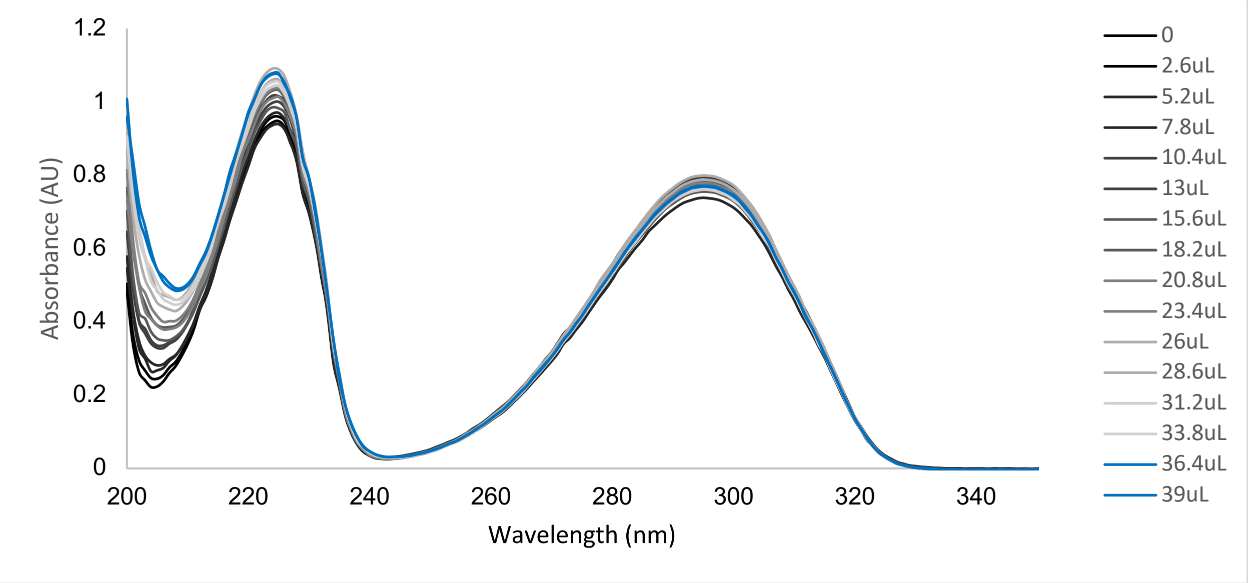

I. Cyclic Voltammetry

I1. General Information regarding equipment/reagents

Electrolyte solution was prepared using NBu_4PF_6 and HPLC grade reagent alcohol. The recrystallized salt was then dried in a Fisher Scientific Isotemp Vacuum Oven (model 280A) equipped with an Emerson Application Vacuum Pump (model S55NXMPF-6788).

The boron-doped diamond (BDD) working electrode was polished using Buehler MicroPolish (part no. 40-10075, batch B112-B-S1) and a small amount of deionized water on a Buehler MicroCloth Polishing Cloth (part no. 40-7212, batch 809-3).

All cyclic voltammograms were performed using a CH Instruments Electrochemical Workstation (model 660E) in a nitrogen-atmosphere Glove Box from Vigor Gas Purification Technologies (model SG1200/750TS) equipped with an Edwards Vacuum Two Stage Rotary Vane Pump (model RV12). Analyses were exported as a .csv file and processed using Microsoft Excel.

I2. Preparation of electrolyte and sample solutions

Approximately 200.0 mL of MeCN was refluxed over CaH_2. It was further dried by siphoning from the storage bottle into an oven-dried, nitrogen-flushed column packed with activated alumina.

To prepare the salt for the electrolyte solution, 14 grams of commercially available tetrabutylammonium hexafluorophosphate were added to a 250.0 mL Erlenmeyer flask. The salt was dissolved by adding 75 mL of 95% reagent alcohol–previously prepared using reagent alcohol and deionized water–and heating the flask in a water bath at 80ºC. Once the salt was dissolved, the flask was removed from the water bath and left on the benchtop to slowly cool to room temperature. The flask was then placed in an ice bath to cool further. The mixture was filtered using a fine fritted funnel, and the crystals were washed with ice cold 95% alcohol and then left to dry under vacuum. The dry crystals were poured into a clean Erlenmeyer flask and the recrystallization process was repeated two more times. After the third recrystallization, the salt was dried in a vacuum oven at 100ºC for 12 hours and then allowed to cool under vacuum. The salt dried in the vacuum oven at 100ºC for another 12 hours, then allowed to cool under vacuum before storage in the glovebox.

Electrolyte solution for analysis was prepared by dissolving 3.875g of the tetrabutylammonium hexafluorophosphate salt into 100.0 mL of dried acetonitrile using a 100.0 mL volumetric flask. Once dissolved, the solution was transferred to a glass amber bottle and a layer of 3Å activated molecular sieves was added to the bottom of the bottle. The solution was allowed to dry over the sieves for 48 hours and then was filtered into a clean and dry glass amber bottle using a PTFE syringe filter with 0.2 micrometer pore size.

Sample Solutions

- **1a** stock solution: 100mM, 0.1mmol of **1a** in 2mL MeCN, aliquot 50μL
- **2a** stock solution: 100mM, 0.1mmol of **2a** in 2mL MeCN, aliquot 50μL
- 50mM = 0.1mmol reagent in 2mL MeCN; aliquot 100μL
- 100mM = 0.2mmol reagent in 2mL MeCN; aliquot 25μL
- 200mM = 0.4mmol reagent in 2mL MeCN; aliquot 50μL

- 400mM = 0.8mmol reagent / 2mL MeCN; aliquot 12.5μL

I3. General voltammetry procedure

A silver wire, a platinum wire, and a teflon electrochemical cell cap were cleaned with deionized water and acetone prior to being moved into the glovebox. BDD electrode was polished with micropolish on a soft polishing pad before also being cleaned with deionized water and acetone. The glass electrochemical cell to be used for cyclic voltammetry was removed from a 105ºC oven and allowed to cool in a desiccator.

A clean and dry electrochemical cell was secured in place using a ring stand equipped with a clamp. The cell was filled with 5.0 mL of the prepared electrolyte solution, and a teflon cap was placed on top of the cell. A glass separate compartment was cleaned with a small amount of electrolyte solution, filled with the electrolyte solution, and then placed into the corresponding hold in the teflon cap. The silver wire reference electrode was inserted into the separate compartment and connected to the white lead. The boron-doped diamond working electrode and platinum wire counter electrode were then inserted into their corresponding holes in the teflon cap and connected to the green and red leads, respectively.

Conditioning of the electrode was performed by cycling through the potential window 2000 times at a scan rate of 10 V/s. Once the background current stabilized, background scans were collected at scan rates of 0.1, 0.2, 0.5, 1.0, 2.0, and 5.0 V/s. After background scans, an aliquot of sample stock solution was added to the cell for a final concentration of 1.0 mM. The sample solution was also scanned at all the scan rates. Each of the sample scans were background subtracted.

Plotting Convention: IUPAC
Electrode Materials:
Working: Boron-Doped Diamond
Counter: Platinum
Reference: Silver

I4. CVs / SI-S Figures

SI-S9. CV of **1a**

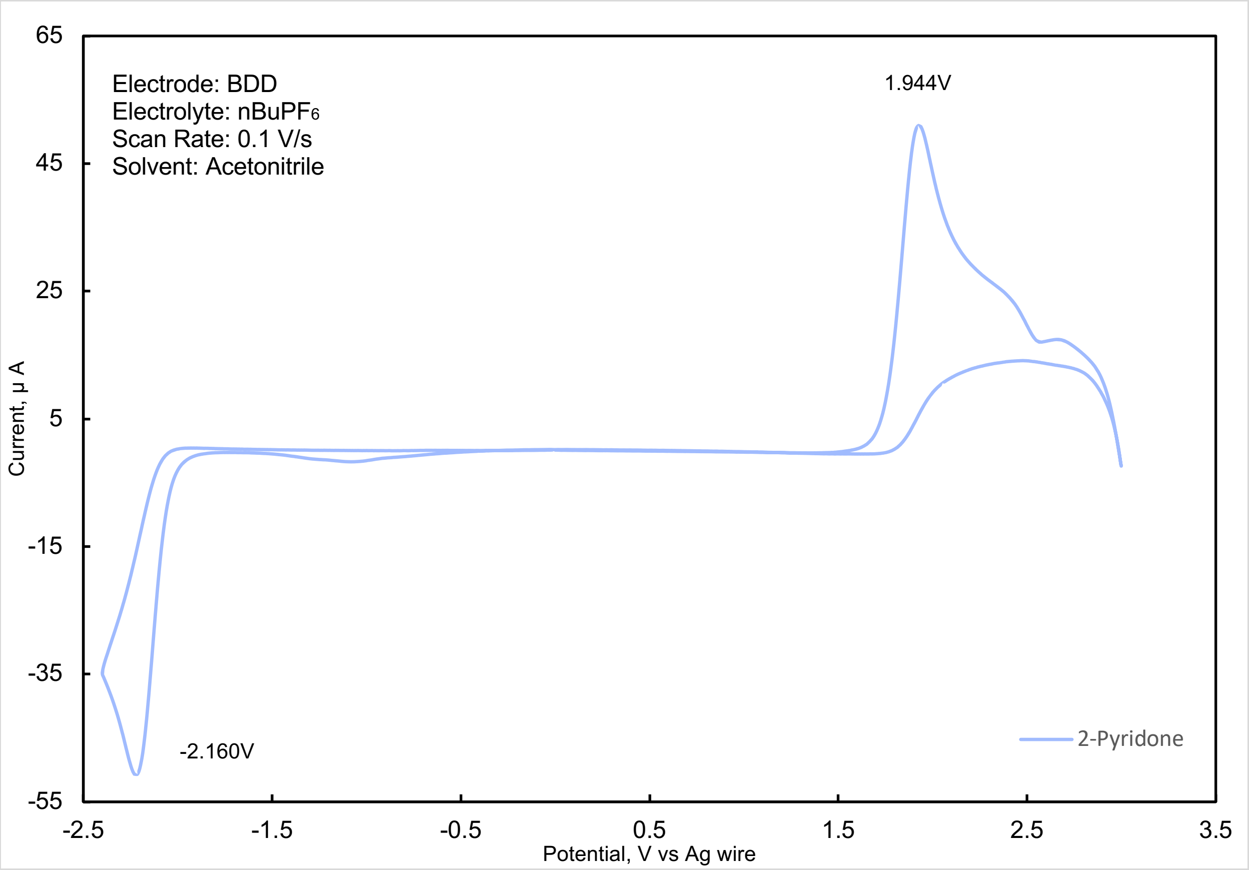

SI-S10. CV of **2a**

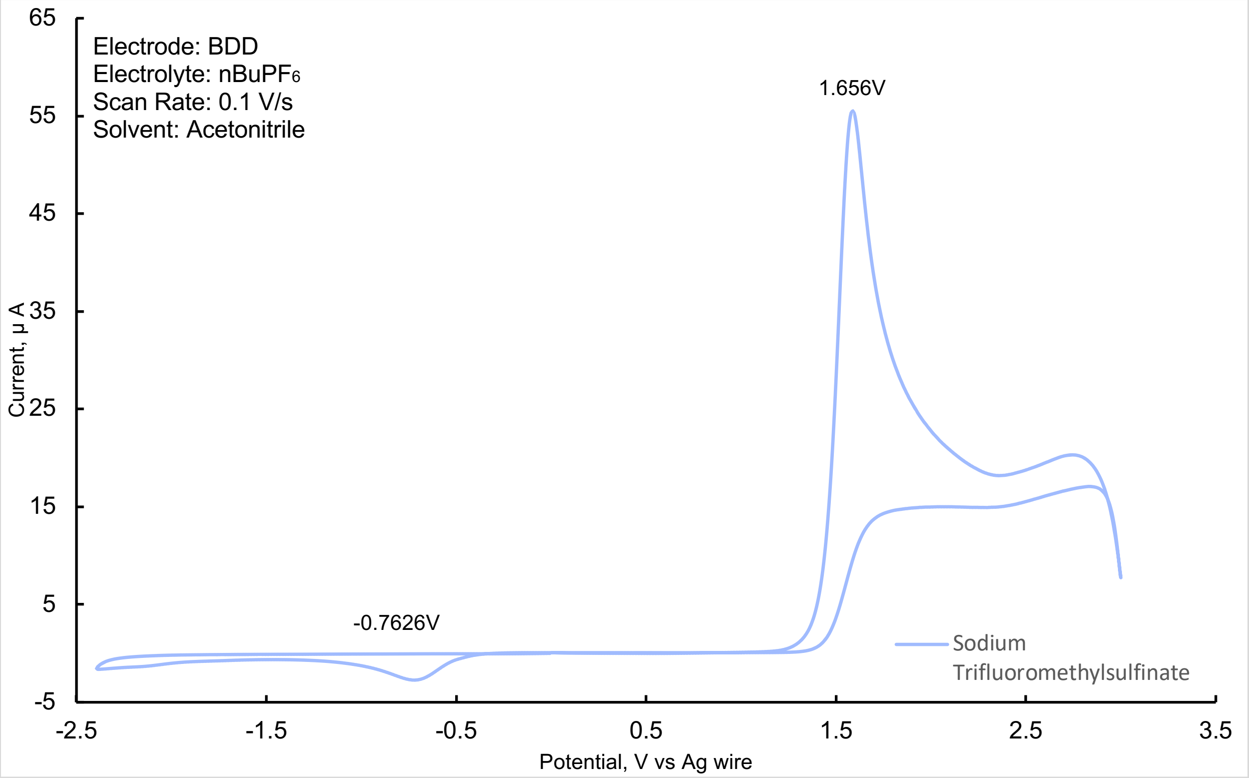

SI-S11. CV of **3a**

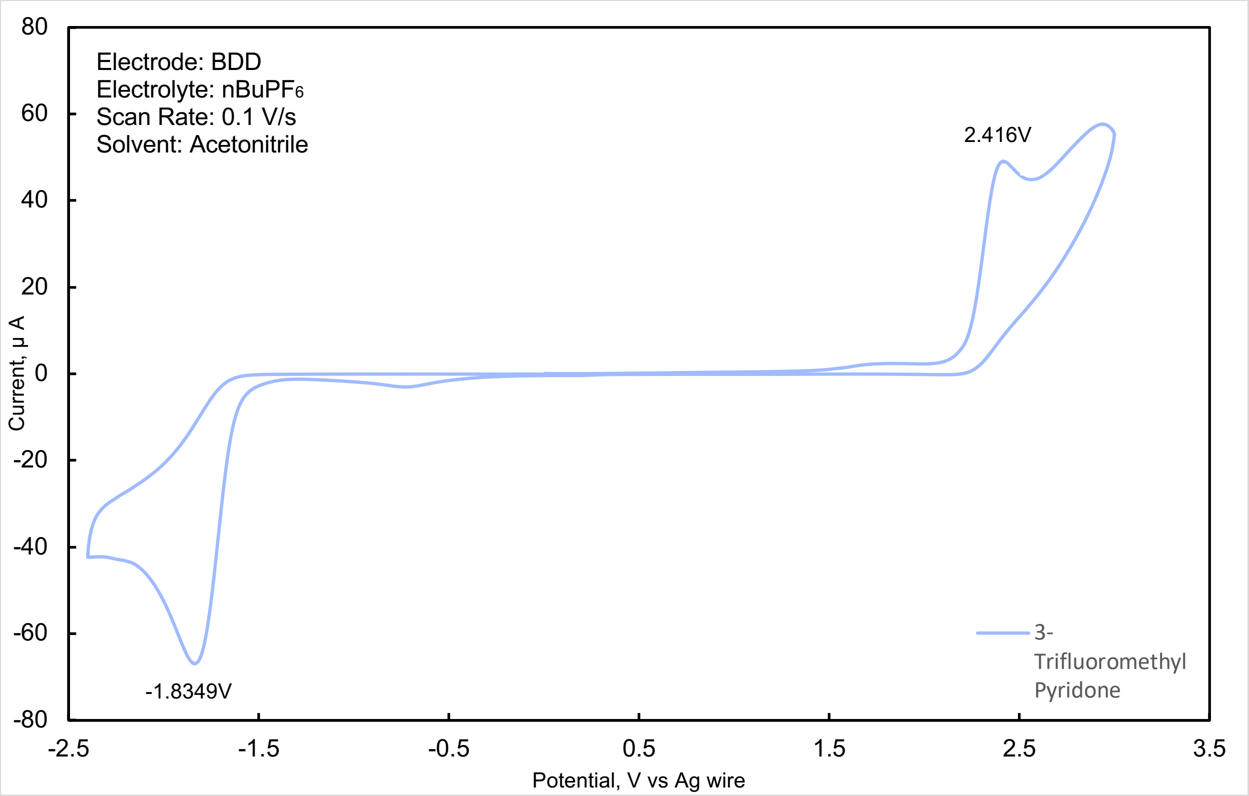

SI-S12. CV of **1a** and **2a** at 1mM, 1:1 equiv., scan rate dependence

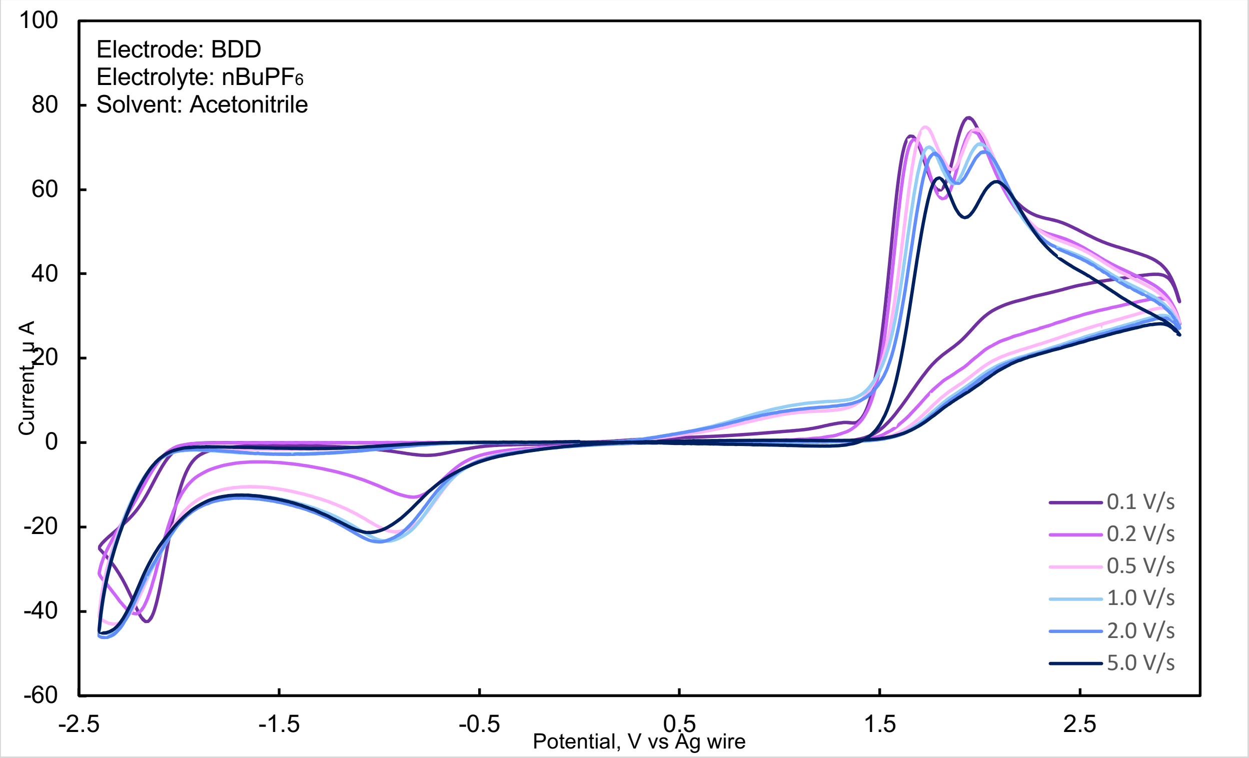

SI-S13. Irradiation interval study of **1a** and **2a** at multiple concentrations irradiated for 1h

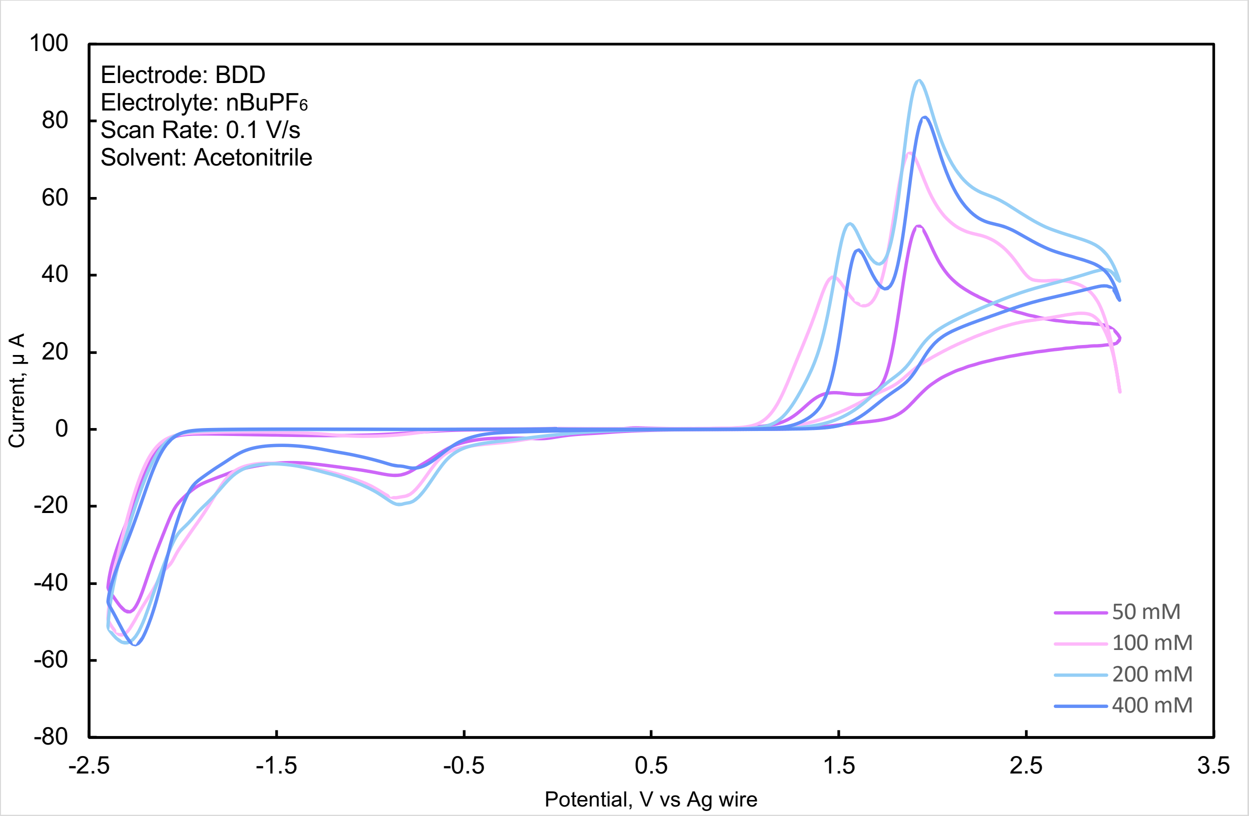

SI-S14. Irradiation interval study of **1a** and **2a** at multiple concentrations irradiated for 7h

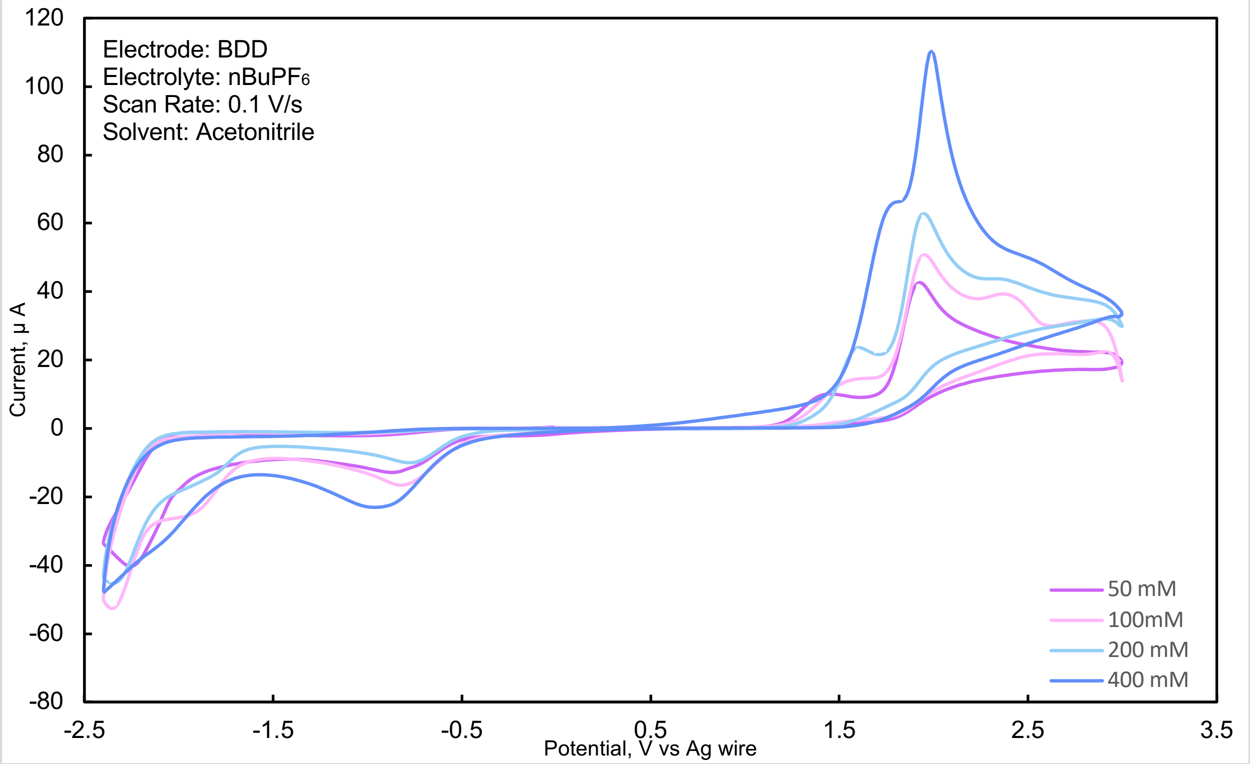

SI-S15. Irradiation interval study of **1a** and **2a** at multiple concentrations irradiated for 15h

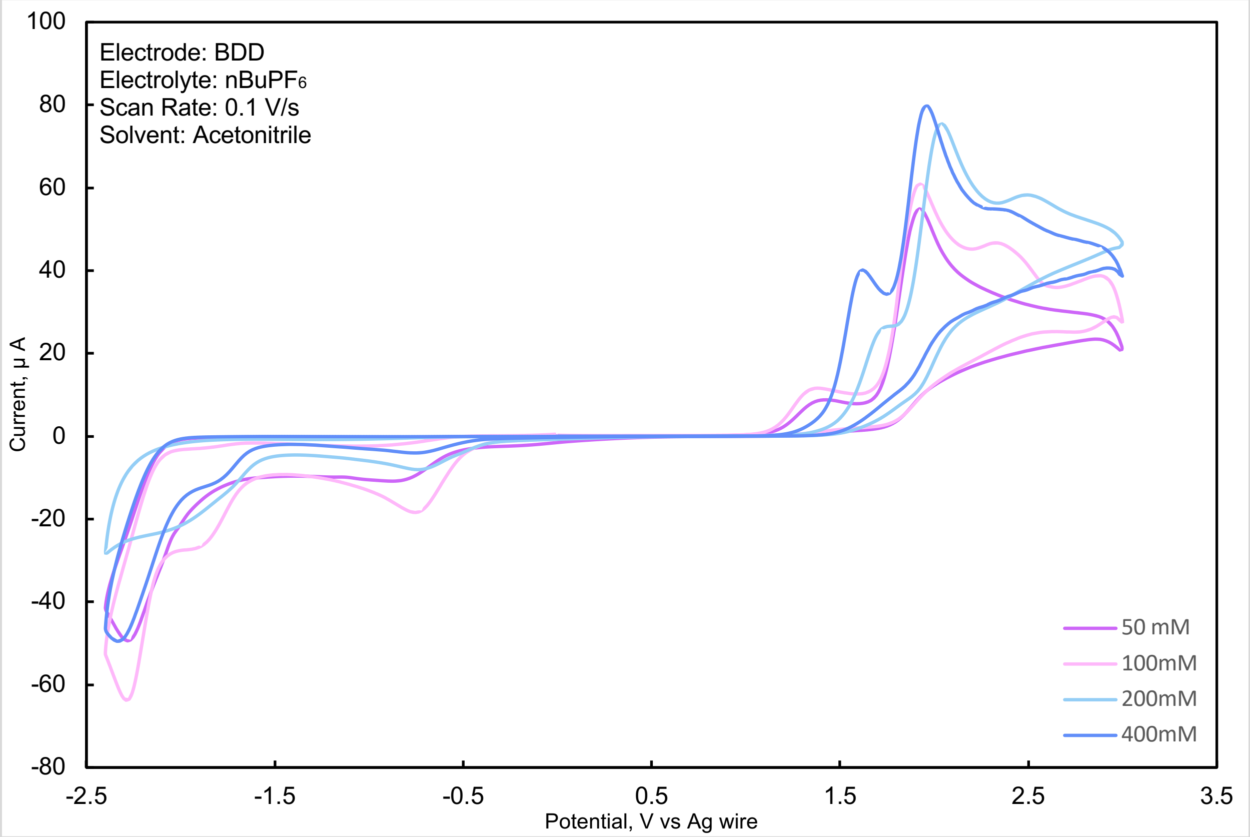

Supplement: Supplementary file 1 — ol3c01710_si_001.pdf [file ol3c01710_si_001.pdf]
